# Long-term decline in above-ground carbon sinks in Brazilian tropical and subtropical forests

**DOI:** 10.1038/s41467-026-74921-0

**Published:** 2026-08-17

**Authors:** Vinícius Andrade Maia, Natália de Aguiar-Campos, Fernanda Coelho de Souza, Cléber Rodrigo de Souza, Jamir Prado-Junior, Camila Laís Farrapo, Tainá Mamede Cirne-Silva, Jean Daniel Morel, Fernanda Moreira Gianasi, Gabriela Gomes Pires de Paula, Nathalle Cristine Alencar Fagundes, Alisson Borges Miranda Santos, Wilder Bento da Silva, Felipe de Carvalho Araújo, Fernanda de Oliveira, André Maciel da Silva Sene, Lidiany Carolina Arantes da Silva, Leony Aparecido Silva Ferreira, Miguel Gama Reis, Denise Moura Madeira, Rafaella Tavares Pereira, Vinvivenci Filipe Pereira de Lima e Silva, Ana Lívia de Carvalho Rodrigues, Renan de Soldi Matzner, Michael de Oliveira Alves Braga, Marco Aurélio Leite Fontes, Ana Carolina Silva, Pedro Higuchi, Rubens Manoel dos Santos

**Affiliations:** 1 Departamento de Ciências Florestais, Universidade Federal de Lavras, Lavras, MG Brazil; 2 Centre for Tropical and Environmental Sustainability Science, College of Science and Engineering, James Cook University, Cairns, QLD Australia; 3 BeZero Carbon, London, UK; 4 Universidade Federal de Uberlândia, Uberlândia, MG Brazil; 5 Departamento de Ciências Biológicas, Universidade Federal de Lavras, Lavras, MG Brazil; 6 Universidade do Estado de Minas Gerais, Ituiutaba, MG Brazil; 7 Departamento de Engenharia Florestal, Universidade do Estado de Santa Catarina, Lages, SC Brazil

**Keywords:** Climate-change ecology, Forest ecology, Biodiversity

## Abstract

Recent evidence indicates that Brazilian forests are shifting from persistent carbon sinks to emerging carbon sources, with important implications for the global climate. To investigate this trend, we analyse long-term census data from 49 forest sites spanning southeast and southern Brazil, including tropical and subtropical forests. We examine above-ground carbon dynamics and its relationship with tree diversity and functional traits that are tightly linked with species’ carbon processing and storage capacities. Our findings reveal a long-term trend of taxonomic, functional and phylogenetic homogenisation and a decline in the forests’ above-ground carbon sink. Specifically, homogeneous forests have lower carbon losses, whilst forests with more conservative species are associated with greater carbon gains and lower carbon losses. Overall, our study establishes a baseline to understand and predict the above-ground carbon sink in the face of ongoing climate change in this region.

## Abstract (web summary)

Brazilian forests are increasingly shifting from carbon sinks to sources. Analysis of 49 sites shows declining carbon sinks and diversity, with forests dominated by conservative species gaining more carbon, highlighting drivers of forest dynamics.

## Introduction

The effects of climate change on tropical forest carbon sequestration are complex. While the above-ground carbon sink of Amazon forests has been declining towards an expected zero in 2035^1^, drier seasonal forests may have already turned into carbon sources^2^. To fully understand these effects and predict future scenarios, we must consider the different ecological dimensions that affect carbon uptake, such as tree species diversity and functional trait composition^3–6^. In the Amazon, some studies have found a positive effect of diversity on forest resilience to climate change^4,7–9^. However, these relationships have been rarely explored on a comprehensive scale in other Brazilian forests, such as the Atlantic Forest^10^ (157,200 km^2^) and Caatinga dry forests^11^ (850,000 km^2^), which experience lower water availability and different trait-productivity relationships^12,13^.

Tree community responses to climatic oscillations and extreme weather events linked to climate change depend on the physiological reactions of trees to varying conditions of water availability and temperature^1,9,14^. Lower rainfall and higher climatic water deficit (CWD) reduce the amount of water available for plant survival and growth, which may ultimately lead to reductions in forest carbon sinks and stocks^9,14,15^. Plant responses to climate are also mediated by soil texture and fertility^2,16^, with complex outcomes due to different responses in above- and below-ground biomass productivity^17^. Overall, higher nutrient availability, particularly of phosphorus, is expected to increase forest productivity^16,17^. Temperature effects are also complex: while net productivity is positively correlated with temperature at the global scale^18^, climate change has been pushing tropical forests to critical temperature thresholds beyond which photosynthesis is impaired^19^.

Other than environmental factors, ecological aspects such as tree species diversity and functional trait composition also play an important role in above-ground carbon dynamics^3,5,12,13^. Evidence points to diverse communities having higher resource-use efficiency due to niche complementarity effects^14,20–23^. Over time and under fluctuating environmental conditions, these effects are hypothesized to result in stable carbon dynamics (i.e., insurance hypothesis)^24^. Following this rationale, taxonomic diversity alone may not accurately reflect niche differentiation due to potential niche overlap among co-occurring species (i.e., functional redundancy)^12^. Phylogenetic and functional diversity, on the other hand, may serve as better proxies for niche complementarity^8,23^. In contrast, in less diverse communities where niche complementarity effects are minimal, the functional composition of the most abundant species may better predict community productivity (i.e., mass-ratio effects)^25^. Although mass-ratio effects operate in high-diversity communities^26^, they have greater weight in low-diversity communities, reflecting the optimal traits that promote growth and survival in a given habitat^12^.

Among these traits, specific leaf area (SLA) and wood density (WD) are often linked to forest productivity due to their role in tree survival and growth^3,4,26–29^. They also help to identify underlying ecological strategies related to resource use and carbon assimilation in trees. Low WD and high SLA indicate an acquisitive strategy, usually linked to fast-growing and short-lived species with high carbon assimilation rates^12,26,30–32^. Although acquisitive species may be vulnerable to hydraulic failure, stem breakage, and herbivory^12,26,33,34^, many are able to endure high temperatures and low humidity by shedding their leaves during the dry season (i.e., deciduousness), particularly in nutrient-rich environments^35^. Such adaptation could theoretically increase the survival of acquisitive deciduous species under a hotter and drier climate, despite evidence that tropical deciduous forests have had higher tree mortality than wetter forests in recent years^2^. On the other end of the spectrum, high WD and low SLA indicate a conservative strategy, usually linked to slow-growing species less prone to hydraulic failure and carbon starvation^12,31,36,37^. These conservative traits not only confer resilience to water-limited conditions but also contribute to higher above-ground carbon stocks and productivity in such environments^12,38^. In fact, because of climate change and increasing droughts, forests are likely to move towards a more conservative trait composition (functional homogeneity), resulting in lower taxonomic and phylogenetic diversity^4,23,28^.

**Fig. 1:**
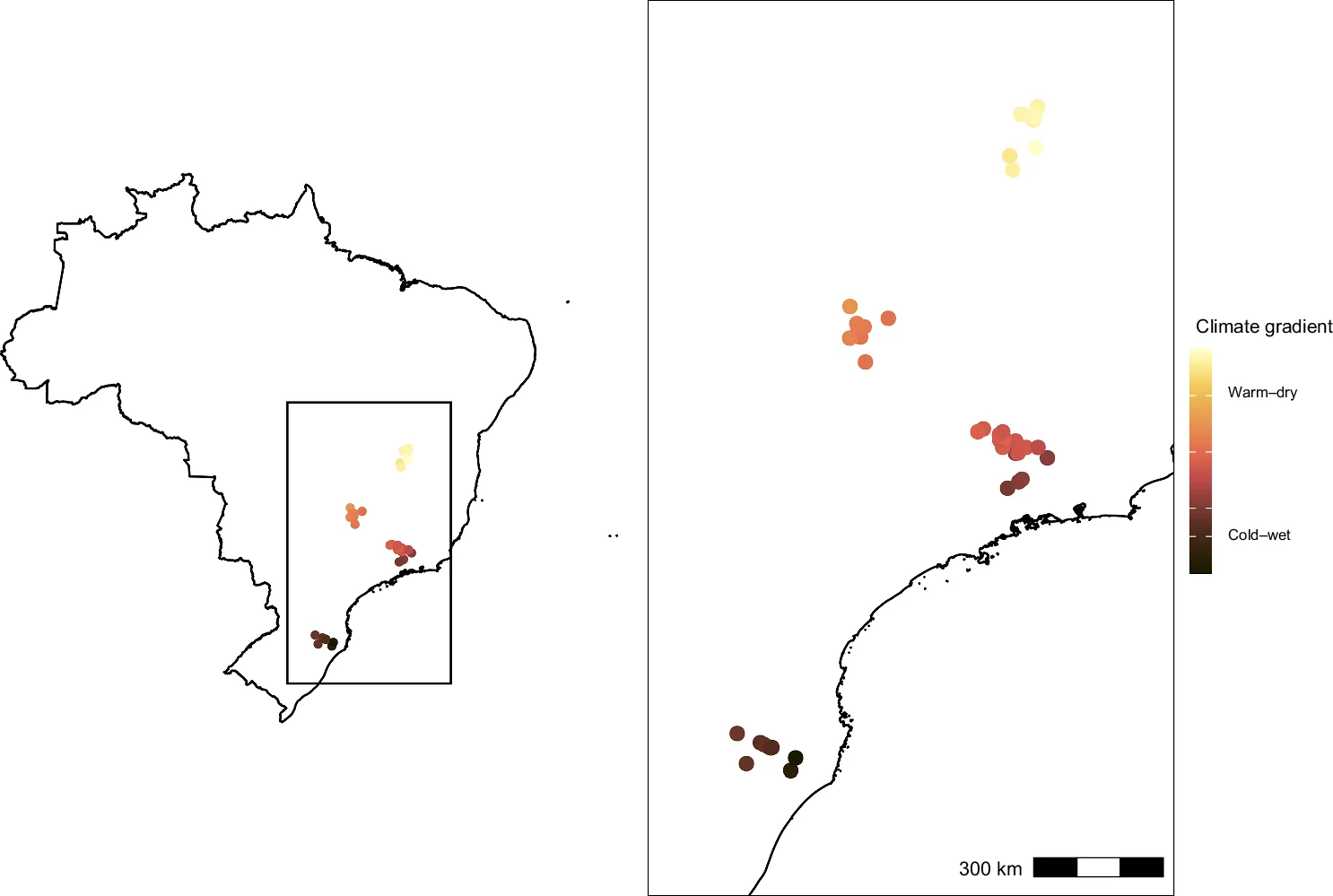
Spatial location of the sampled sites (*n* = 49) in Brazil. Points are coloured by the sites’ climate (gradient obtained from PCA). Colours represent the gradient from cold-wet sites (low values; dark red-brown tones) to warm-dry sites (high values; yellow to pale-yellow tones). Map file obtained from Instituto Brasileiro de Geografia e Estatística (IBGE) available at: https://www.ibge.gov.br/geociencias/organizacao-do-territorio/malhas-territoriais/15774-malhas.html?=&t=acesso-ao-produto. Source data are provided as a Source Data file.

Here, we use a long-term dataset of 49 forest sites in south and southeast Brazil spanning up to 31 years of monitoring, over 50 ha of samples and 942 tree species (Fig. 1). We investigate the temporal trends of different facets of diversity and functional trait composition (hereafter, ecological variables) and their effects on above-ground carbon stocks and dynamics. We show that above-ground carbon stocks and sinks have declined since ~2013, with more rapid reductions in the warm-dry sites than in the cold-wet sites along the gradient. In parallel, taxonomic, functional and phylogenetic diversity show a long-term decline across the whole gradient, indicating functional homogenisation, which is also reflected in narrowing ranges of SLA and WD. Functionally homogeneous forests exhibit lower carbon losses, while a more conservative functional composition is associated with greater carbon gains and lower carbon losses. Our findings establish a baseline to understand and predict the above-ground carbon sink of extra-Amazonian tropical and subtropical forests in Brazil.

## Results

### Long-term trends

**Fig. 2:**
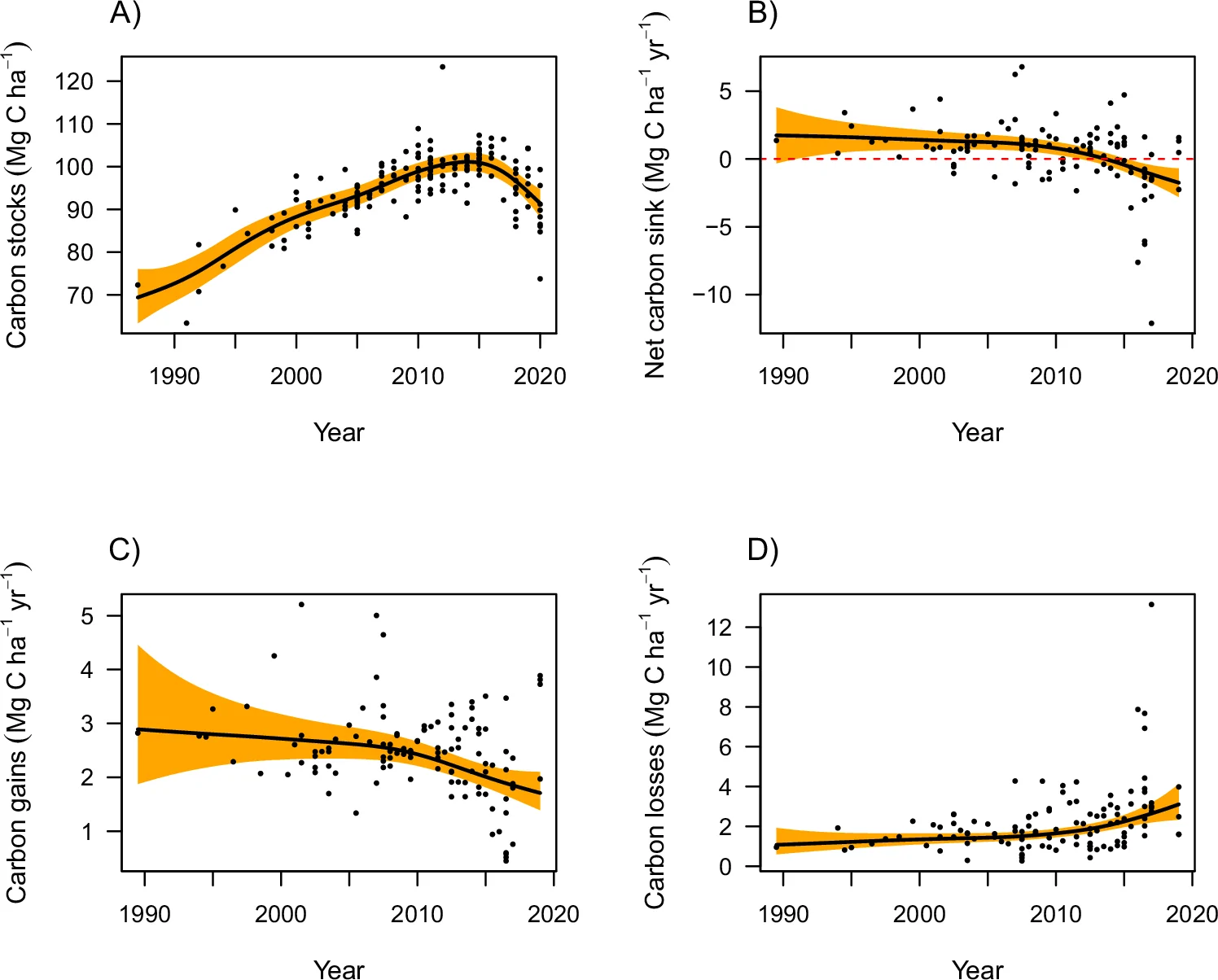
Long-term trends of above-ground carbon dynamics variables. Curves are smooth splines fitted with generalized additive models (GAM). Points are partial residuals after accounting for the random effect of site (*n *= 49), corresponding to each census for above-ground carbon stocks and to each census interval for above-ground carbon dynamics variables. Shaded areas represent 95% confidence intervals. Statistical significance of the smooth terms was assessed using two-sided F-tests, with corresponding *p*-values reported in the legend and in Supplementary Table S1. **A** Above-ground carbon stocks (*n* = 163; F(5.27, 6.37) = 22.79, *p* < 0.001), (**B**) net above-ground carbon sink (*n* = 114; F(2.88, 3.64) = 9.05, *p* < 0.001), (**C**) above-ground carbon gains (*n *= 114; F(2.25, 2.81) = 5.37, *p* = 0.004), and (**D**) above-ground carbon losses (*n* = 114; F(2.56, 3.21) = 8.77, *p* < 0.001). Source data are provided as a Source Data file.

**Fig. 3:**
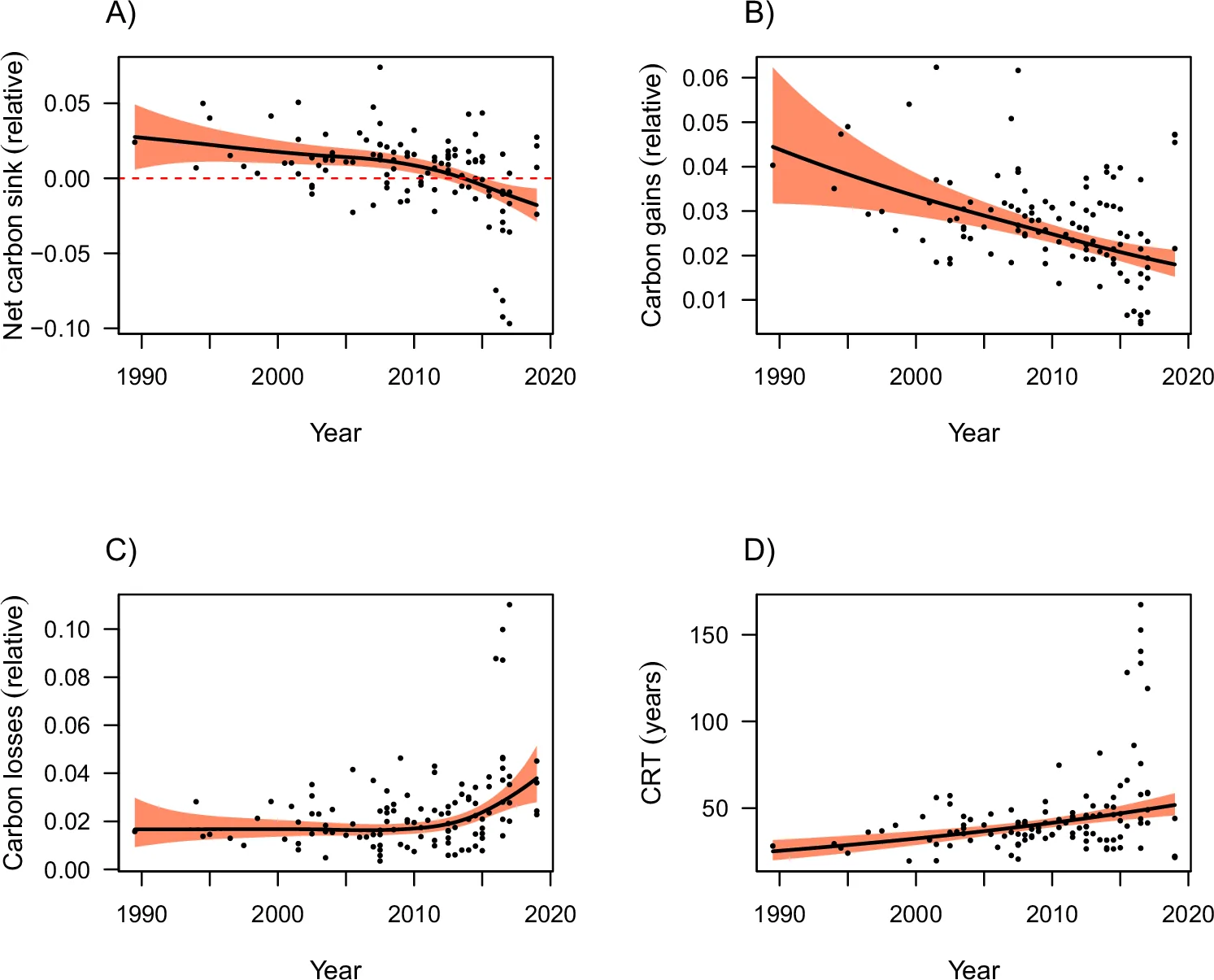
Long-term trends of the relative values (to above-ground carbon stocks) of above-ground carbon dynamics variables and above-ground carbon residence time (CRT). Curves are smooth splines fitted with generalized additive models (GAM). Points are partial residuals after accounting for the random effect of site (*n* = 49), corresponding to each census interval (*n* = 114). Shaded areas represent 95% confidence intervals. Statistical significance of the smooth terms was assessed using two-sided F-tests, with corresponding *p*-values reported in the legend and in Supplementary Table S1. **A** Net above-ground carbon sink (relative) (F(2.76, 3.47) = 11.04, *p* < 0.001), (**B**) above-ground carbon gains (relative) (F(1.39, 1.66) = 13.42, *p* < 0.001), (**C**) above-ground carbon losses (relative) (F(3.42, 4.29) = 7.17, *p* < 0.001), and (**D**) above-ground carbon residence time (CRT) (F(1.00, 1.00) = 19.95, *p* < 0.001). Source data are provided as a Source Data file.

Our results showed that tropical and subtropical forests of south and southeast Brazil experienced climatic fluctuations throughout the study period (1987-2020). Overall, mean annual precipitation (MAP) F(7.15, 8.13) = 40.23, *p *< 0.001, and climatic water deficit (CWD) F(6.9, 7.9) = 10.79, *p* < 0.001 displayed a non-linear trend, while mean annual temperature (MAT) F(7.12, 8.11) = 50.65, *p* < 0.001 increased and drought peaks were observed in 2003 and 2015 (Fig. S25). Above-ground carbon stocks across the sites generally increased until ~2013, when they began to decline (Table 1, Table S1, Fig. 2a) F(5.27, 6.37) = 22.79, *p* < 0.001. The net carbon sink declined over time and reached zero in ~2013 F(2.88, 3.63) = 9.05, *p* < 0.001, driven by increasing above-ground carbon losses F(2.56, 3.21) = 8.76, *p* < 0.001 and decreasing above-ground carbon gains F(2.25, 2.8) = 5.36, *p *= 0.004 (Fig. 2b–d, Fig. S4). Meanwhile, the data showed an increase in carbon residence time (CRT) F(1, 1) = 19.95, *p* < 0.001 (Table 1, Fig. 3d).

**Table 1:** Population-level predictions (average between sites at a given point in time) from the GAMs (Figs. 2 and 3) for 2010 and 2020

	2010	2020
Above-ground carbon stocks (Mg C ha^−1^)	98.89	91.408
Net above-ground carbon sink (Mg C ha^−1^ yr^−1^)	0.788	-2.059
Net above-ground carbon sink (relative)	0.009	-0.021
Above-ground carbon gains (Mg C ha^−1^ yr^−1^)	2.427	1.643
Above-ground carbon gains (relative)	0.025	0.017
Above-ground carbon losses (Mg C ha^−1^ yr^−1^)	1.65	3.362
Above-ground carbons losses (relative)	0.017	0.043
Above-ground carbon residence time (years)	41.56	53.14

Relative values are relative to the above-ground carbon stock of the site in the first year of the census interval. Source data are provided as a Source Data file.

**Fig. 4:**
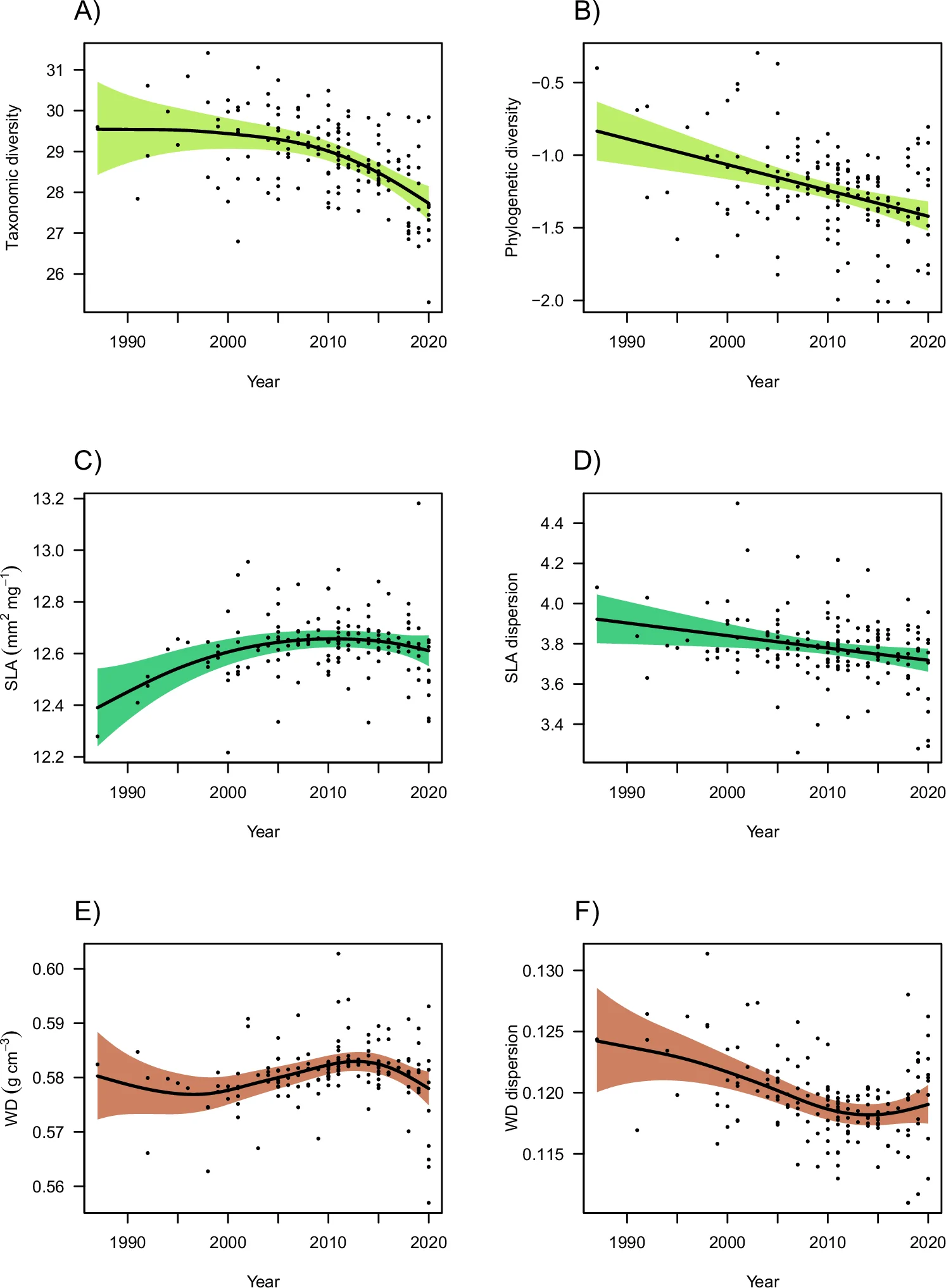
Long-term trends of taxonomic and phylogenetic diversity, functional trait community weighted means and functional trait weighted coefficient of variation (dispersion). Curves are smooth splines fitted with generalized additive models (GAM). Points are partial residuals after accounting for the random effect of site (*n* = 49), corresponding to each census (*n* = 163). Shaded areas represent 95% confidence intervals. Statistical significance of the smooth terms was assessed using two-sided F-tests, with corresponding *p*-values reported in the legend and in Supplementary Table S1. **A** Taxonomic diversity (Shannon) (F(2.64, 3.33) = 11.50, *p* < 0.001), (**B**) Phylogenetic diversity (PD) (F(1.00, 1.00) = 17.59, *p* < 0.001), (**C**) SLA (specific leaf area community-weighted mean) (F(2.77, 3.49) = 3.67, *p* = 0.012), (**D**) SLA dispersion (specific leaf area dispersion) (F(1.00, 1.00) = 6.31, *p *= 0.013), (**E**) WD (wood density community-weighted mean) (F(3.83, 4.76) = 2.91, *p* = 0.019), and (**F**) WD dispersion (wood density dispersion) (F(2.97, 3.73) = 5.75, *p* < 0.001). Source data are provided as a Source Data file.

Although taxonomic diversity remained stable before starting to decline in ~2005–2010, we observed a long-term decline in phylogenetic diversity (Table S1, Fig. 4A, B) (Taxonomic diversity (Shannon): F(2.64, 3.33) = 11.5, *p* < 0.001; Phylogenetic diversity (PD): F(1.00, 1.00) = 17.59, *p* < 0.001). Similarly, although specific leaf area (SLA) showed a positive trend until ~2010, after which it stabilised, SLA dispersion decreased linearly over time (Table S1, Fig. 4C, D) (SLA: F(2.77, 3.49) = 3.67, *p* = 0.012; SLA dispersion: F(1.00, 1.00) = 6.31, *p *= 0.013). Wood density (WD) was mostly stable (and even slightly increased) until about 2012, when it started to decline (Table S1, Fig. 4E) (WD: F(3.83, 4.76) = 2.91, *p* = 0.019). At the same time, WD dispersion, which showed a negative trend until about 2012, started to increase (Table S1, Fig. 4F) (WD dispersion: F(2.97, 3.73) = 5.75, *p* < 0.001).

### Above-ground carbon and climate relationships over time

**Fig. 5:**
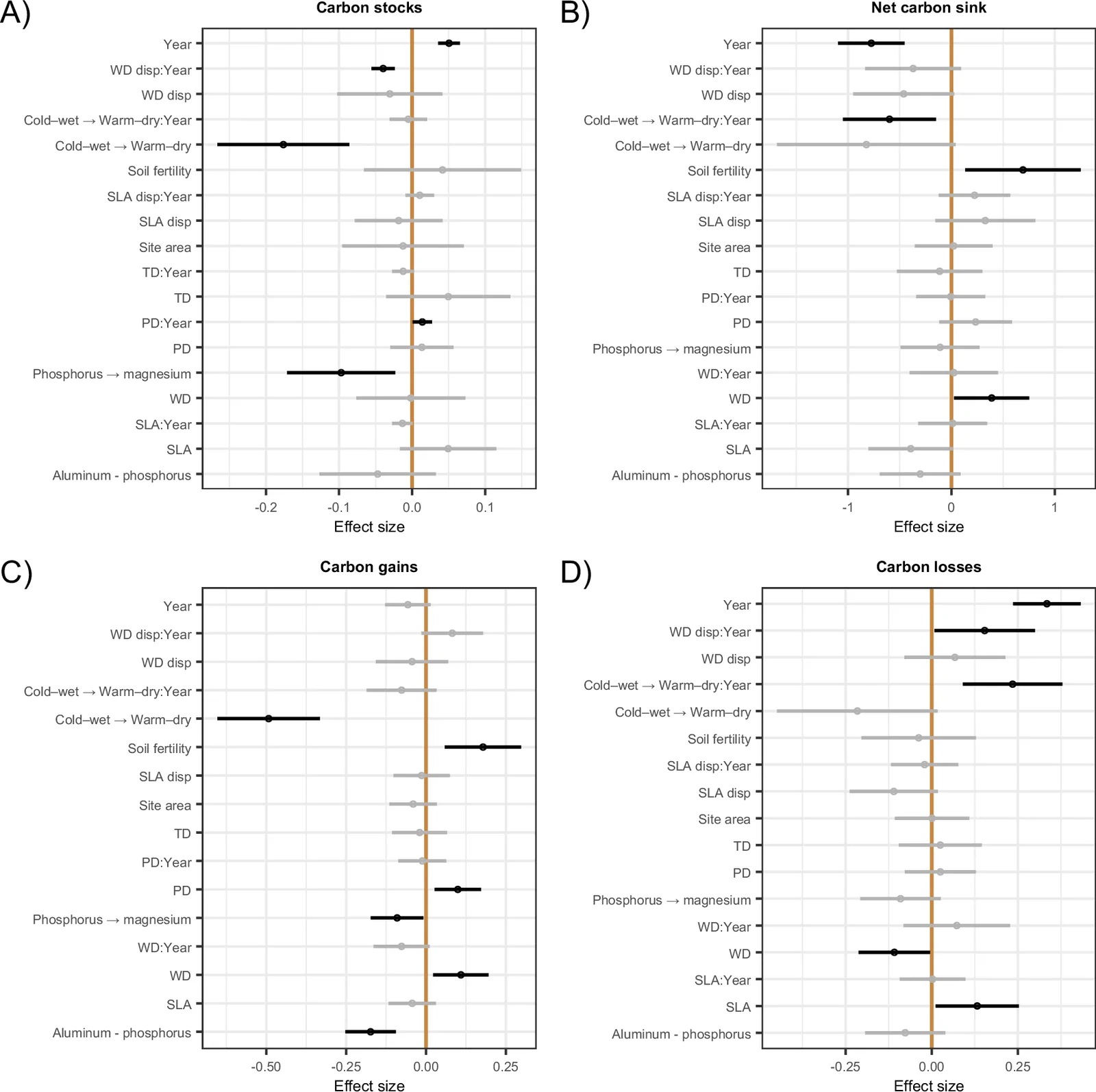
Conditional average coefficients for the selected models (∆AICc ≤ 4) for above-ground carbon stock, net above-ground carbon sink, above-ground carbon gains and above-ground carbon losses. All models included the effects of time, climate, soil, diversity and functional trait composition. The statistical significance was assessed using two-sided asymptotic z-tests. **A** Above-ground carbon stocks (marginal R² = 30.64%), (**B**) net above-ground carbon sink (marginal R² = 33.1%), (**C**) above-ground carbon gains (marginal R² = 47.1%), and (**D**) above-ground carbon losses (marginal R² = 28.6%). For each variable in the model, dots represent conditional averaged coefficients with their 95% confidence intervals; the coefficients and confidence intervals of variables not included in the averaged model were set to zero. The number of observations was 163 for above-ground carbon stocks and 114 for the other response variables, with data nested within sites (*n* = 49). Coefficients were estimated using linear mixed-effects models (LMM, including a random effect of site). Detailed results for each predictor (Estimate, Std. Error, Adjusted SE, z value, and Pr(>|z | )) are provided in Supplementary Tables S2–S9. WD, wood density community-weighted mean; WD disp, wood density dispersion; SLA, specific leaf area community-weighted mean; SLA disp, specific leaf area dispersion; PD, phylogenetic diversity; TD, taxonomic diversity. Source data are provided as a Source Data file.

**Fig. 6:**
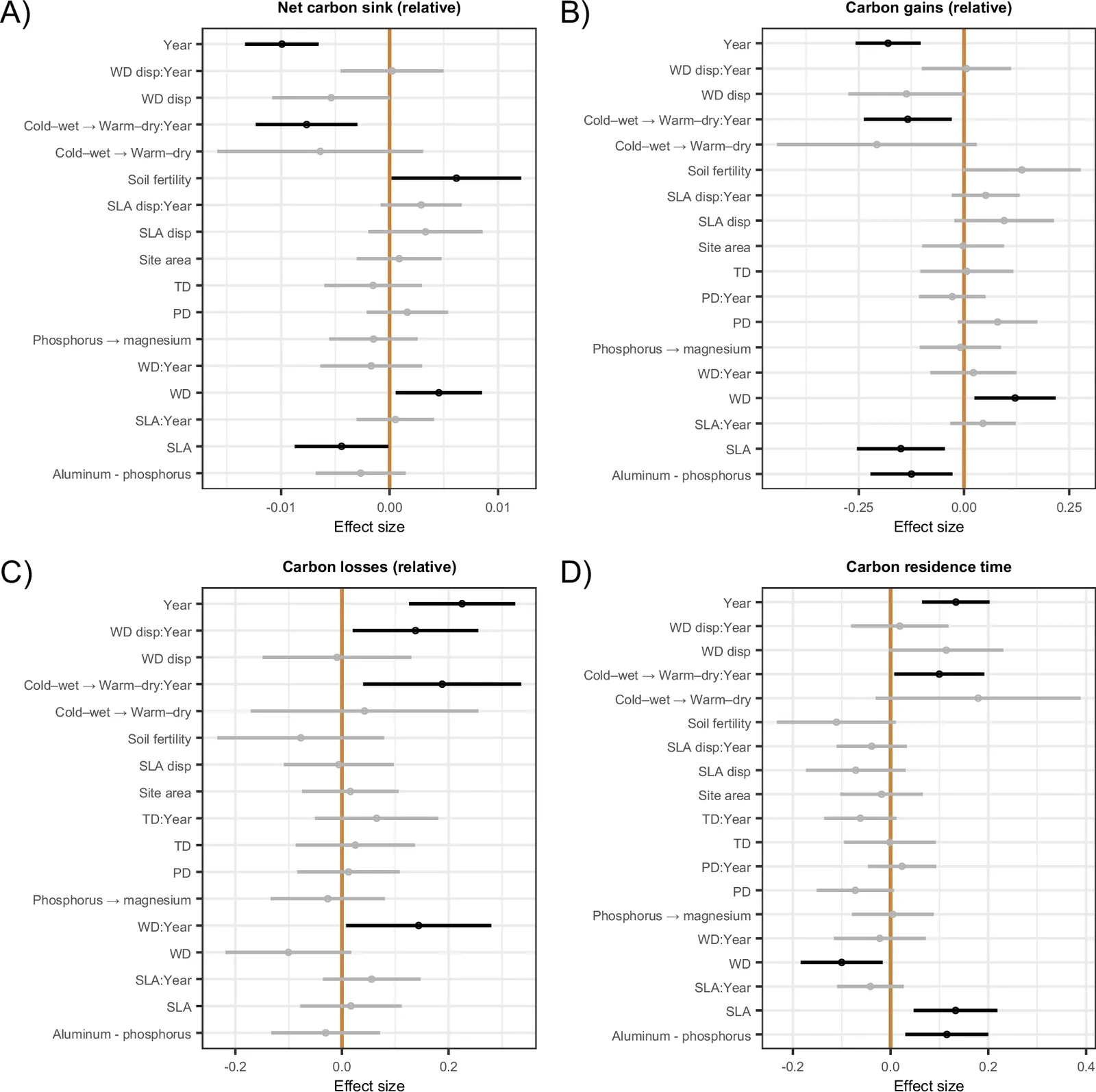
Conditional average coefficients for the selected models (∆AICc ≤ 4) for relative net above-ground carbon sink, relative above-ground carbon gains, relative above-ground carbon losses and above-ground carbon residence time. All models included the effects of time, climate, soil, diversity and functional traits composition. The statistical significance was assessed using two-sided asymptotic z-tests. **A** Relative net above-ground carbon sink (marginal R² = 34.7%), (**B**) relative above-ground carbon gains (marginal R² = 39.2%), (**C**) relative above-ground carbon losses (marginal R² = 18.3%), and (**D**) above-ground carbon residence time (marginal R² = 33.6%). For each variable in the model dots represent the conditional averaged coefficients with their 95% confidence intervals; the coefficients and confidence intervals of variables not included in the averaged model were set to zero. The number of observations was 114, with data nested within sites (*n* = 49). Coefficients were estimated using linear mixed-effects models (LMM, including a random effect of site). Detailed results for each predictor (Estimate, Std. Error, Adjusted SE, z value, and Pr(>|z | )) are provided in Supplementary Tables S2–S9. WD, wood density community weighted mean; WD disp, wood density dispersion; SLA, specific leaf area community weighted mean; SLA disp, specific leaf area dispersion; PD, phylogenetic diversity; TD, taxonomic diversity. Source data are provided as a Source Data file.

The direction of the relationship between each carbon variable and time varied. Above-ground carbon stocks (z = 6.61, *p *< 0.001, effect size = 0.051, 95% CI = [0.036, 0.065]), carbon losses (z = 6.66, *p* < 0.001, effect size = 0.334, 95% CI = [0.237, 0.431]), relative carbon losses (z = 4.44, *p *< 0.001, effect size = 0.225, 95% CI = [0.127, 0.324]) and carbon residence time (CRT; z = 3.80, *p* < 0.001, effect size = 0.134, 95% CI = [0.065, 0.202]) had a positive relationship with time, whereas net carbon sink (z = 4.73, *p* < 0.001, effect size = −0.775, 95% CI = [−1.092, −0.457]), relative net carbon sink (z = 5.74, *p* < 0.001, effect size = −0.010, 95% CI = [−0.013, −0.007]) and relative carbon gains (z = 4.58, *p* < 0.001, effect size = −0.180, 95% CI = [−0.257, −0.104]) had a negative relationship with time (Figs. 5–6, Figs. S26 and S27). The interaction between time and climate revealed that the warmest–driest sites had the steepest decline in net carbon sink (z = 2.60, *p* = 0.009, effect size = −0.599, 95% CI = [−1.044, −0.153]), due to the increasing above-ground carbon losses over time (z = 3.19, *p* = 0.001, effect size = 0.235, 95% CI = [0.092, 0.378]; Figs. 5 and 6, Figs. S26 and S27). The increases in CRT were more pronounced in the warmest–driest sites (z = 2.12, *p* = 0.034, effect size = 0.100, 95% CI = [0.009, 0.191]), while the CRT of the coldest–wettest sites showed a stable trend (Fig. 6, S27D).

In general, warm-dry sites had lower above-ground carbon stocks (z = 3.83, *p* < 0.001, effect size = −0.176, 95% CI = [−0.266, −0.087]) and carbon gains (z = 6.02, *p *< 0.001, effect size = −0.493, 95% CI = [−0.651, −0.334]) than cold-wet sites, although they showed similar values for net carbon sink, carbon losses and all relativised variables (Figs. 5 and 6, Figs. S26 and S27). Because of the interaction between climate and time, we found that the warmest–driest sites trended in recent years towards a lower net carbon sink (z = 2.60, *p* = 0.009, effect size = −0.599, 95% CI = [−1.044, −0.153]) and relative net carbon sink (z = 3.20, *p* = 0.001, effect size = −0.008, 95% CI = [−0.012, −0.003]), which were associated with lower relative carbon gains (z = 2.51, *p* = 0.012, effect size = −0.134, 95% CI = [−0.237, −0.030]) and higher carbon losses (z = 3.19, *p* = 0.001, effect size = 0.235, 95% CI = [0.092, 0.378]), relative carbon losses (z = 2.49, *p* = 0.013, effect size = 0.188, 95% CI = [0.041, 0.335]) and CRT (z = 2.12, *p* = 0.034, effect size = 0.1, 95% CI = [0.009, 0.191]) (Figs. 5 and 6, Figs. S26 and S27). Thus, the relationships between climate and above-ground carbon dynamics became more pronounced over time because of the decreasing net carbon sink of the warmest–driest sites (Figs. 5 and 6, Figs. S26 and S27).

### Above-ground carbon and soil relationships

We found a negative relationship between the phosphorus-to-magnesium PCA axis and above-ground carbon stocks and gains (Figs. 5 and 6; carbon stocks: z = 2.57, *p* = 0.010, estimate = −0.097, 95% CI [ − 0.171, −0.024]; carbon gains: z = 2.14, *p* = 0.032, estimate = −0.090, 95% CI [ − 0.172, −0.009]). This means that the phosphorus-rich and magnesium-poor forests seemed to have large and increasing carbon stocks. Soil fertility had a positive relationship with net carbon sink, relative net carbon sink, carbon gains and relative carbon gains, although the latter had *p* = 0.054 (net carbon sink: z = 2.43, *p* = 0.015, estimate = 0.692, 95% CI [0.139, 1.245]; relative net carbon sink: z = 2.02, *p* = 0.043, estimate = 0.006, 95% CI [0.000, 0.012]; carbon gains: z = 2.92, *p* = 0.003, estimate = 0.178, 95% CI [0.060, 0.297]; relative carbon gains: z = 1.93, *p* = 0.054, estimate = 0.138, 95% CI [ − 0.001, 0.276]). The aluminium–phosphorus PCA axis, which was positively related to both aluminium and phosphorus contents, had a negative relationship with both absolute and relative carbon gains and a positive relationship with CRT (carbon gains: z = 4.29, *p* < 0.001, estimate = −0.173, 95% CI [ − 0.252, −0.095]; relative carbon gains: z = 2.51, *p* = 0.012, estimate = −0.125, 95% CI [ − 0.221, −0.028]; CRT: z = 2.66, *p* = 0.008, estimate = 0.115, 95% CI [0.031, 0.199]).

### Above-ground carbon and ecological variables relationships

**Fig. 7:**
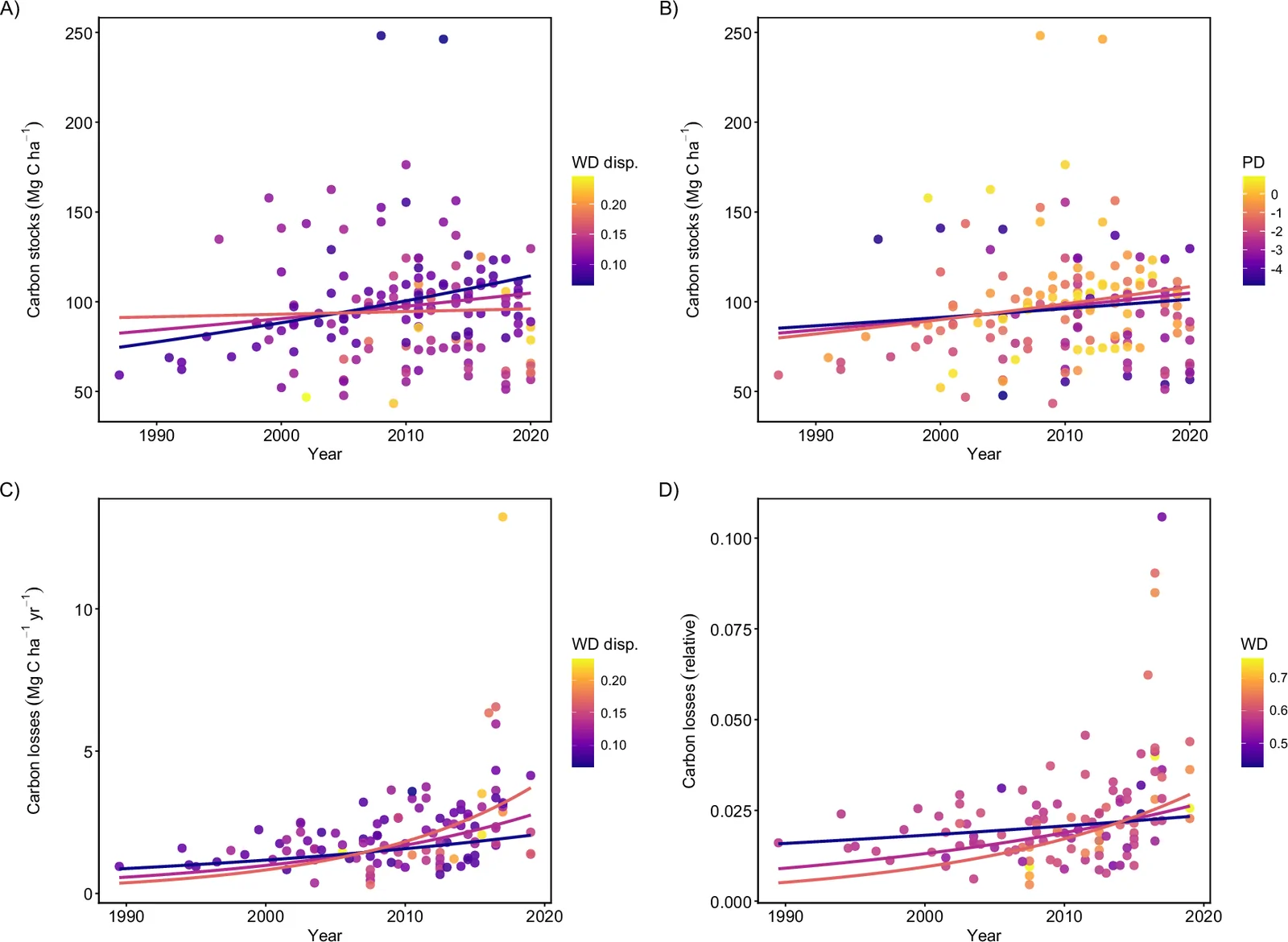
Temporal trends in carbon stocks and carbon dynamics across gradients of ecological variables. **A** Above-ground carbon stocks as a function of time (year) at varying levels of WD disp (WD dispersion; year: z = 6.61, *p* < 0.001, effect size = 0.051, 95% CI = [0.036, 0.065]; year × WD disp: z = 4.88, p < 0.001, effect size = −0.040, 95% CI = [−0.055, −0.024]); (**B**) above-ground carbon stocks as a function of time (year) at varying levels of phylogenetic diversity (PD; year × PD: z = 2.06, *p* = 0.039, effect size = 0.014, 95% CI = [0.001, 0.027]); (**C**) above-ground carbon losses as a function of time (year) at varying levels of WD disp (year: z = 6.66, *p* < 0.001, effect size = 0.33, 95% CI = [0.24, 0.43]; year × WD disp: z = 2.06, *p *= 0.039, effect size = 0.15, 95% CI = [0.01, 0.30]); and (**D**) relative above-ground carbon losses as a function of time (year) at varying levels of WD (WD community-weighted mean; year: z = 4.44, *p* < 0.001, effect size = 0.23, 95% CI = [0.13, 0.32]; year × WD: z = 2.07, *p* = 0.038, effect size = 0.14, 95% CI = [0.01, 0.28]). Points are raw residuals and the curves were fitted with linear mixed-effects models (LMM, including a random effect of site; same models described in Figs. 5 and 6). Results are based on conditional averaged coefficients from the selected models (∆AICc ≤ 4) and statistical significance was assessed using two-sided asymptotic z-tests. The number of observations was 163 for above-ground carbon stocks and 114 for the other response variables. Colours indicate the gradient of the interacting variables, ranging from dark purple (low values) to magenta and orange to bright yellow (high values). Source data are provided as a Source Data file.

We analysed the relationships between above-ground carbon and ecological variables with and without interactions with year. Excluding interactions, WD was positively linked to absolute net carbon sink (z = 2.10, *p* = 0.036, effect size = 0.39, 95% CI = [0.03, 0.75]), relative net carbon sink (z = 2.24, *p* = 0.025, effect size = 0.005, 95% CI = [0.001, 0.008]), absolute carbon gains (z = 2.47, *p* = 0.014, effect size = 0.11, 95% CI = [0.02, 0.20]) and relative carbon gains (z = 2.47, *p* = 0.014, effect size = 0.12, 95% CI = [0.03, 0.22]), and negatively linked to absolute carbon losses (z = 2.05, *p* = 0.040, effect size = −0.11, 95% CI = [−0.21, −0.01]) and CRT (z = 2.34, *p* = 0.019, effect size = −0.10, 95% CI = [−0.18, −0.02]). SLA was positively linked to absolute carbon losses (z = 2.14, p = 0.032, effect size = 0.13, 95% CI = [0.01, 0.25]) and CRT (z = 3.04, *p* = 0.002, effect size = 0.13, 95% CI = [0.05, 0.22]) and negatively linked to relative net carbon sink (z = 2.01, *p* = 0.045, effect size = −0.004, 95% CI = [−0.009, −0.000]) and relative carbon gains (z = 2.82, *p* = 0.005, effect size = −0.15, 95% CI = [−0.25, −0.05]). Finally, phylogenetic diversity (PD) was only positively linked to absolute carbon gains (z = 2.69, *p* = 0.007, effect size = 0.10, 95% CI = [0.03, 0.17]) (Figs. 5 and 6). Including interactions, forests with high WD dispersion showed a stable (rather than an increasing) carbon stock trend (z = 4.88, *p* < 0.001, effect size = −0.040, 95% CI = [−0.055, −0.024]) and high rates of both absolute carbon loss (z = 2.06, *p* = 0.039, effect size = 0.15, 95% CI = [0.01, 0.30]) and relative carbon loss (z = 2.30, *p* = 0.022, effect size = 0.14, 95% CI = [0.02, 0.25]). In contrast, forests with low WD dispersion showed higher carbon stocks in recent years and lower carbon losses (Figs. 5–7). We also detected a modest interaction between PD and year on carbon stocks (z = 2.06, *p *= 0.039, effect size = 0.014, 95% CI = [0.001, 0.027]), suggesting that forests with higher PD may have experienced a greater rate of carbon stock increase over time compared to those with lower PD (Fig. 7).

## Discussion

### Long-term trends of carbon dynamics and diversity

Forests with negative net above-ground carbon sinks (i.e., carbon sources) are widespread across the entire sampled climate space. Although the coldest-wettest sites had higher above-ground carbon uptake and stocks *per* year when compared with the driest-warmest sites, most are transitioning from net carbon sinks to net carbon sources over time^2^. We also found that both diversity and mass-ratio mechanisms may simultaneously drive above-ground carbon dynamics, with context-dependent effects.

We found a long-term decline in taxonomic, functional, and phylogenetic diversity (Fig. 4). These results indicate functional homogenisation of Brazilian tropical and subtropical forests driven by climate change^23,28^. Functionally, these forests have shifted during most of the monitoring period towards a narrower range of specific leaf area (SLA) and wood density (WD). Although the direct drivers of this homogenisation are difficult to determine and we have not delved into these aspects, recent literature suggests that drought and rising temperatures are likely to be the most important drivers^23,28,39^. Although disturbance and forest age are expected to be spatially homogeneous in our samples based on the sampling criteria and historical data, successional processes cannot be completely disregarded. However, assuming that successional processes are controlled in our samples, diversity and carbon losses can be a result of physiologically driven tree mortality^28^. In fact, with more trees dying because of physiological responses to climate fluctuations, certain species, traits, and lineages, particularly the ones with narrower niches, are more likely to disappear from a given community^40^. Conversely, rare species inhabiting non-optimal environments might benefit from climate change if the emerging conditions align more closely with their niche requirements.

Both functional trait variables assessed (SLA and WD) showed subtle fluctuations over time. The WD trend changed from slight positive to negative in ~2012, coinciding with the change in the WD dispersion trend, which started to increase around the same time. These shifts also coincided with the period when the forests shifted from carbon sinks to carbon sources. It is possible that the above-ground carbon sink decline was associated with the death of large individuals from high-WD species, which decreased the average WD of the communities. Because high-WD species are less prone to cavitation and usually perform better under water-limited conditions, one could expect an increasing WD trend in forests experiencing climate change effects^12,28^. However, our results contradict this expectation by showing that, temporally, the carbon sink decline seemed associated with the death of individuals from conservative high-WD species. Moreover, although SLA was more stable than WD after 2012, it showed a slight decrease thereafter, while SLA dispersion showed an almost linear decrease throughout the monitoring period.

Overall, these results may suggest two paths in the functional composition trends of our sites. First, a growing presence of species with thicker leaves and lighter wood in the forest community could indicate an increasingly conservative strategy at the leaf-trait level and an increasingly acquisitive strategy at the wood-trait level. Alternatively, if such trends are no more than oscillations, both wood and leaf traits could remain stable around the values they have reached in the last decades (Fig. 4). However, carbon losses were associated with lower WD and higher SLA, indicating conservative strategies over time (i.e., no interactions between time and the community-weighted means of WD and SLA). This relationship was not affected by the shift that occurred around 2012. Although our results do not reveal the direct drivers of the diversity decline, these trends suggest that even in the absence of deforestation, species and lineage losses are likely to occur at a substantial rate due to climate change^39^. Strong climate change effects are expected to be seen in the Amazon in the next years^39^; however, in forests that already have lower diversity and biomass and are under more limiting environmental conditions, strong climate change effects may have already started^2,37^.

The difference between the net above-ground carbon sink of the warmest-driest sites and that of the coldest-wettest sites has increased in the last few decades^2^. Due to greater above-ground carbon losses over time, the net carbon sink of the driest forests was smaller than that of the wettest forests^2^. Therefore, the above-ground carbon stock differences between the driest and the wettest sites have increased, with the wettest forests maintaining the highest carbon stocks. In contrast with Maia et al. 2020^2^, the relationship between time (and its interaction with climate) and carbon gains was not statistically significant in our full model, revealing that the carbon sink decline is more likely to be caused by the increasing carbon losses. However, the relative carbon gains declined over time, with the most pronounced decline observed among the warmest-driest sites. This result indicates increasing tree growth limitation in the warmest-driest sites, further reinforcing their vulnerability to climate change. The CRT of the warmest-driest sites has experienced a long-term increase and is currently greater than that of the coldest-wettest sites. The decreasing carbon gains of the warmest-driest sites are likely driving the CRT increases because CRT = carbon stocks / carbon gains. These patterns were also found in Maia et al. (2020)^2^ (except for relative values and CRT, which were not included in the 2020 study), which used 32 sites of the 49 used here. Expanding the latitudinal and longitudinal gradients of the previous study did not change these results, indicating that the subtropical forests of southern Brazil and the semideciduous forests of central Brazil are probably following a similar trend.

### Environmental and ecological drivers of above-ground carbon dynamics

Both the phosphorus-to-magnesium and aluminium-phosphorus axes of the soil PCA had negative relationships with carbon gains. In other words, carbon gains were generally lower in aluminium-rich and magnesium-poor forests, although the relationship with phosphorus showed conflicting trends depending on the soil fertility axis considered. Here, we interpret these results considering that carbon gains are likely to decrease with aluminium and increase with phosphorus for the following reasons: (i) the contribution of phosphorus in the aluminium-phosphorus component is smaller than that of aluminium, (ii) the contribution of phosphorus in the phosphorus-to-magnesium component is greater than that of magnesium, and (iii) phosphorus-to-magnesium had a negative relationship with carbon stocks. These findings are consistent with the negative effects of aluminium toxicity and the positive effects of soil phosphorus availability on tree growth^41–44^. For relative carbon gains, only a relationship with aluminium-phosphorus was detected, further indicating the detrimental effect of aluminium toxicity for tree growth in these forests. The positive relationship between aluminium-phosphorus and CRT probably owes to the relationship between CRT and carbon gains (CRT = carbon stocks/carbon gains). The potential negative relationship between magnesium and carbon stocks and gains should be further investigated. However, as the contribution of magnesium to the phosphorus-to-magnesium axis was smaller than the contribution of phosphorus, these relationships are probably more associated with phosphorus than magnesium. Moreover, we also found that the net carbon sink, relative net carbon sink and carbon gains increased with soil fertility, as expected considering that low nutrient availability limits plant growth^45,46^. This finding highlights the confounding effects of soil and climate on this region’s carbon dynamics, because the warmest-driest sites (deciduous forests) contain the most fertile soils due to slower nutrient leaching^47^. Therefore, as expected, deciduous forests’ lower above-ground carbon stock and sink are probably related to climate.

Carbon gains increased with phylogenetic diversity (Fig. 5C) and the temporal increases in carbon stocks were slightly more pronounced in sites with higher phylogenetic diversity than in sites with lower phylogenetic diversity (Figs. 5A and 7B). Since the phylogenetic relationships among species tend to predict functional similarity to a certain extent^8,23^, the effect of PD on carbon gains is consistent with niche complementarity effects and recent findings on carbon-diversity relations in tropical forests^6^. Therefore, because we already accounted for SLA dispersion and WD dispersion, niche complementarity could be related to other factors, as follows. (i) The portions of SLA and WD not captured by our data, (ii) unmeasured traits involved in carbon processing and storage (e.g., root, stem and leaf physiology and canopy architecture^8,23^) and/or (iii) the lower vulnerability of distantly related species to similar pathogens due to pathogen dilution effects^48^.

Forests with higher WD dispersion showed the most pronounced increases in both absolute and relative carbon losses, while their carbon stocks remained relatively stable over time – unlike the general trend of increasing carbon stocks (Figs. 5–7). These relationships likely reflect the negative relationships between WD dispersion and net carbon sink and relative net carbon sink, which had small, although non-statistically significant, *P*-values (*P* = 0.07; Figs. 5–6). This indicates that trees with WD values approaching the community’s extremes are being eliminated, given the decreasing tendency of WD dispersion (Fig. 4F). On the other hand, the carbon losses suffered by forest communities with a narrower WD range was mostly stable over time, suggesting resilience to climatic fluctuations. At the same time, WD was positively linked to net carbon sink and carbon gains (and their relativised values) and negatively linked to carbon losses and relative carbon losses, although the latter was not statistically significant (*P* < 0.1). Meanwhile, WD had a negative effect on CRT, which can be related to its positive effect on carbon gains. Although one could argue that the positive relationship between WD and carbon gains and net carbon sink is biased by the use of WD in the calculation of above-ground biomass - a precursor for the estimation of above-ground carbon -, this is contradicted by our results. First, above-ground carbon stocks showed no relationship with WD. Second, high WD seemed consistently linked to tree survival and growth throughout the monitoring period. Third, the relative values of carbon gains and net carbon sink (expected to be unbiased by community functional composition) also showed a positive relationship with WD. Finally, carbon losses were negatively associated with WD. These results indicate that the observed relationships are unlikely to be an artefact of WD-based calculations. However, we have also observed an increasing trend of relative carbon losses over time, regardless of community WD. Since the relationships between WD and carbons gains and between WD and net carbon sink have remained stable over time, this suggests that the typical pattern of high WD predicting a high above-ground carbon sink may shift in the future.

The relationship between WD and SLA with above-ground carbon variables suggests that forests with a predominance of softer leaves and lighter wood have been the most vulnerable. High-SLA forests (i.e., with softer leaves) had the greatest carbon losses and the lowest relative carbon sinks and relative carbon gains in our dataset. This pattern is not likely to have changed over time since no statistically significant interactions between SLA and time were detected. These findings are consistent with the hypothesis that, despite faster turnover among acquisitive species, acquisitive traits predict higher vulnerability under limiting environmental conditions, such as during drought events^12,26^. For example, high SLA is typically linked to high respiration and transpiration rates, which may impair growth and survival during drought periods^12,26,31^. Conversely, low SLA (i.e., thicker leaves) enables plant functioning to persist even at reduced leaf water potentials during dry periods^12^. Therefore, our findings reinforce that, under limiting conditions, conservative traits are more associated with growth and survival than acquisitive traits^12,26,28^. Thus, although acquisitive traits are typically linked to higher growth rates and faster turnover, they are also linked to higher mortality, competition, vulnerability to drought-induced cavitation and temperature-induced water loss^12,26,33,34^. At the same time, SLA was positively linked to CRT, indicating that above-ground carbon tends to persist longer in forests with higher SLA (i.e., softer leaves). Combined with the observation of higher CRT in low-WD forests, this indicates that forests characterised by acquisitive SLA and WD composition may retain carbon for longer periods. However, given the long-term increase in carbon stocks followed by a recent decline over the last 10 years, these patterns may shift as carbon stocks stabilise in the future^12,28^.

### Final remarks

Our results showed that Brazilian tropical seasonal forests and subtropical evergreen forests have experienced a decline in tree diversity and above-ground carbon sink. The relationships between above-ground carbon dynamics and diversity seem to be context-dependent: while phylogenetic diversity predicted higher carbon gains, trait diversity (particularly wood density) predicted higher carbon losses over time. We also found that forests composed of mostly conservative species (i.e., denser wood and thicker leaves) tend to show higher productivity. We recognise the limitations of the data, especially related to the phylogenetic and functional variables and the application of complex (and less generalisable) models for data exploration and hypothesis testing. We also acknowledge the need for precise measures of disturbance history and forest age to better understand the above-ground carbon sink dynamics in these regions. However, our sampling strategy was designed to represent the most conserved and least disturbed forests, so disturbance and age are unlikely to confound the observed relationships. This supports the reliability of our findings, although the role of disturbance in these forests warrants further investigation. In addition, as our samples are spatially clustered, caution is needed when extrapolating our conclusions to the regions between the large-scale clusters.

Our evidence suggests that conservation policies that do not take carbon-diversity relationships into account may be less effective. Therefore, to jointly safeguard different facets of forest diversity and preserve forest carbon sinks, carbon-diversity relationships should be considered. These are also useful for restoration planning and for predicting the vulnerability and resilience of these forests in the near future. In addition, these results have relevant implications for the conservation of ecosystem services, which can be extended to other forests in south and southeast Brazil. Our target sites have been protected from anthropogenic disturbances and monitored for decades, which sets them apart from most forests in the region, largely neglected by conservation efforts. Consequently, the concerning trends observed in above-ground carbon sink and biodiversity reduction in this study may be even worse in less conserved forests, leading to more severe consequences for ecosystem services.

## Method

### Study design

We used above-ground woody biomass data from 49 repeatedly measured forest sites in Brazil, characterized as deciduous, semideciduous, and evergreen forests (Supplementary Data 1; data of 32 sites were used in Maia et al. 2020^2^). Field measurements were conducted with permission from landowners when sites were located on private properties. For sites located within protected areas or lands administered by public institutions, access and research activities were carried out with authorization from the responsible authorities. In Brazil, this commonly includes agencies such as ICMBio (Chico Mendes Institute for Biodiversity Conservation) and other relevant environmental management bodies. These forest monitoring plots are part of long-term research efforts that have been maintained for decades, with repeated measurements conducted under the appropriate institutional permissions and in compliance with local, national, and international regulations governing research access and environmental monitoring. The sites are located in tropical and subtropical regions across south and southeast Brazil, spanning the latitudes 28° 19’ S to 14° 16’ S (Fig. 1). The mean annual temperature (MAT), mean annual precipitation (MAP) and mean altitude of the sites were, respectively, 20.6 °C (from 13.3 to 25.2 °C), 1450 mm (from 669 to 1998 mm) and 900 m a.s.l. (from 450 to 1536 m a.s.l.). Altitude is negatively correlated with MAT across the sites (*r* = -0.84). Both MAT and MAP follow a latitudinal gradient, with MAT increasing and MAP decreasing northward (Fig. S1). The driest and warmest forests in our gradient (tropical dry deciduous) are established on fertile soils in the northernmost sites, while the coldest and wettest forests in our gradient (subtropical and tropical evergreen) are established on soils of varying fertility but generally poorer in the southernmost sites. The semideciduous forests experience an intermediate climate in the tropical region. The minimum distance between sites is ~1 km and the maximum distance is >1000 km.

The 49 forest sites are distributed across four spatial clusters (Fig. 1), which are strongly correlated with climate. Despite substantial within-cluster climate variation, we achieved 100% accuracy when using climatic variables to predict the cluster assignment of each site. Sites within the same cluster have an average maximum distance of ~150 km and an average distance of ~60 km. Such distribution characterises large-scale variations (between clusters) and regional variations (within clusters). Despite the spatial proximity between some of the sites (<5 km), there is high local-scale environmental heterogeneity within the clusters, driven by soil, topography and proximity to rivers and streams. Hence, we can assume independence between the sites, since their climatic and compositional similarities will have been incorporated by the model predictors (i.e., climate and functional composition).

The sites were selected to represent forests at advanced successional stages with minimal human intervention. Although we were unable to determine the exact age of each forest, we conservatively estimate that they are likely over 50 years old. Based on field observations and personal communication with reserve managers, local communities and landowners, the sites have not experienced the effects of fire, landslides, floods or logging during or before the monitoring period. Thus, disturbance history and forest age are not expected to be correlated with space and environmental conditions.

Site area ranged from 3.4 to 1179 ha (mean of 112 ha), sampled with multiple subplots of 400 m^2^ (in most sites). The area sampled in each site ranged from 0.2 to 5 ha (mean of 1.03 ha), totalling 50.4 ha across all sites. Although most subplots were located in forest interiors, edges were also captured, since sampling was designed to cover within-site heterogeneity in most cases. The number of censuses *per* site ranged from 2 to 8 (mean 3) and the total monitoring length, from 1 to 31 years (mean 11 years) (Fig.S1). The length of the census intervals, between 1 and 9 years (mean 5 years), varied among the sites and within the same site over time (Fig. S1). Forest census data is hosted in the ForestPlots.net platform (https://www.forestplots.net).

### Tree vegetation sampling

In each census, we measured all trees with a quadratic diameter at 1.3 m (qDBH) ≥ 5 cm. This approach enables the inclusion of multi-stemmed trees with slender stems (diameter at breast height <5 cm) if their quadratic diameter reaches 5 cm^49^, following the operation: qDBH = √(DBH_1_^2^ + DBH_2_^2^ + … + DBH_n_^2^); where DBH is the diameter at 1.3 m of each stem of a given tree. A different inclusion criterion was used in eight semideciduous forest sites, in which multi-stemmed trees were included if at least one of their stems had a diameter at breast height (DBH) ≥ 5 cm. The height of the point of measurement (POM) was marked in each stem and used as a reference for the ensuing censuses in most sites. See Fig. S2 and S3 for site-specific DBH and qDBH distributions. Taxonomic nomenclature was based on Angiosperm Phylogeny Group IV (APG IV 2016)^50^ and standardised using the R package flora^51^, following Flora do Brasil 2020^52^. We dropped Arecaceae, Dicksoniaceae, Cyatheaceae, and Cactaceae (~1% of the stems) to avoid biases in biomass calculation.

### Above-ground carbon stocks and dynamics

To calculate above-ground woody biomass (AGWB) from the census data, we extracted wood density values (WDi) from the Global Wood Density Database, the published literature, and field collections (details in the “Functional traits and functional diversity” section). We used the modified pantropical allometric equation of Chave et al. (2009)^32^ through the R package BIOMASS^53^. This equation has parameters for DBH, WDi and environmental stress (E), which is used to estimate tree height. We alternatively considered using a height-diameter allometric equation to estimate heights. However, we decided against this approach because the biomass estimated from this equation was more correlated with the climate variables than the biomass estimated from using the E parameter in the modified pantropical allometric equation. We converted the AGWB estimates to above-ground carbon (AGWC) by multiplying AGWB by 0.456^54^. Carbon stocks (Mg C ha^-1^) were estimated for each site in each census by summing the AGWC of each living tree and scaling the result to a per-hectare basis. To estimate above-ground carbon gains (Mg C ha^-1^ yr^-1^), we first summed the AGWC represented by the growth of each surviving tree (captured by changes in DBH) and the AGWC of newly recruited trees (those that reached 5 cm DBH or qDBH from one census to the next). Recruits were assumed to have DBH = 0 at the start of the interval^55^. Then, the above-ground carbon gains were divided by census interval length (in years) and scaled to a per-hectare basis. Above-ground carbon losses (Mg C ha^-1^ yr^-1^) were calculated as the sum of the AGWC of the trees and stems that died during the interval divided by the interval length and scaled to a per-hectare basis. We corrected the above-ground carbon gains and carbon losses using the equation CIC_1_ proposed by Talbot et al. (2014)^55^ to deal with the bias caused by census interval length. We applied this correction because larger census intervals can underestimate productivity, therefore, a small value, which is proportional to the interval length, is added to the observed values^55^. Net above-ground carbon sink (Mg C ha^-1^ yr^-1^) was calculated as carbon gains minus carbon losses. We also calculated the relative values (%) of above-ground carbon gains, carbon losses and net carbon sink by dividing each one by the carbon stocks of the start of the census interval. Carbon residence time (CRT, in years) was calculated as above-ground carbon stocks divided by carbon gains^1,14^. Census interval length was calculated as the difference between the end and start years of each interval, using decimal years to account for months. For the subsequent forest dynamics analyses (carbon gains, carbon losses, and net carbon sink), we used the midpoint year of each interval, calculated as the average of the end and start years and rounded to the nearest year. Carbon stock estimates were based on census-level data, while analyses of forest and carbon dynamics were based on census-interval data.

### Taxonomic diversity

We used the effective number of species (Hill numbers) to represent tree taxonomic diversity^56^. To account for uneven sampling efforts across the sites, we used the R package iNEXT^56,57^, applying coverage-based rarefaction and extrapolation assuming 90% of sample completeness. For each census at each site, we calculated rarefied/extrapolated Hill numbers for species richness (*q* = 0), the exponential of Shannon’s entropy index (*q* = 1), and the inverse of Simpson’s concentration index (*q* = 2). However, due to strong correlations among these metrics (*r* > 0.9; Fig. S5), we retained only Shannon diversity for further analyses. Hence, to represent taxonomic diversity during each census interval, we used the midpoint value of Shannon diversity, calculated as the mean of the start and end census values.

### Functional traits and functional diversity

We extracted most wood density values (WD, g cm^-3^) from the Global Wood Density Database^32,58^ and the published literature^59–63^. For 7.9% of the species and 16.3% of the basal area in our dataset, we used field-collected WD values. Similarly, specific leaf area (SLA, mm² mg^-1^) was obtained from the literature^12,59–65^ and field-collected data. The latter represented 27.5% of the species and 64.2% of the total basal area in our dataset. Species names in all data sources were standardised as per Flora do Brasil 2020^51,52^ to maximise matches with our dataset. The field collection and trait measurements followed the standard protocol proposed by Pérez-Harguindeguy et al. (2013)^66^. When trait values were not available for a given species in our dataset, we used the average trait value of other species in its genus or family. When the same trait was available at the species level in more than one source, we used the average of the available values. On average across all censuses, the individuals for which we used species-level WD and SLA represented 61.7% ± 17.7% and 76.7% ± 18.8% of the above-ground carbon stocks, respectively (mean ± SD). Genus-level data were used for 27.3% of the carbon stocks for WD and 16.14% for SLA, while family-level data accounted for 10.5% and 5.6%, respectively (Fig. S6 and S7). Although the practical difficulties of massive trait data collection in the field render the use of average traits from the literature a common approach, it entails a high level of uncertainty. Therefore, the trait variables used here can only be viewed as a rough representation of reality instead of a precise metric. From the trait data, we calculated the community-weighted means (CWM) of each trait for each site and census, using the individuals’ basal area as weights. Hereafter, the CWM of wood density will be referred to as WD and that of specific leaf area as SLA.

To represent the dispersion of each functional trait, we calculated their weighted standard deviation per census (referred to as “WD dispersion” and “SLA dispersion”). To account for differences in sample size, we applied a randomisation approach with 100 simulations per site and averaged the resulting CWMs. In each simulation, we randomly selected 100 tree individuals – the minimum number of trees recorded in a census – from each site to calculate a CWM. For census interval data (i.e., dynamics data) we used the CWMs and dispersions corresponding to the midpoint of the interval, consistent with our approach for other variables.

### Phylogenetic diversity

From the total of 942 morphospecies (12 identified to the genus level, 930 to the species level), we standardised all of those identified to the species level with The Plant List, using the package taxonstand^67^. Name standardisation aimed to maximize the number of matches between the species recorded in our dataset and the ALLOTB phylogeny, which contains over 350,000 tips^68^. This phylogeny is based on DNA sequences from GenBank and the Open Tree of Life as a backbone (more details in Smith and Brown 2018)^68^. Pruning this large phylogeny to match our dataset resulted in a tree containing ~88% of all species (~94% of the basal area recorded across the censuses). We added to the supplementary data files the list of which species were matched from the dataset to the phylogeny (Supplementary Data 2).

To summarise the evolutionary history of each tree community in each census, we used the R package picante^69^ to calculate phylogenetic diversity^70^. Phylogenetic diversity sensu stricto is the total sum of branch lengths of all taxa from a community comprised in a phylogeny. Because this metric is highly correlated with species richness^71^, we calculated its standardised effect size by comparing each observed value with that obtained under a null expectation. To do this, we randomly shuffled the tips of the phylogeny 999 times and, each time, calculated a phylogenetic diversity value for each randomised phylogeny. The standardised effect size was then computed by subtracting the mean of the randomised values from the observed value and dividing the result by their standard deviation^69^. We used this approach because it enables the detection of high or low evolutionary diversity regardless of species richness, which is sensitive to sampling effort.

### Climate data

We extracted multiple temperature, precipitation, and evapotranspiration variables from the Climatic Research Unit (CRU TS version 4.04; released 24 April 2020; https://crudata.uea.ac.uk/cru/data/hrg^72^): mean monthly temperature (°C), mean monthly daily maximum temperature (°C), monthly precipitation (mm) and daily potential evapotranspiration (dPET, mm) with a 0.5° resolution. We used 1 km² resolution temperature and precipitation data from WorldClim^73^ to downscale the CRU data (except dPET) via the delta spatial downscaling method^74^. From the downscaled data, we calculated the average values of mean annual temperature (MAT), maximum temperature and mean annual precipitation (MAP) across the full years within each census interval. Additionally, we calculated the annual maximum climatic water deficit (CWD) by summing the monthly differences between downscaled precipitation (mP) and potential evapotranspiration (PET), considering only months where PET exceeded mP (i.e., water deficit), following Chave et al. 2014^75^. For the first census of the census-level data (used only in the carbon stocks models), we used the mean climate values from the preceding 5 years. Due to their strong correlation (Fig. S8), we used principal component analysis (PCA) to synthesise the climatic variables. The first PCA axis explained 91% of the variance and described a cold-wet to warm-dry climatic gradient (low to high values, respectively). We used this axis in the analyses at both the census and census interval levels. Further details on the PCA are provided in the Statistical Analyses section.

### Soil data

In the first census of each site, soil surface samples (20 cm of depth) were collected from three points in each subplot and later combined into one composite sample. Following Empresa Brasileira de Pesquisa Agropecuária’s protocol^76^, the samples were sent for analysis of the following parameters: pH in water, phosphorus (mg cm^-3^) (P), potassium (mg cm^-3^) (K), calcium (cmol_c_ dm^-3^) (Ca), magnesium (cmol dm^-3^) (Mg), aluminium (cmolc dm^-3^) (Al) and sum of bases (SB) (sum of Ca, K and Mg; cmol_c_ dm^-3^). For each site, we used the mean value of each soil variable measured across its subplots. Due to strong correlation among the soil variables, we also used PCA to synthesise edaphic variation. The first three PCA axes, which explained 89% of the variance (63.8%, 12.7% and 12%, respectively), were retained for analysis. The first axis was positively associated with soil fertility – characterized by higher levels of pH, P, K, Ca, Mg and SB, and lower levels of Al. The second axis was positively associated with Al and P, while the third was positively associated with Mg and negatively with P. More details on the PCA are provided in the Statistical Analyses section.

### Controlling for edge effects

Edge effects on forest microclimatic conditions can affect above-ground carbon dynamics through changes in plant community composition, phenology, forest structure, and plant growth^77,78^. Since our sampling design aimed to maximise the area covered within each site, smaller sites tended to have greater exposure to edge effects. Hence, to control for the influence of edge effects in carbon dynamics, we included site area in the models. More details are provided in the Statistical Analyses section.

### Statistical analysis

We performed all statistical analyses in the R environment version 4.0.2^79^, using the following packages for graphics: ggplot2, GGally, grDevices, cowplot, viridis and corrplot^79–84^. To account for the non-independence of repeated observations within sites over time (e.g., multiple censuses and intervals per site), we included random intercepts for site in all models. This approach accounts for temporal autocorrelation and site-level variation by allowing each site to have its own baseline, thus controlling for within-site dependence inherent to long-term forest monitoring.

We used linear mixed-effects models (LMM) from the package lme4^85^ and generalised additive models (GAM) from mgcv^86^. Due to different spatial and temporal sampling efforts across sites, we weighted the observations by area sampled and census interval length (Fig. S1). For the forest and above-ground carbon dynamics models, we used the following weighting procedure, following Hubau et al. 2020^1^: interval length^1/3^ + sampled area^1/4^ – 1. The carbon stocks models were weighted by the cubic root of the sampled area^14^, while the temporal climatic trend models were weighted by the cubic root of interval length. To meet model assumptions (normality and homoscedasticity of residuals), the response variables were log-transformed when necessary, and site area was log-transformed to reduce the influence of extreme values. To facilitate comparisons among effect sizes, the predictors were centred to zero-mean and scaled to unit variance, allowing effect sizes to be interpreted as the change in the response variable associated with a one-standard-deviation increase in the predictor. Using the package spdep^87^, we tested for spatial autocorrelation in model residuals with Moran’s I. The results indicated no significant spatial autocorrelation, suggesting that spatial dependencies among observations did not bias model inferences.

Long-term carbon, vegetation, and climate trends were regressed as a function of time (year) using GAMs (Eq. 1). To maximise the use of available data, we used census-level instead of interval-level data – particularly for above-ground carbon stocks (also for LMMs models), taxonomic diversity, phylogenetic diversity, and trait variables. Since no model selection would be performed, we used Restricted Maximum Likelihood (REML) for the GAM model fits. We did not use GAMs in further analyses due to the relatively small sample size, which would not support the estimation of numerous parameters. In models with multiple predictors and interactions, detailed below, the large number of smooth splines would risk overfitting.

Other than the long-term carbon trends, we fitted LMMs to evaluate the relationships between the above-ground carbon variables and other predictors (Eq. 2). Hence, carbon stocks and carbon dynamics (in absolute and relative values) were modelled as a function of site area (i.e., proxy for edge effects), soil and interactions between time (year) and multiple ecological variables (i.e., taxonomic diversity, phylogenetic diversity, WD dispersion, SLA dispersion, WD CWM, and SLA CWM).

Climate and soil data were synthesised by principal component analysis (PCA). For each set of variables (either climate or soil), we centred and scaled all original variables and then submitted them to a PCA using the R packages FactoMineR^88^ and factoextra^89^. Climate was described by its first PCA axis, while soil was described by its first three PCA axes.

We tested the inclusion of spatial cluster as a predictor in the models. However, the strong association between cluster and climate due to the broad latitudinal gradient captured by the clusters resulted in extremely high variance inflation factor (VIF) in models where cluster and climate were both included as predictors. Hence, we opted for the inclusion of climate instead of spatial cluster for two main reasons: first, for being a tangible, continuous variable instead of an arbitrary, discrete variable; second, for capturing within-cluster climate variation.

We allowed year to interact with climate and the ecological variables (Eq. 2). The climate-year interaction was added due to strong interactive effects reported in a previous study^2^, which used a large portion of the data used here. For these analyses, the LMM were fitted using Maximum Likelihood because model selection, detailed below, can be biased by REML.

1 $$\begin{array}{cc}{{\rm{Carbon}}}\\ {{\rm{Vegetation}}} & \sim {{\rm{Year}}}+\left(1\,|{{\rm{Site}}}\right)\\ {{\rm{Climate}}}\end{array}$$

2 $$\begin{array}{cc}\begin{array}{c}{{\rm{Carbon\ stocks}}}\\ {{\rm{Carbon\ dynamics}}}\\ {{\rm{Carbon\ dynamics}}}(\%)\end{array} & \sim {{\rm{S}}}{{\rm{ite\,area}}}+{{\rm{Soil}}}+\left({{\rm{Climate}}}+{{\rm{T}}}.{{\rm{diversity}}}+{{\rm{P}}}.{{\rm{diversity}}}+{{\rm{Trait\,dispersion}}}+{{\rm{Trait\,CWM}}}\right) \, * \, {{\rm{Year}}}+(1{{\rm{|Site}}})\end{array}$$

In the equations above, “*” is an interaction term, which includes the main effects of each separate variable and their interaction effects, “+” is the additive effects (i.e., main effects) and “(1 | site)” refers to the random intercepts of sites.

Model selection was based on the Akaike Information Criterion of second order (AICc). We followed a three-step procedure. First, starting from a global model including all explanatory variables, we fitted submodels with all possible combinations of the following predictors: ecological variables, year, site area, climate, and soil (Eq. 2), year, site area, soil, and climate were included in all submodels to control for their effects. Second, we ranked the submodels by their AICc values. Third, through multimodel inference, we averaged the coefficients of the best-performing submodels (ΔAICc ≤ 4) and used their conditional averages in the final model^90^. To avoid multicollinearity (Figs. S9–S24), we only retained predictors with VIF ≤ 5, except for those involved in interaction terms, where a VIF < 10.5 was tolerated. This exception was necessary because interaction terms inherently increase VIF values due to variable repetition. In our models, this applied only to climate, which remained a strong predictor despite a high VIF. Although AICc penalises model complexity to reduce overfitting, we further constrained model complexity by requiring at least 10 observations per predictor.

We did not calculate the relative importance of predictors because Akaike weights are biased when predictors are not included in the same number of models, as was our case. For each final model (∆AICc = 0), we report the variance explained by the fixed effects (R²). Model selection, multimodel inference, and R² calculations were performed using the MuMIn package^91^. All effects sizes, *P*-values and 95% confidence intervals are reported in the main text, figures, or supplementary information.

### Reporting summary

Further information on research design is available in the Nature Portfolio Reporting Summary linked to this article.

## Supplementary information

Supplementary Information

Description of Additional Supplementary Information

Supplementary Data 1

Supplementary Data 2

Supplementary Data 3

Supplementary Data 4

Reporting Summary

Transparent Peer Review file

## Source data

Source Data

## Data Availability

All processed data supporting the findings of this study are available in the Supplementary Data files. Census-level data are provided in Supplementary Data 3, and census-interval data in Supplementary Data 4. The raw tree-level forest inventory data are available under restricted access, as they are subject to third-party data-use agreements defined by ForestPlots.net (www.forestplots.net; see area codes in Supplementary Data 1). Access can be obtained by submitting a request directly to ForestPlots.net in accordance with their data access policies. These raw data are not publicly available. Climate data were obtained from the Climatic Research Unit (CRU TS version 4.04; released 24 April 2020; https://crudata.uea.ac.uk/cru/data/hrg) and WorldClim (https://www.worldclim.org/). Phylogenetic data were obtained from Smith and Brown (^68^; https://doi.org/10.1002/ajb2.1019). Trait data were compiled from published and publicly available sources: Prado-Junior et al. (^12^; https://doi.org/10.1111/1365-2745.12543), Zanne et al. (^58^; https://doi.org/10.5061/dryad.234), de Paula (^59^; https://repositorio.ufjf.br/jspui/handle/ufjf/10249), Batalha et al. (^61^; https://doi.org/10.1111/j.1600-0706.2011.19513.x), Batalha et al. (^61^; https://doi.org/10.1016/j.flora.2011.07.004), Cianciaruso et al. (^62^; https://doi.org/10.1016/j.ppees.2011.11.004), Dantas and Pausas (^63^; https://doi.org/10.1111/geb.13111), Cianciaruso et al. (^64^; https://doi.org/10.1016/j.baae.2013.05.002), and Raymundo et al. (^65^; https://doi.org/10.1111/1365-2745.13031). Map data were obtained from the Instituto Brasileiro de Geografia e Estatística (IBGE; https://www.ibge.gov.br/geociencias/organizacao-do-territorio/malhas-territoriais/15774-malhas.html?=&t=acesso-ao-produto). Source data are provided with this paper.

## Code availability

The code is publicly available at https://doi.org/10.5281/zenodo.18881288

## References

[1] Hubau, W. et al. Asynchronous Carbon Sink Saturation in African and Amazonian Tropical Forests. *Nature* **579**, 80–87 (2020). 32132693 10.1038/s41586-020-2035-0 PMC7617213

[2] Maia, V. M. et al. The carbon sink of tropical seasonal forests in southeastern Brazil can be under threat. *Sci. Adv.* **6**, eabd4548 (2020). 33355136 10.1126/sciadv.abd4548 PMC11206208

[3] Poorter, L. et al. Biodiversity and climate determine the functioning of Neotropical forests. *Glob. Ecol. Biogeogr.* **27**, 389–390 (2017).

[4] Aleixo, I. et al. Amazonian rainforest tree mortality driven by climate and functional traits. *Nat. Clim. Change* **9**, 384–388 (2019).

[5] Ali, A. et al. Climate and soils determine aboveground biomass indirectly via species diversity and stand structural complexity in tropical forests. *For. Ecol. Manag.* **432**, 823–831 (2019).

[6] Aguirre-Gutiérrez, J. et al. Functional susceptibility of tropical forests to climate change. *Nat. Ecol. Evol.* **6**, 878–889 (2022). 35577983 10.1038/s41559-022-01747-6

[7] Langan, L., Scheiter, S., Hickler, T. & Higgins, S. I. Amazon forest resistance to drought is increased by diversity in hydraulic traits. *Nat. Commun.* **16**, 8246 (2025). 10.1038/s41467-025-63600-1 PMC12420827 40925888

[8] Coelho de Souza, F. et al. Evolutionary diversity is associated with wood productivity in Amazonian forests. *Nat. Ecol. Evol.* **3**, 1754–1761 (2019). 31712699 10.1038/s41559-019-1007-y

[9] McDowell, N. et al. Drivers and mechanisms of tree mortality in moist tropical forests. *N. Phytologist* **219**, 851–869 (2018). 10.1111/nph.15027 29451313

[10] Ribeiro, E. M. S. et al. Functional diversity and composition of Caatinga woody flora are negatively impacted by chronic anthropogenic disturbance. *J. Ecol.* **107**, 2291–2302 (2019).

[11] Klipel, J. et al. & others. How do distinct facets of tree diversity and community assembly respond to environmental variables in the subtropical Atlantic Forest? *Ecol. Evol.* **13**, e10321 (2023). 37465611 10.1002/ece3.10321 PMC10350641

[12] Prado-Junior, J. A. et al. Conservative species drive biomass productivity in tropical dry forests. *J. Ecol.* **104**, 817–827 (2016).

[13] Wang, L.-Q. & Ali, A. Climate regulates the functional traits – aboveground biomass relationships at a community-level in forests: A global meta-analysis. *Sci. Total Environ.* **761**, 143238 (2021). 33158541 10.1016/j.scitotenv.2020.143238

[14] Sullivan, M. J. P. et al. Long-term thermal sensitivity of Earth’s tropical forests. *Science* **368**, 869 (2020). 32439789 10.1126/science.aaw7578

[15] Phillips, O. L. et al. Drought Sensitivity of the Amazon Rainforest. *Science* **323**, 1344–1347 (2009). 19265020 10.1126/science.1164033

[16] Quesada, C. A. et al. Basin-wide variations in Amazon forest structure and function are mediated by both soils and climate. *Biogeosciences* **9**, 2203–2246 (2012).

[17] Vargas Gutiérrez, G. et al. Throughfall exclusion and fertilization effects on tropical dry forest tree plantations, a large-scale experiment. *Biogeosciences* **20**, 2143–2160 (2023).

[18] Banbury Morgan, R. et al. Global patterns of forest autotrophic carbon fluxes. *Glob. Change Biol.* **27**, 2840–2855 (2021). 10.1111/gcb.15574 33651480

[19] Doughty, C. E. et al. Tropical forests are approaching critical temperature thresholds. *Nature* **621**, 105–111 (2023). 37612501 10.1038/s41586-023-06391-z

[20] Tilman, D. The Ecological Consequences Of Changes In Biodiversity: A Search For General Principles. *Ecology* **80**, 1455–1474 (1999).

[21] Díaz, S. et al. Linking functional diversity and social actor strategies in a framework for interdisciplinary analysis of nature’s benefits to society. *Proc. Natl. Acad. Sci. USA* **108**, 895–902 (2011). 21220325 10.1073/pnas.1017993108 PMC3024663

[22] Loreau, M. & de Mazancourt, C. Biodiversity and ecosystem stability: a synthesis of underlying mechanisms. *Ecol. Lett.* **16**, 106–115 (2013). 23346947 10.1111/ele.12073

[23] Srivastava, D. S., Cadotte, M. W., MacDonald, A. A. M., Marushia, R. G. & Mirotchnick, N. Phylogenetic diversity and the functioning of ecosystems. *Ecol. Lett.* **15**, 637–648 (2012). 22583836 10.1111/j.1461-0248.2012.01795.x

[24] Yachi, S. & Loreau, M. Biodiversity and ecosystem productivity in a fluctuating environment: the insurance hypothesis. *Proc. Natl. Acad. Sci. USA* **96**, 1463–1468 (1999). 9990046 10.1073/pnas.96.4.1463 PMC15485

[25] Grime, J. P. Benefits of plant diversity to ecosystems: immediate, filter and founder effects. *J. Ecol.* **86**, 902–910 (1998).

[26] Van der Sande, M. T. et al. Soil fertility and species traits, but not diversity, drive productivity and biomass stocks in a Guyanese tropical rainforest. *Funct. Ecol.* **32**, 461–474 (2017).

[27] Conti, G. & Díaz, S. Plant functional diversity and carbon storage – an empirical test in semi-arid forest ecosystems. *J. Ecol.* **101**, 18–28 (2013).

[28] Greenwood, S. et al. Tree mortality across biomes is promoted by drought intensity, lower wood density and higher specific leaf area. *Ecol. Lett.* **20**, 539–553 (2017). 28220612 10.1111/ele.12748

[29] Poorter, L., Castilho, C. V., Schietti, J., Oliveira, R. S. & Costa, F. R. C. Can traits predict individual growth performance? A test in a hyperdiverse tropical forest. *N. Phytologist* **219**, 109–121 (2018). 10.1111/nph.15206 PMC6001574 29774944

[30] Santiago, L. S. et al. Leaf photosynthetic traits scale with hydraulic conductivity and wood density in Panamanian forest canopy trees. *Oecologia* **140**, 543–550 (2004). 15232729 10.1007/s00442-004-1624-1

[31] Poorter, L. & Bongers, F. Leaf traits are good predictors of plant performance across 53 rain forest species. *Ecology* **87**, 1733–1743 (2006). 16922323 10.1890/0012-9658(2006)87[1733:ltagpo]2.0.co;2

[32] Chave, J. et al. Towards a worldwide wood economics spectrum. *Ecol. Lett.* **12**, 351–366 (2009). 19243406 10.1111/j.1461-0248.2009.01285.x

[33] Reich, P. B. et al. The evolution of plant functional variation: traits, spectra, and strategies. *Int. J. Plant Sci.* **164**, S143–S164 (2003).

[34] Wright, S. J. et al. Functional traits and the growth–mortality trade-off in tropical trees. *Ecology* **91**, 3664–3674 (2010). 21302837 10.1890/09-2335.1

[35] Oliveira, R. S. et al. Linking plant hydraulics and the fast–slow continuum to understand resilience to drought in tropical ecosystems. *N. Phytologist* **230**, 904–923 (2021). 10.1111/nph.17266 33570772

[36] Pineda-García, F., Paz, H. & Meinzer, F. C. Drought resistance in early and late secondary successional species from a tropical dry forest: the interplay between xylem resistance to embolism, sapwood water storage and leaf shedding. *Plant, Cell Environ.* **36**, 405–418 (2013). 22812458 10.1111/j.1365-3040.2012.02582.x

[37] Allen, K. et al. Will seasonally dry tropical forests be sensitive or resistant to future changes in rainfall regimes? *Environ. Res. Lett.* **12**, 023001 (2017).

[38] Bauman, D. et al. Tropical tree growth sensitivity to climate is driven by species intrinsic growth rate and leaf traits. *Glob. Change Biol.* **28**, 1414–1432 (2022). 10.1111/gcb.15982 34741793

[39] Gomes, V. H. F., Vieira, I. C. G., Salomão, R. P. & ter Steege, H. Amazonian tree species threatened by deforestation and climate change. *Nat. Clim. Change* **9**, 547–553 (2019).

[40] Vincent, H., Bornand, C. N., Kempel, A. & Fischer, M. Rare species perform worse than widespread species under changed climate. *Biol. Conserv.* **246**, 108586 (2020).

[41] John, R. et al. Soil nutrients influence spatial distributions of tropical tree species. *Proc. Natl. Acad. Sci.* **104**, 864–869 (2007). 17215353 10.1073/pnas.0604666104 PMC1783405

[42] Cleveland, C. C. et al. Relationships among net primary productivity, nutrients and climate in tropical rain forest: a pan-tropical analysis. *Ecol. Lett.* **14**, 939–947 (2011). 21749602 10.1111/j.1461-0248.2011.01658.x

[43] Wieder, W. et al. Future productivity and carbon storage limited by terrestrial nutrient availability. *Nat. Geosci.* **8**, 441–444 (2015).

[44] Soong, J. L. et al. Soil properties explain tree growth and mortality, but not biomass, across phosphorus-depleted tropical forests. *Sci. Rep.* **10**, 2302 (2020). 32041976 10.1038/s41598-020-58913-8 PMC7010742

[45] Wright, S. J. et al. Potassium, phosphorus, or nitrogen limit root allocation, tree growth, or litter production in a lowland tropical forest. *Ecology* **92**, 1616–1625 (2011). 21905428 10.1890/10-1558.1

[46] Muller-Landau, H. C. et al. Patterns and mechanisms of spatial variation in tropical forest productivity, woody residence time, and biomass. *N. Phytologist* **229**, 3065–3087 (2020). 10.1111/nph.17084 33207007

[47] Malhi, Y. The productivity, metabolism and carbon cycle of tropical forest vegetation. *J. Ecol.* **100**, 65–75 (2012).

[48] Gilbert, G. S. & Parker, I. M. The evolutionary ecology of plant disease: a phylogenetic perspective. *Annu. Rev. Phytopathol.* **54**, 549–578 (2016). 27359365 10.1146/annurev-phyto-102313-045959

[49] MacDicken, K. G., G. V. Wolf, and C. B. Briscoe. Standard research methods for multipurpose trees and shrubs. Winrock International. (1991).

[50] Angiosperm Phylogeny Group An update of the Angiosperm Phylogeny Group classification for the orders and families of flowering plants: APG IV. *Botanical J. Linnean Society*, **181**, 1–20 (2016).

[51] Carvalho G. et al. Flora: Tools for Interacting with the Brazilian Flora 2020. R package version 0.3.4. https://CRAN.R-project.org/package=flora (2020).

[52] Flora do Brasil 2020 em construção. Jardim Botânico do Rio de Janeiro. Disponível em: <http://floradobrasil.jbrj.gov.br/>. Acesso em: 09 Mar. 2020.

[53] Réjou-Méchain, M., Tanguy, A., Piponiot, C., Chave, J. & Hérault, B. BIOMASS: an r package for estimating above-ground biomass and its uncertainty in tropical forests (version 2.2.4-1). *Methods Ecol. Evol.* **8**, 1163–1167 (2017).

[54] Martin, A. R., Doraisami, M. & Thomas, S. C. Global patterns in wood carbon concentration across the world’s trees and forests. *Nat. Geosci.* **11**, 915–920 (2018).

[55] Talbot, J. et al. Methods to estimate aboveground wood productivity from long-term forest inventory plots. *For. Ecol. Manag.* **320**, 30–38 (2014).

[56] Chao, A. et al. Rarefaction and extrapolation with Hill numbers: a framework for sampling and estimation in species diversity studies. *Ecol. Monogr.* **84**, 45–67 (2014).

[57] Hsieh T. C., Ma K. H. and Chao A. iNEXT: iNterpolation and EXTrapolation for species diversity. R package version 2.0.20 URL: http://chao.stat.nthu.edu.tw/wordpress/software-download/ (2020).

[58] Zanne, A. E. et al. Towards a Worldwide Wood Economics Spectrum 10.5061/dryad.234 (Dryad Digital Repository) (2009). 19243406

[59] Paula, M. A. de. Bamboo dominance in an Atlantic Forest community: a structural, functional and taxonomic approach. Universidade Federal de Juiz de Fora https://repositorio.ufjf.br/jspui/handle/ufjf/10249 (2019).

[60] Batalha, M. A., Silva, I. A., Cianciaruso, M. V. & de Carvalho, G. H. Trait diversity on the phylogeny of cerrado woody species. *Oikos* **120**, 1741–1751 (2011a).

[61] Batalha, M. A., Silva, I. A., Cianciaruso, M. V., França, H. & de Carvalho, G. H. Phylogeny, traits, environment, and space in cerrado plant communities at Emas National Park (Brazil). *Flora - Morphol., Distrib., Funct. Ecol. Plants* **206**, 949–956 (2011b).

[62] Cianciaruso, M. V., Silva, I. A., Batalha, M. A., Gaston, K. J. & Petchey, O. L. The influence of fire on phylogenetic and functional structure of woody savannas: Moving from species to individuals. *Perspect. Plant Ecol., Evol. Syst.* **14**, 205–216 (2012).

[63] Dantas, V. L. & Pausas, J. G. Megafauna biogeography explains plant functional trait variability in the tropics. *Glob. Ecol. Biogeogr.* **29**, 1288–1298 (2020).

[64] Cianciaruso, M. V., Silva, I. A., Manica, L. T. & Souza, J. P. Leaf habit does not predict leaf functional traits in cerrado woody species. *Basic Appl. Ecol.* **14**, 404–412 (2013).

[65] Raymundo, D. et al. Shifting species and functional diversity due to abrupt changes in water availability in tropical dry forests. *J. Ecol.* **107**, 253–264 (2018).

[66] Pérez-Harguindeguy, N. et al. New handbookfor standardised measurement of plant functional traits worldwide. *Aust. J. Bot.* **61**, 715–716 (2013).

[67] Cayuela, L., Granzow-de la Cerda, Í, Albuquerque, F. S. & Golicher, D. J. taxonstand: An r package for species names standardisation in vegetation databases. *Methods Ecol. Evol.* **3**, 1078–1083 (2012).

[68] Smith, S. A. & Brown, J. W. Constructing a broadly inclusive seed plant phylogeny. *Am. J. Bot.* **105**, 302–314 (2018). 29746720 10.1002/ajb2.1019

[69] Kembel, S. W. et al. Picante: R tools for integrating phylogenies and ecology (version 1.8.2). *Bioinformatics* **26**, 1463–1464 (2010). 20395285 10.1093/bioinformatics/btq166

[70] Faith, D. P. Conservation evaluation and phylogenetic diversity. *Biol. Conserv.* **61**, 1–10 (1992).

[71] Honorio Coronado, E. N. et al. Phylogenetic diversity of Amazonian tree communities. *Diversity Distrib.* **21**, 1295–1307 (2015).

[72] Harris, I. et al. Version 4 of the CRU TS monthly high-resolution gridded multivariate climate dataset. *Sci. Data* **7**, 109 (2020). 32246091 10.1038/s41597-020-0453-3 PMC7125108

[73] Fick, S. E. & Hijmans, R. J WorldClim 2: new 1-km spatial resolution climate surfaces for global land areas. *Int. J. Climatol.* **37**, 4302–4315 (2017).

[74] Peng, S., Ding, Y., Liu, W. & Li, Z. *Earth Syst. Sci. Data* **11**, 1931–1946 (2019).

[75] Chave, J. et al. Improved allometric models to estimate the aboveground biomass of tropical trees. *Glob. Change Biol.* **20**, 3177–3190 (2014). 10.1111/gcb.12629 24817483

[76] EMBRAPA. Manual de métodos de análise de solos (2nd ed.). Rio de Janeiro: EMBRAPA/Centro Nacional de Pesquisa de Solos (1997).

[77] Magnago, L. F. S. et al. Microclimatic conditions at forest edges have significant impacts on vegetation structure in large Atlantic forest fragments. *Biodivers. Conserv.* **24**, 2305–2318 (2015).

[78] Nunes, M. H. et al. Forest fragmentation impacts the seasonality of Amazonian evergreen canopies. *Nat. Commun.* **13**, 917 (2022). 35177619 10.1038/s41467-022-28490-7 PMC8854568

[79] R. Core Team. R: A language and environment for statistical computing. Version 4.4.1. R Foundation for Statistical Computing, Vienna, Austria. https://www.r-project.org/ (2024).

[80] Wickham, H. Ggplot2: Elegant Graphics for Data Analysis (version 4.0.1). Springer-Verlag New York. ISBN 978-3-319-24277-4, https://ggplot2.tidyverse.org (2016).

[81] Wei, T., & Simko, V. R package “corrplot”: Visualization of a Correlation Matrix (Version 0.84). Retrieved from https://github.com/taiyun/corrplot (2017).

[82] Garnier, S. Viridis: Default Color Maps from ‘matplotlib’. R package version 0.5.1. https://CRAN.R-project.org/package=viridis (2018).

[83] Wilke, C. Cowplot: Streamlined Plot Theme and Plot Annotations for ‘ggplot2’. R package version 1.0.0. https://CRAN.R-project.org/package=cowplot. (2019).

[84] Schloerke, B. et al. GGally: Extension to ‘ggplot2’. R package version 2.1.1. https://CRAN.R-project.org/package=GGally (2021).

[85] Bates, D., Mächler, M., Bolker, B. & Walker, S. Fitting linear mixed-effects models using lme4 (version 1.1-37). *J. Stat. Softw.* **67**, 1–48 (2015).

[86] Wood, S. Generalized Additive Models: An Introduction with R, 2 edition (mgcv version 1.9-1). Chapman and Hall/CRC. (2017).

[87] Bivand R. S., Pebesma E., Gomez-Rubio V. Applied spatial data analysis with R, Second edition (spdep version 1.4-2). Springer, NY. http://www.asdar-book.org/ (2013).

[88] Lê, S., Josse, J., Husson, F. & FactoMineR:, A. Package for Multivariate Analysis (version 2.12). *J. Stat. Softw.* **25**, 1–18 (2008).

[89] Kassambara, A. & Mundt, F. Factoextra: Extract and Visualize the Results of Multivariate Data Analyses. R package version 1.0.7. https://CRAN.R-project.org/package=factoextra (2020).

[90] Burnham, K. P., Anderson, D. R. & Huyvaert, K. P. AIC model selection and multimodel inference in behavioral ecology: Some background, observations, and comparisons. *Behav. Ecol. Sociobiol.* **65**, 23–35 (2011).

[91] Bartón, K. MuMIn: Multi-Model Inference. R package version 1.42.1. Retrieved from https://cran.r-project.org/package=MuMIn (2018).

