# Supplementary material for: Long-term decline in above-ground carbon sinks in Brazilian tropical and subtropical forests
Source: Nat Commun. 2026 Aug 17;17:9856. doi: 10.1038/s41467-026-74921-0 (PMC13578287; doi:10.1038/s41467-026-74921-0)
Supplement: Supplementary file 1 [file 41467_2026_74921_MOESM1_ESM.pdf]

Supplementary Information

Long-term decline in above-ground carbon sinks in Brazilian tropical and subtropical forests

Supplementary figures

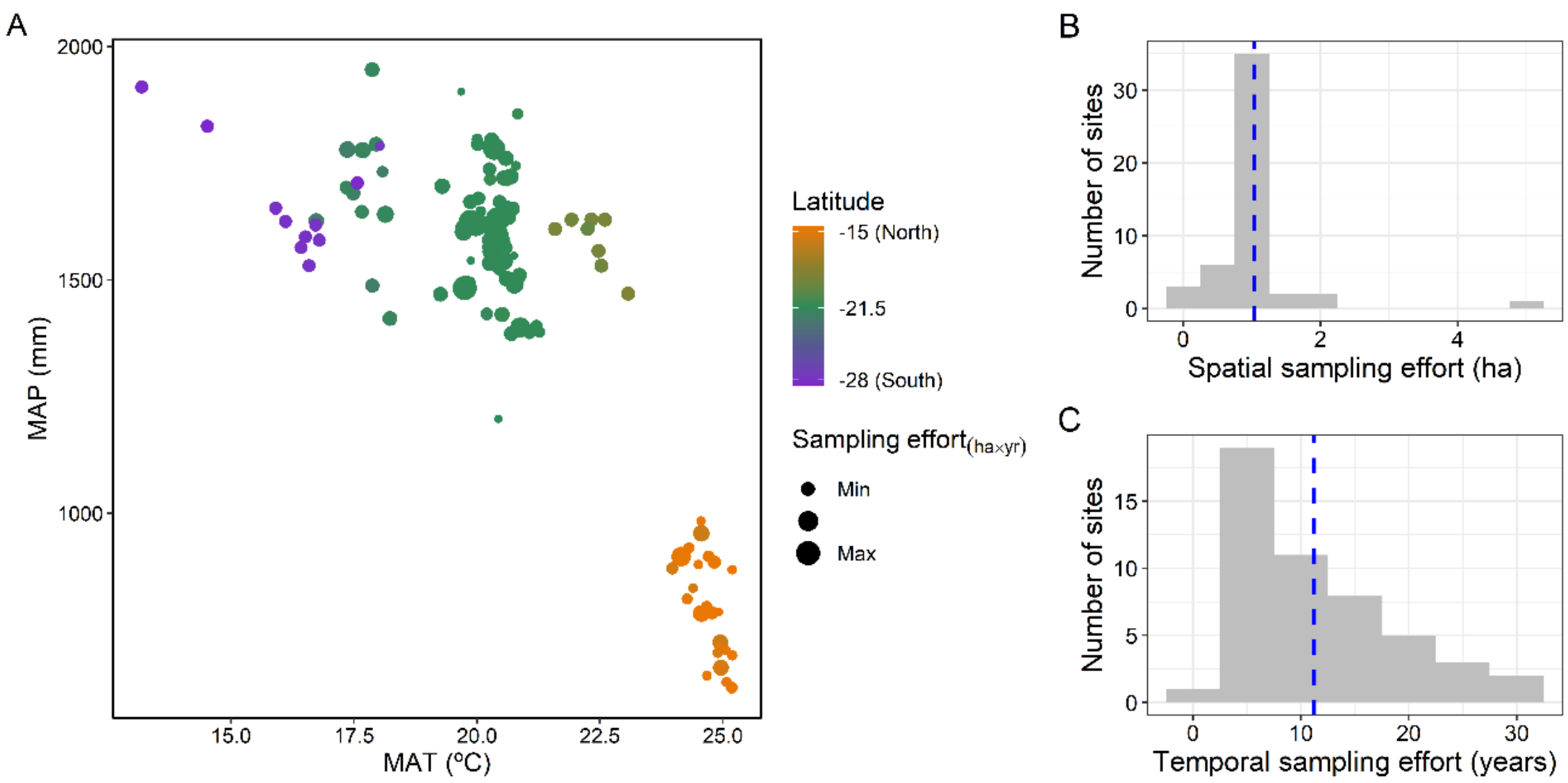

Figure S1. Distribution of the sampled sites across the climate space and their spatial and temporal sampling efforts. A) Distribution of the sites along the climate space represented by mean annual temperature (MAT) and mean annual precipitation (MAP); latitude is represented by a color gradient transitioning from purple to green to orange. The points are census intervals (n = 114), and their sizes are proportional to the site sampled area (mean 1.03 ha) times interval length (mean 5 years). B) Frequency of site sampled areas (n = 49). The blue dashed line is the mean of the sampled area among sites (1.03 ha). C) Frequency of site total monitoring length (n = 49) (year of the last census minus year of the first census). The blue dashed line is the mean of the total monitoring length among sites (11 years). Source data are provided as a Source Data file.

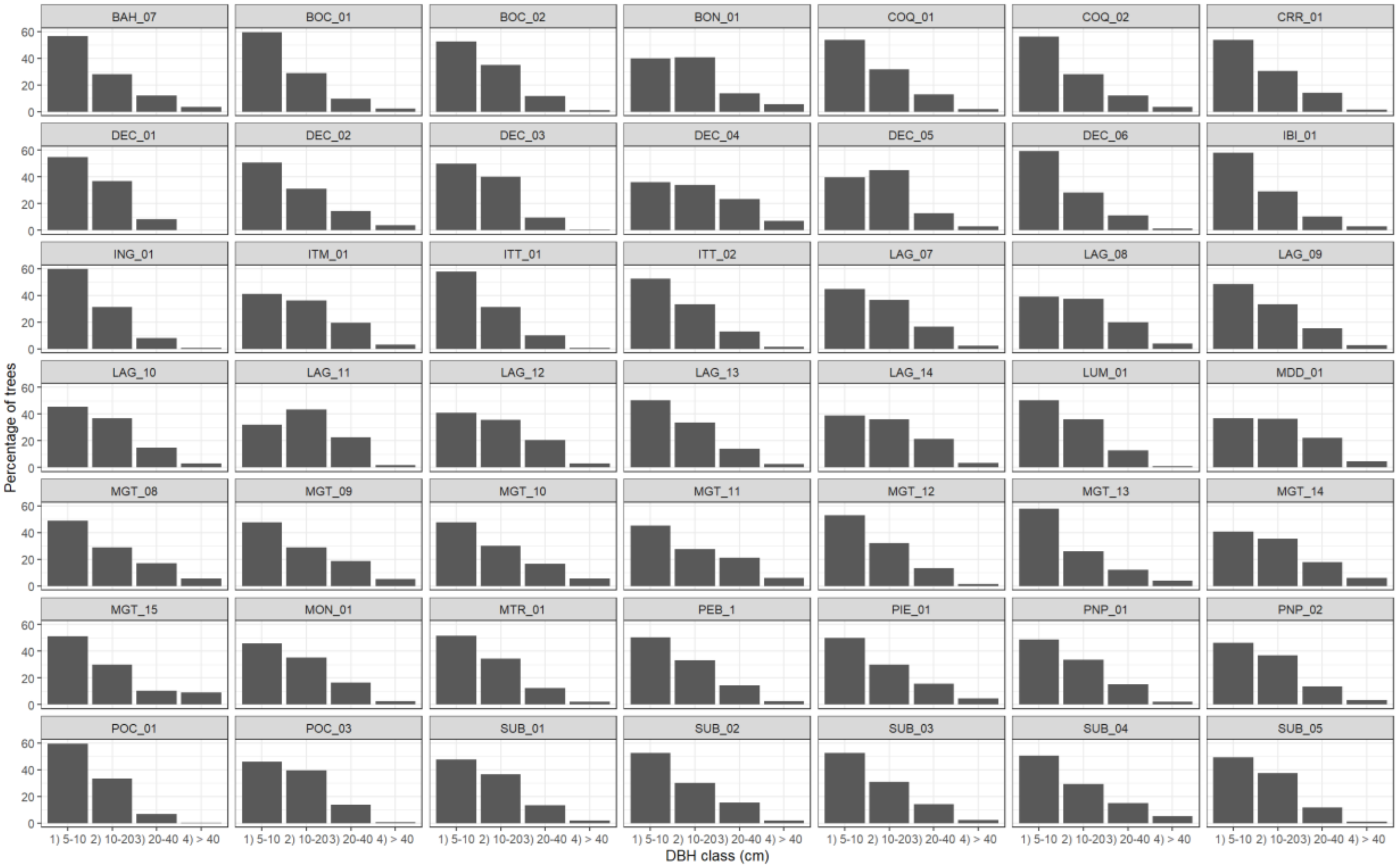

Figure S2. Diametric distribution of tree individuals by site. The y axis is the percentage of trees relative to the total number of trees in the site, n = 244329. Source data are provided as a Source Data file.

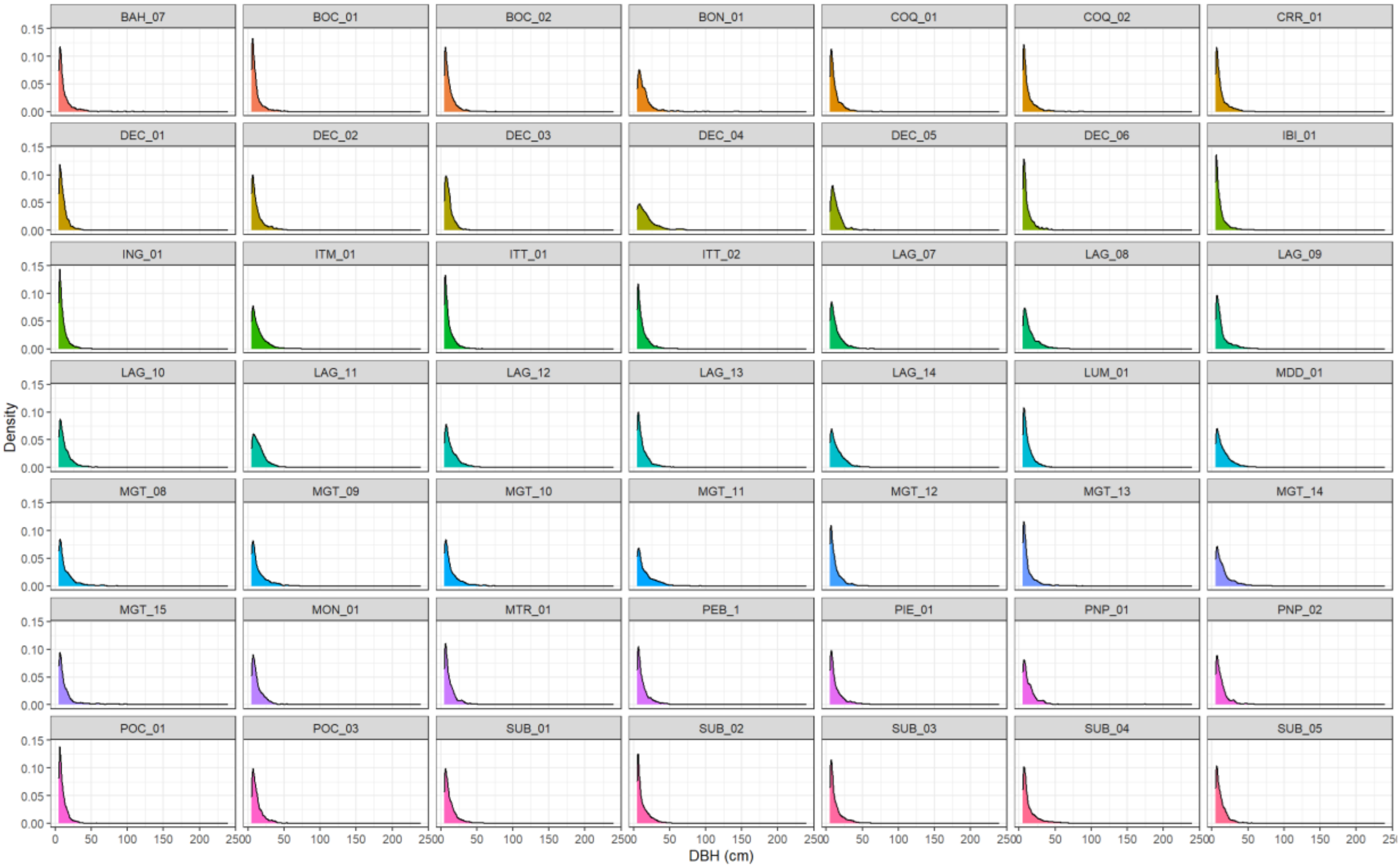

Figure S3. Density of tree individuals by DBH for each site, n = 244329. Source data are provided as a Source Data file.

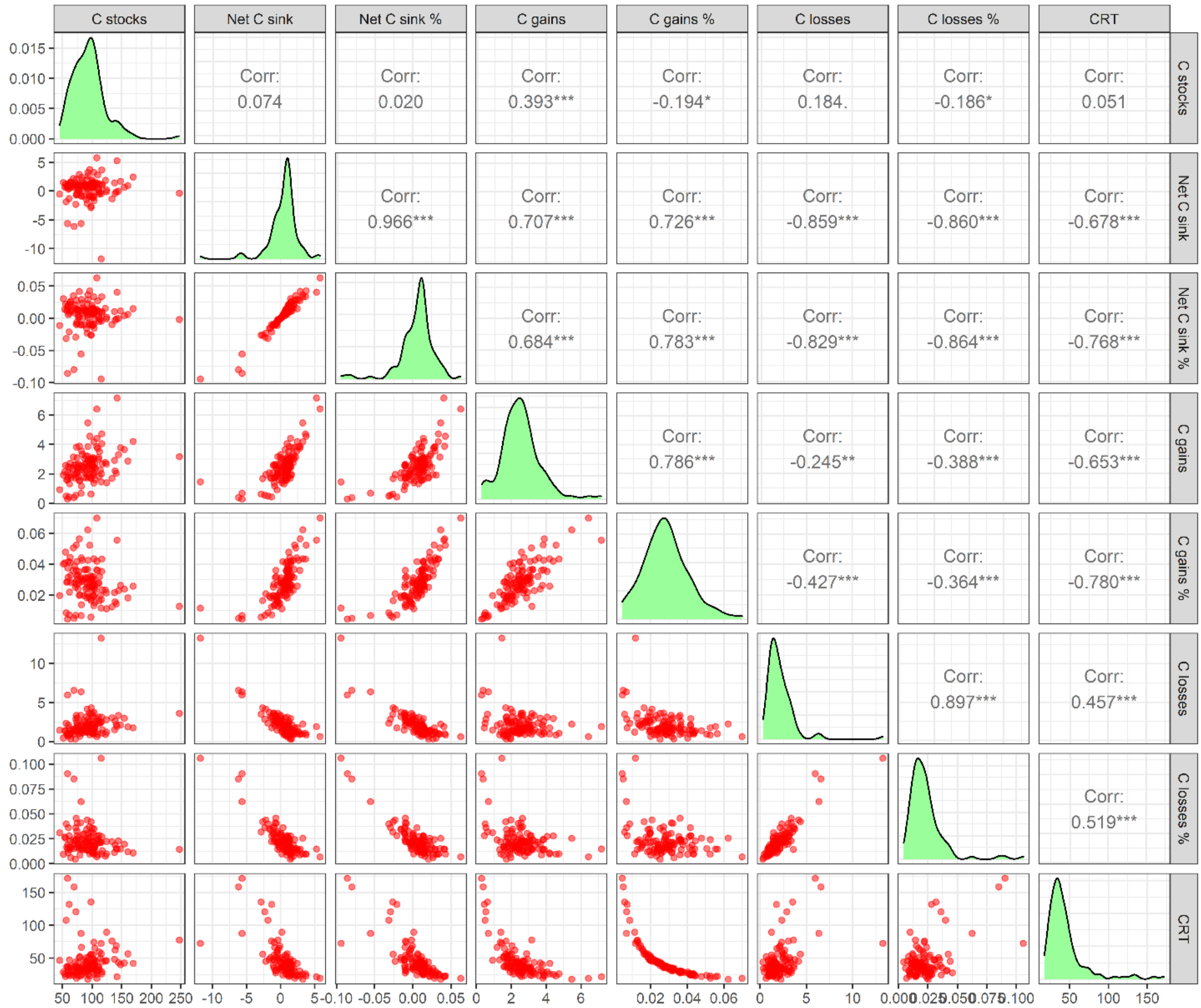

Figure S4. Correlation, scatter plots and density of each carbon dynamics variable. Variables followed by % are the values relativized by carbon stocks; n = 114. Source data are provided as a Source Data file.

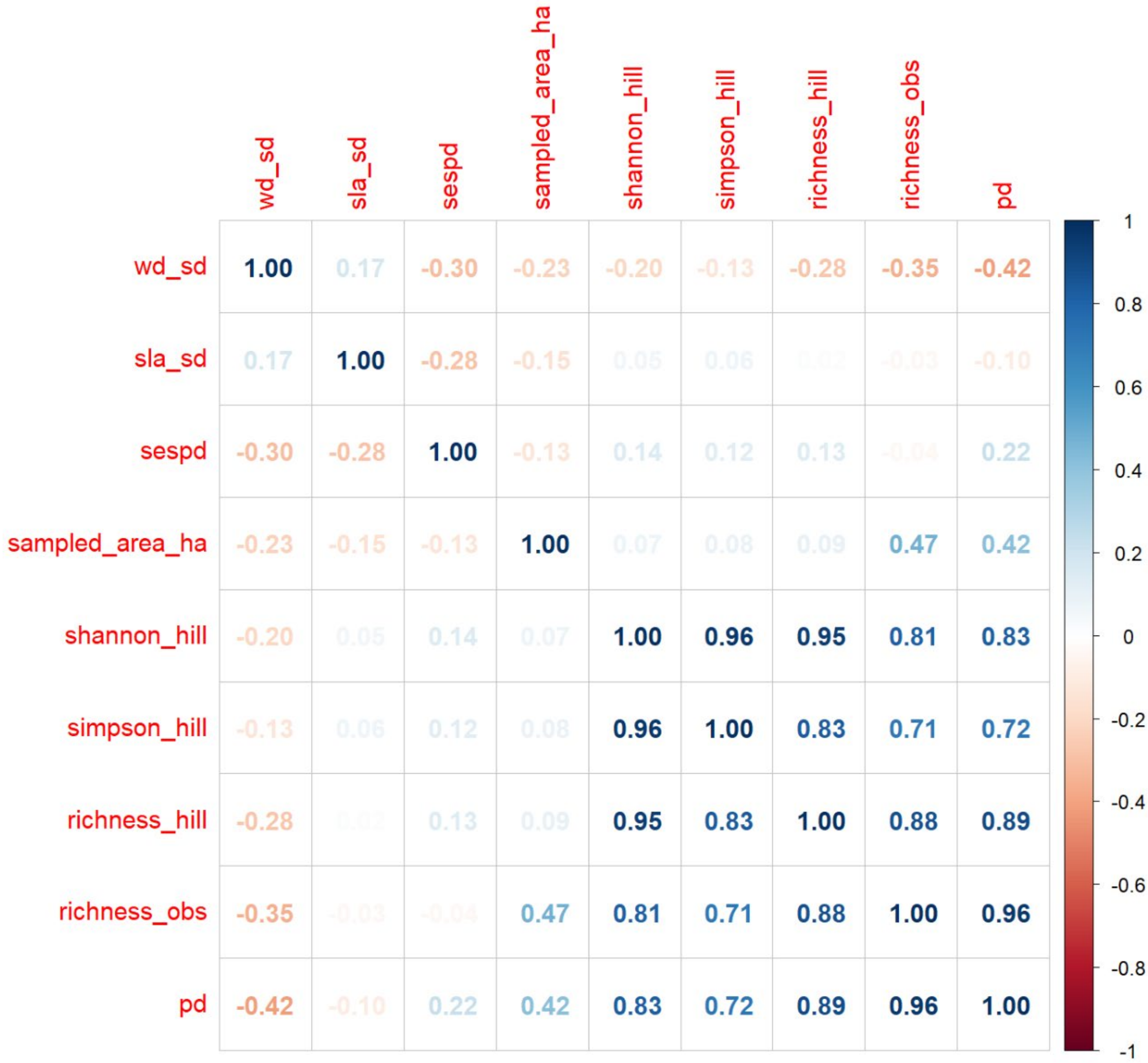

Figure S5. Correlation between ecological variables (including sampled area). Wood density dispersion (wd_sd), specific leaf area dispersion (sla_sd), phylogenetic diversity (standardized value; sespd), sampled area in hectares (sampled_area_ha), Shannon diversity Hill number (Shannon_hill), species richness Hill number (richness_hill), observed species richness (richness_obs) and phylogenetic diversity (PD; pd), n = 163. Source data are provided as a Source Data file.

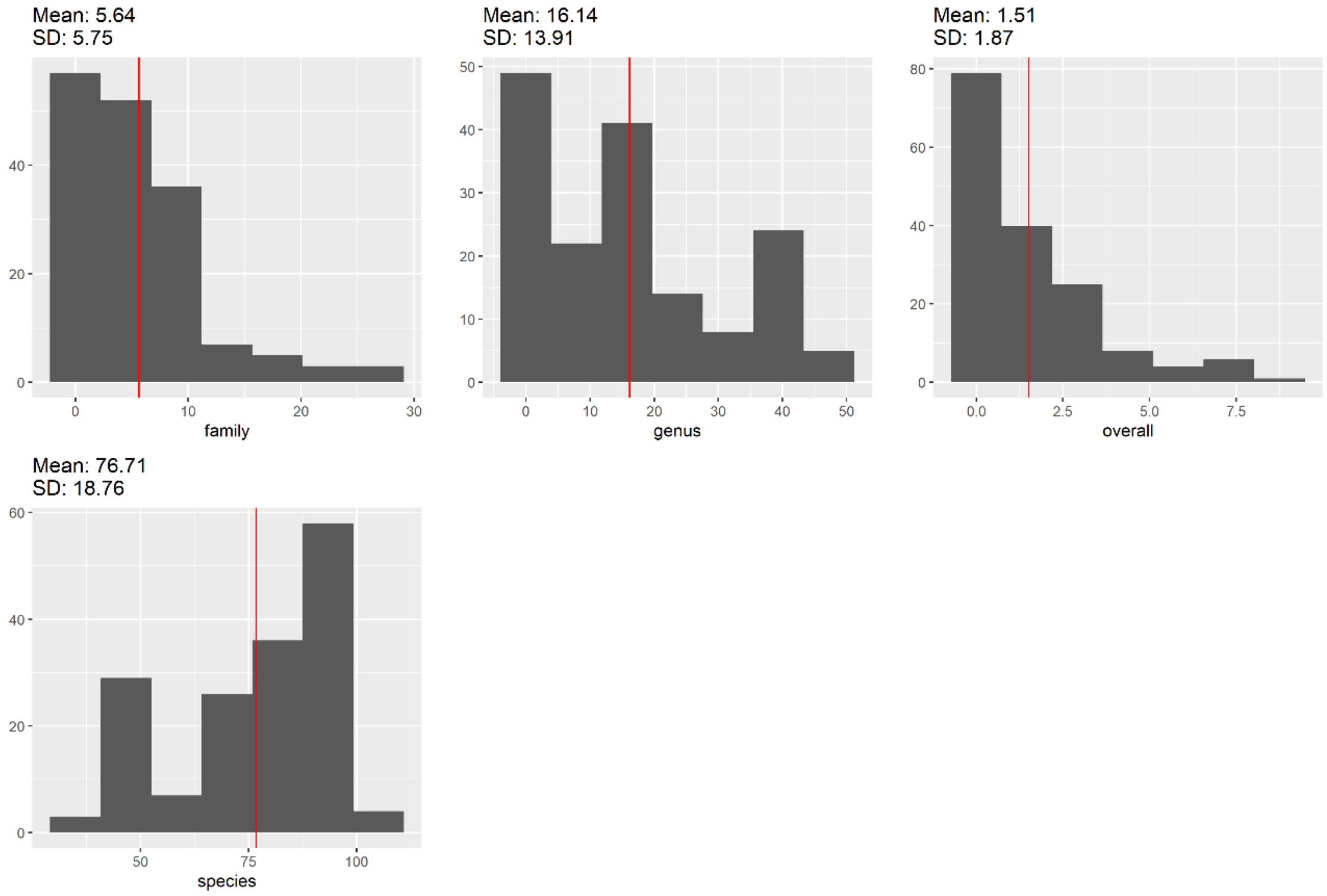

Figure S6. Distribution of the representativity (relative carbon stocks) of species-level trait information among census of specific leaf area (SLA), n = 163. Source data are provided as a Source Data file.

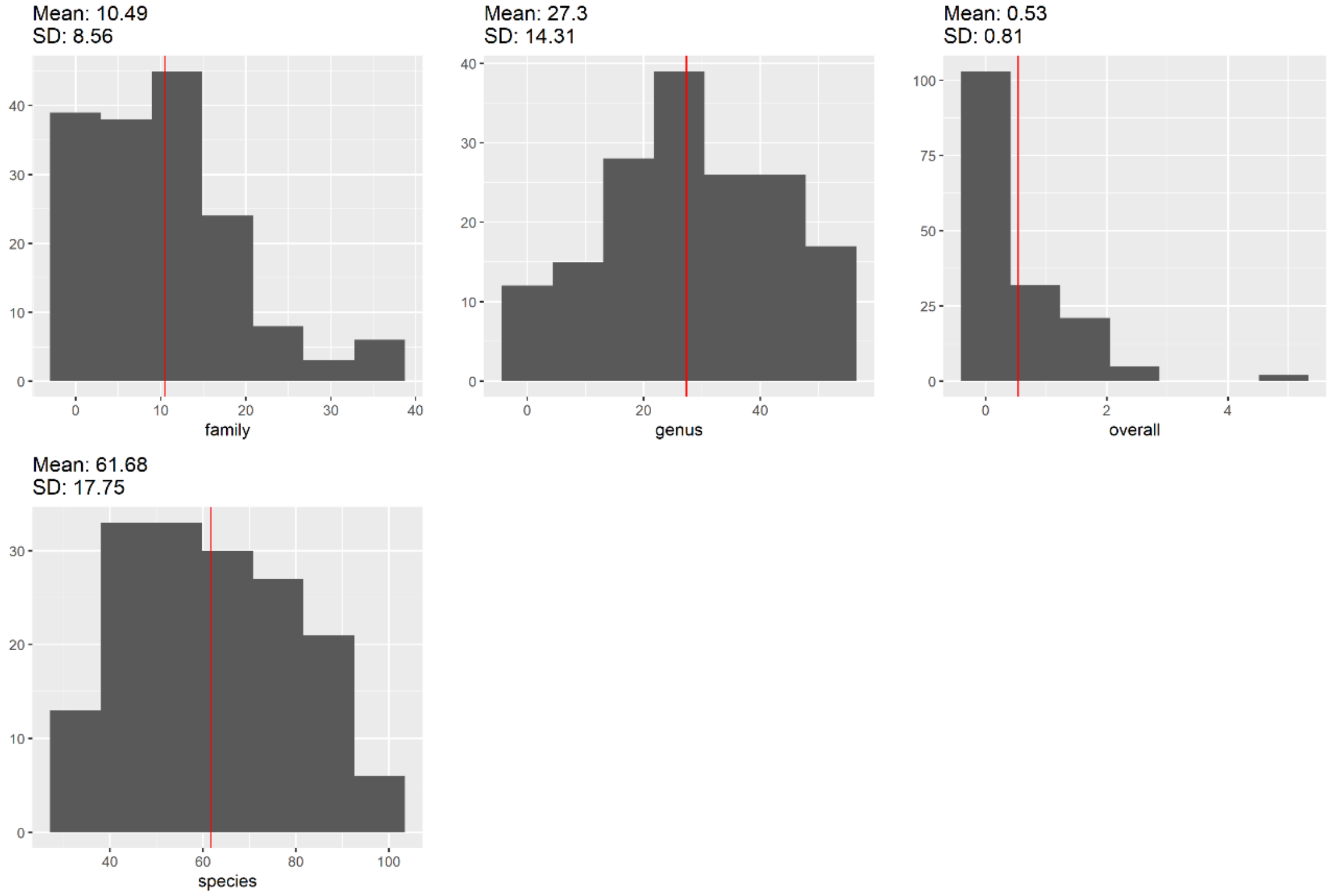

Figure S7. Distribution of the representativity (relative carbon stocks) of species-level trait information among census of wood density (WD), n = 163. Source data are provided as a Source Data file.

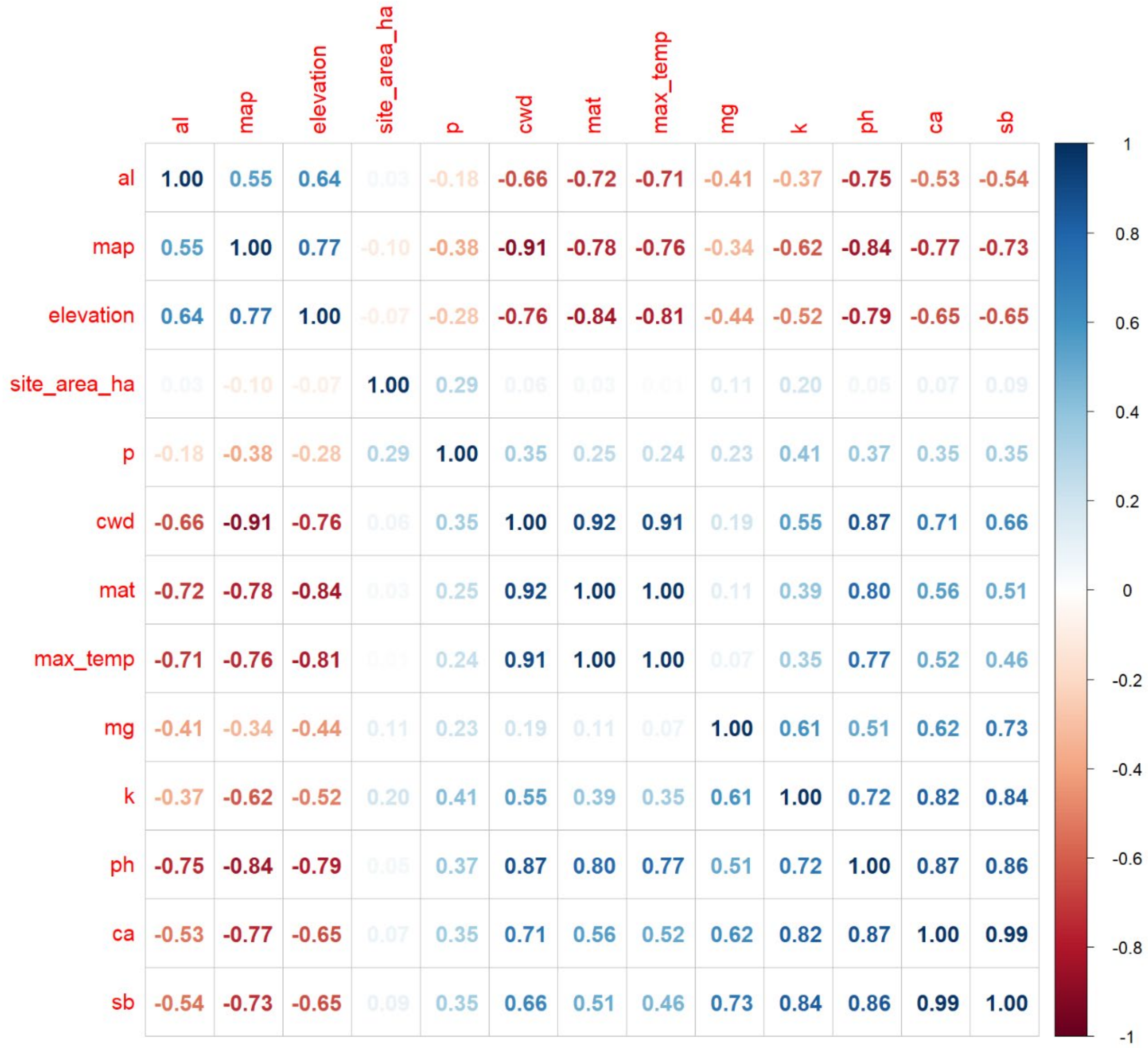

Figure S8. Correlation between environmental variables (climate and soil), altitude (elevation) and site area in hectares (site_area_ha). Mean Annual Temperature (mat), Maximum Temperature (max_temp), Mean Annual Precipitation (map), and Climatic Water Deficit (cwd), pH (ph), Phosphorus (p), Potassium (k), Calcium (ca), Magnesium (mg), Aluminum (al), and Base Saturation (sb), n = 163. Source data are provided as a Source Data file.

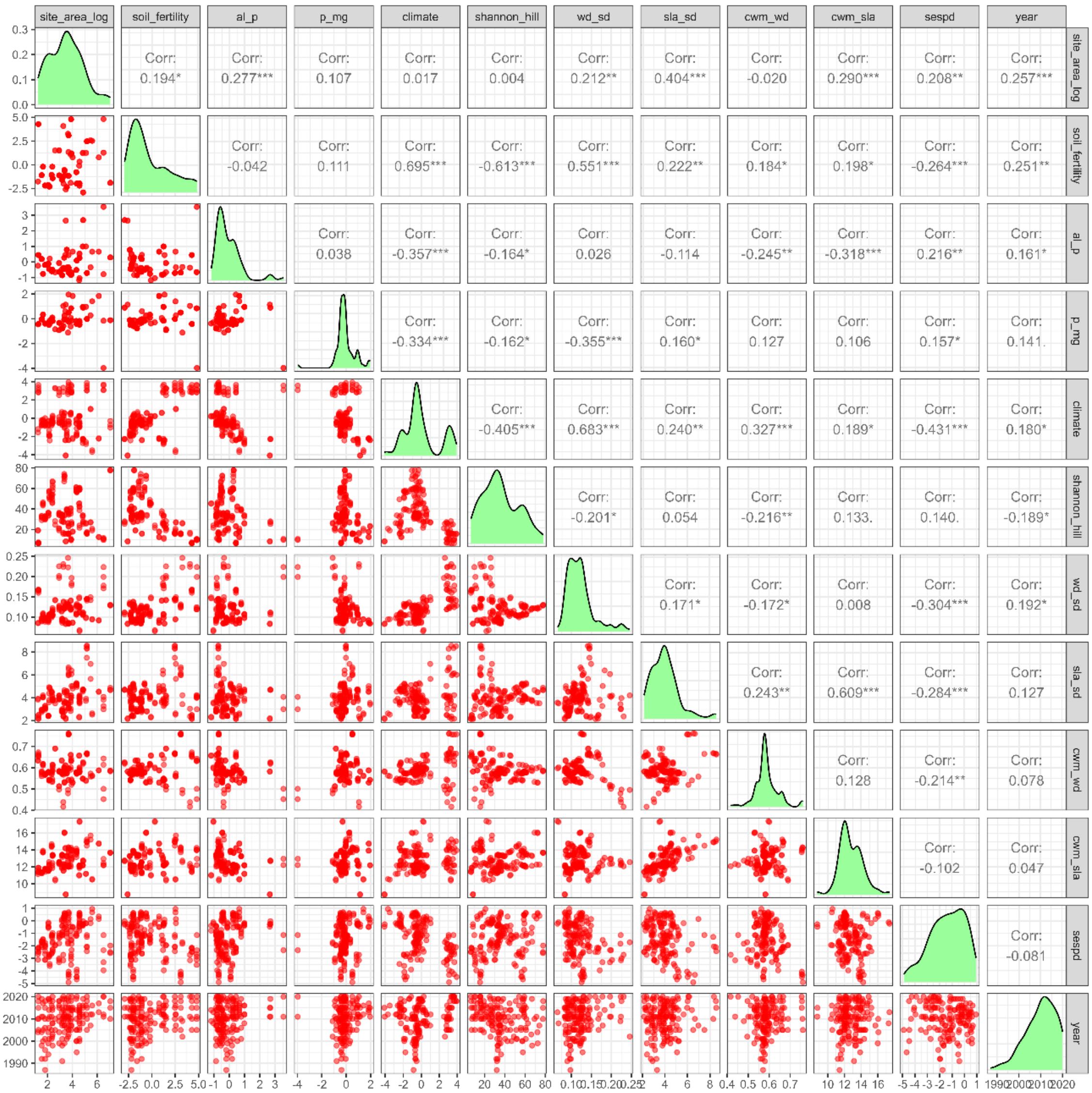

Figure S9. Correlation, scatter plots and density of the predictors of the carbon stocks model (n = 163). Site area in hectares in log scale (site_area_log), soil fertility PCA axis (soil_fertility), aliminum-phosphors PCA axis (al_p), phosphorus -> magnesium PCA axis (p_mg), cold-wet -> warm-dry PCA axis (climate), taxonomic diversity (shannon_hill), WD dispersion (wd_sd), SLA dispersion (sla_wd), community weighted mean of WD (wd_cwm), community weighted mean of SLA (sla_cwm), phylogenetic diversity (sespd) and year (year). Source data are provided as a Source Data file.

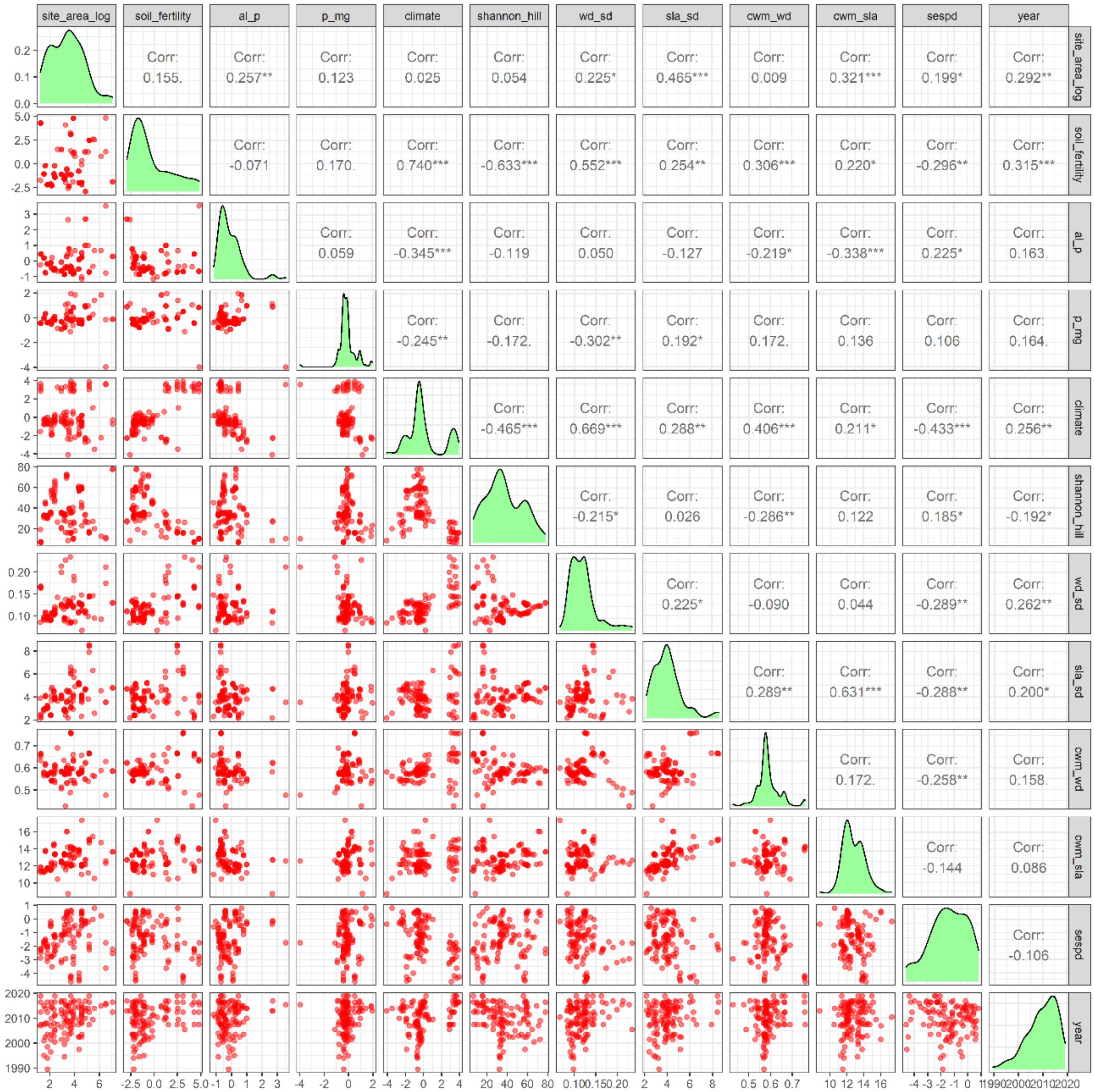

Figure S10. Correlation, scatter plots and density of the predictors of the carbon dynamics models (n = 114). Site area in hectares in log scale (site_area_log), soil fertility PCA axis (soil_fertility), aliminum-phosphors PCA axis (al_p), phosphorus -> magnesium PCA axis (p_mg), cold-wet -> warm-dry PCA axis (climate), taxonomic diversity (shannon_hill), WD dispersion (wd_sd), SLA dispersion (sla_wd), community weighted mean of WD (wd_cwm), community weighted mean of SLA (sla_cwm), phylogenetic diversity (sespd) and year (year). Source data are provided as a Source Data file.

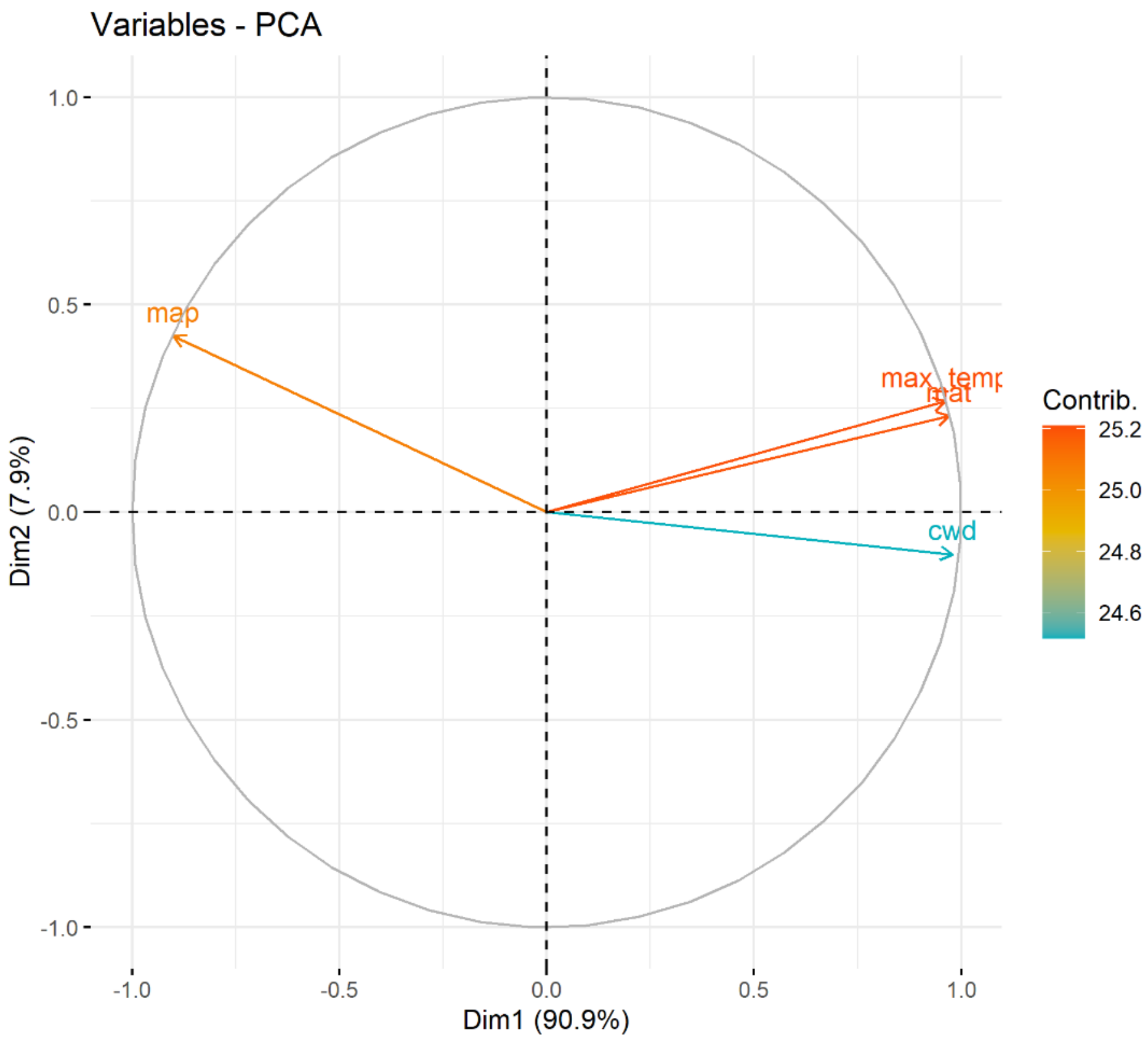

Figure S11. Principal Component Analysis (PCA) Biplot of Climate Variables at census level. This PCA biplot visualizes the relationship among four climate variables: Mean Annual Temperature (mat), Maximum Temperature (max_temp), Mean Annual Precipitation (map), and Climatic Water Deficit (cwd). The analysis encompasses data aggregated over complete years within each interval (n = 163). Source data are provided as a Source Data file.

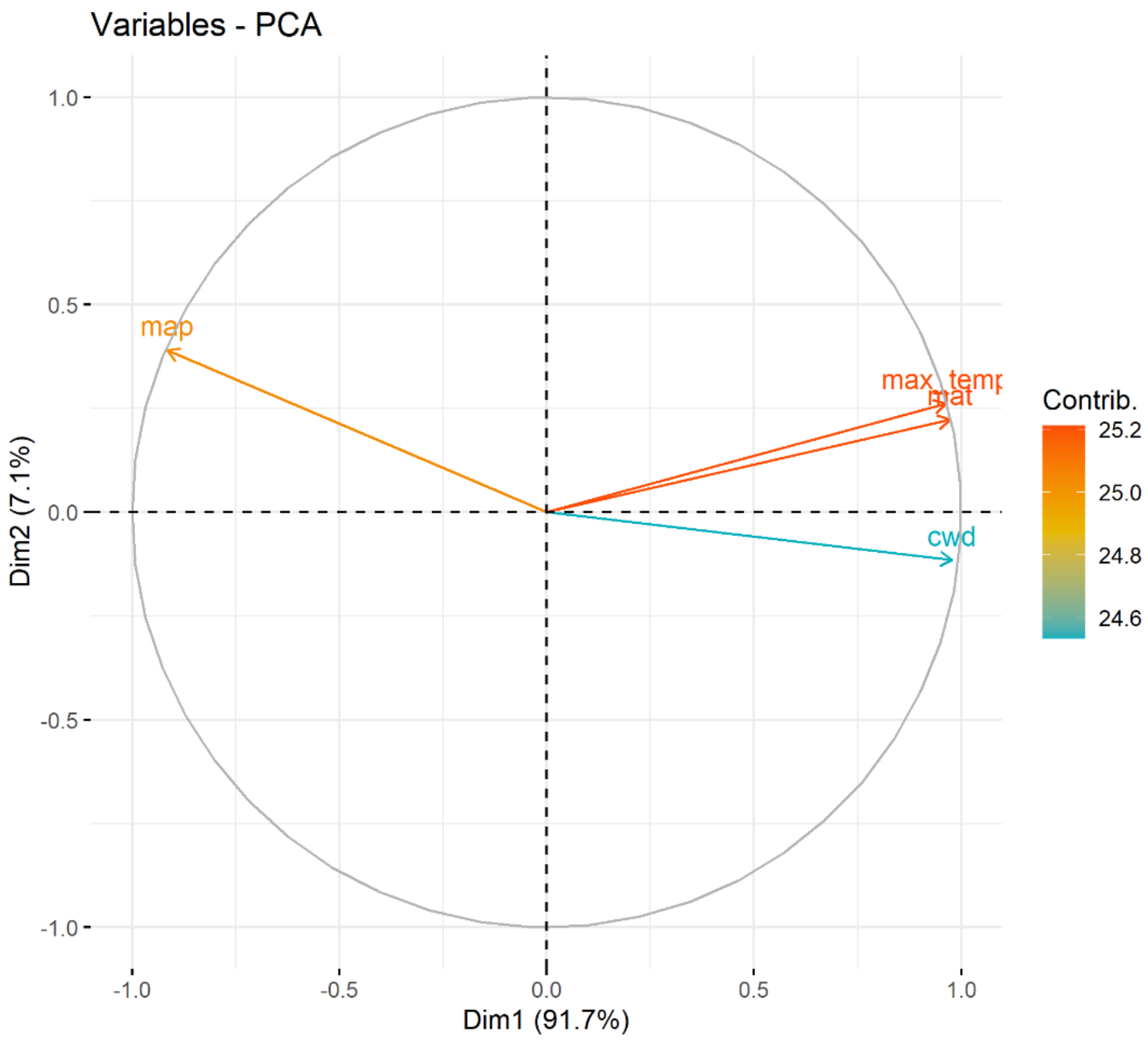

Figure S12. Principal Component Analysis (PCA) Biplot of Climate Variables at census interval level. This PCA biplot visualizes the relationship among four climate variables: Mean Annual Temperature (mat), Maximum Temperature (max_temp), Mean Annual Precipitation (map), and Climatic Water Deficit (cwd). The analysis encompasses data aggregated over complete years within each interval (n = 114). Source data are provided as a Source Data file.

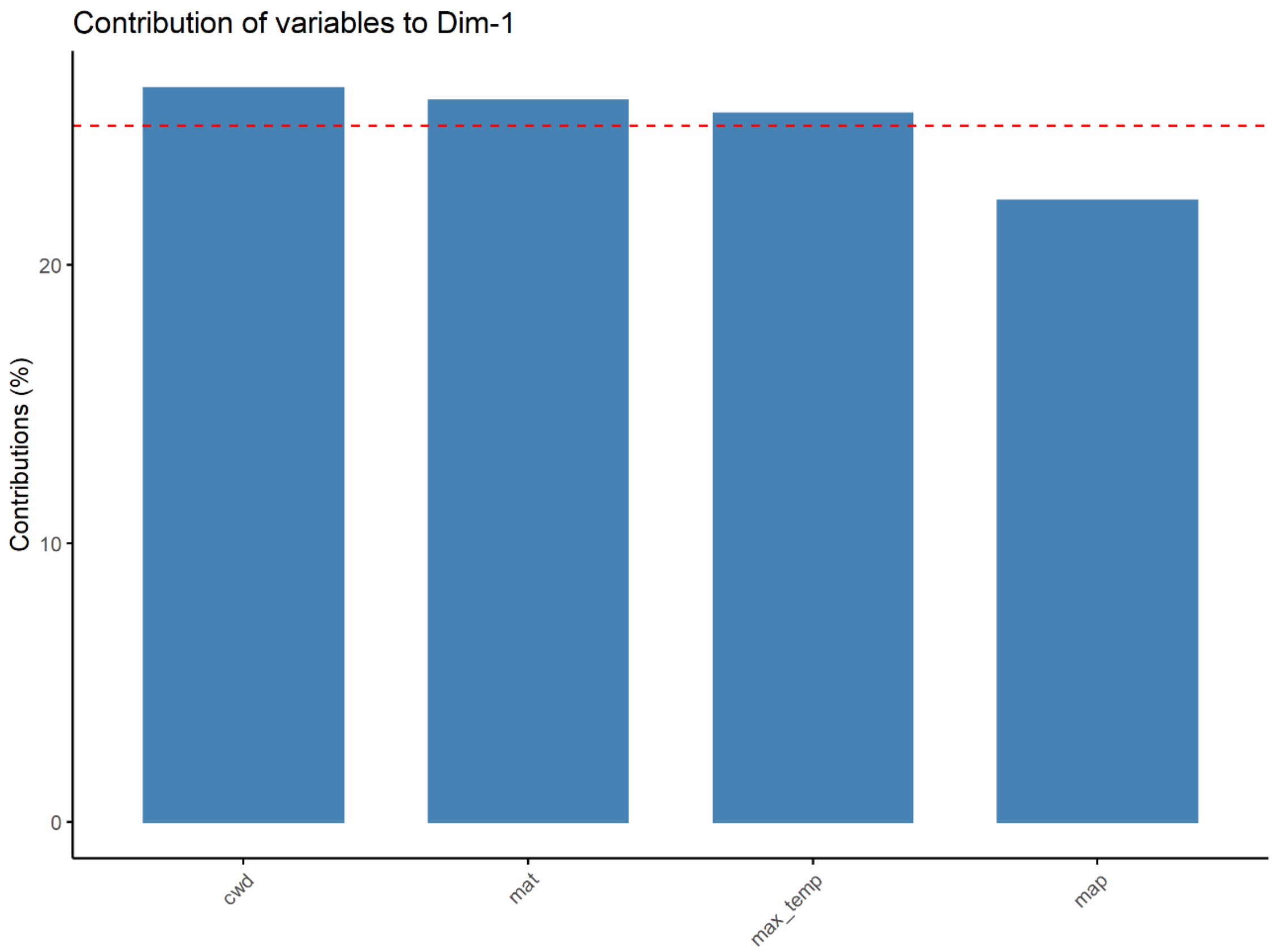

Figure S13. **Contribution Plot of Climate Variables at Census Level**. This contribution plot displays the contribution of four climate variables—Mean Annual Temperature (mat), Maximum Temperature (max_temp), Mean Annual Precipitation (map), and Climatic Water Deficit (cwd)—to the first principal component (PC1) on the y-axis. The analysis encompasses data aggregated over complete years within each interval (n = 163). Source data are provided as a Source Data file.

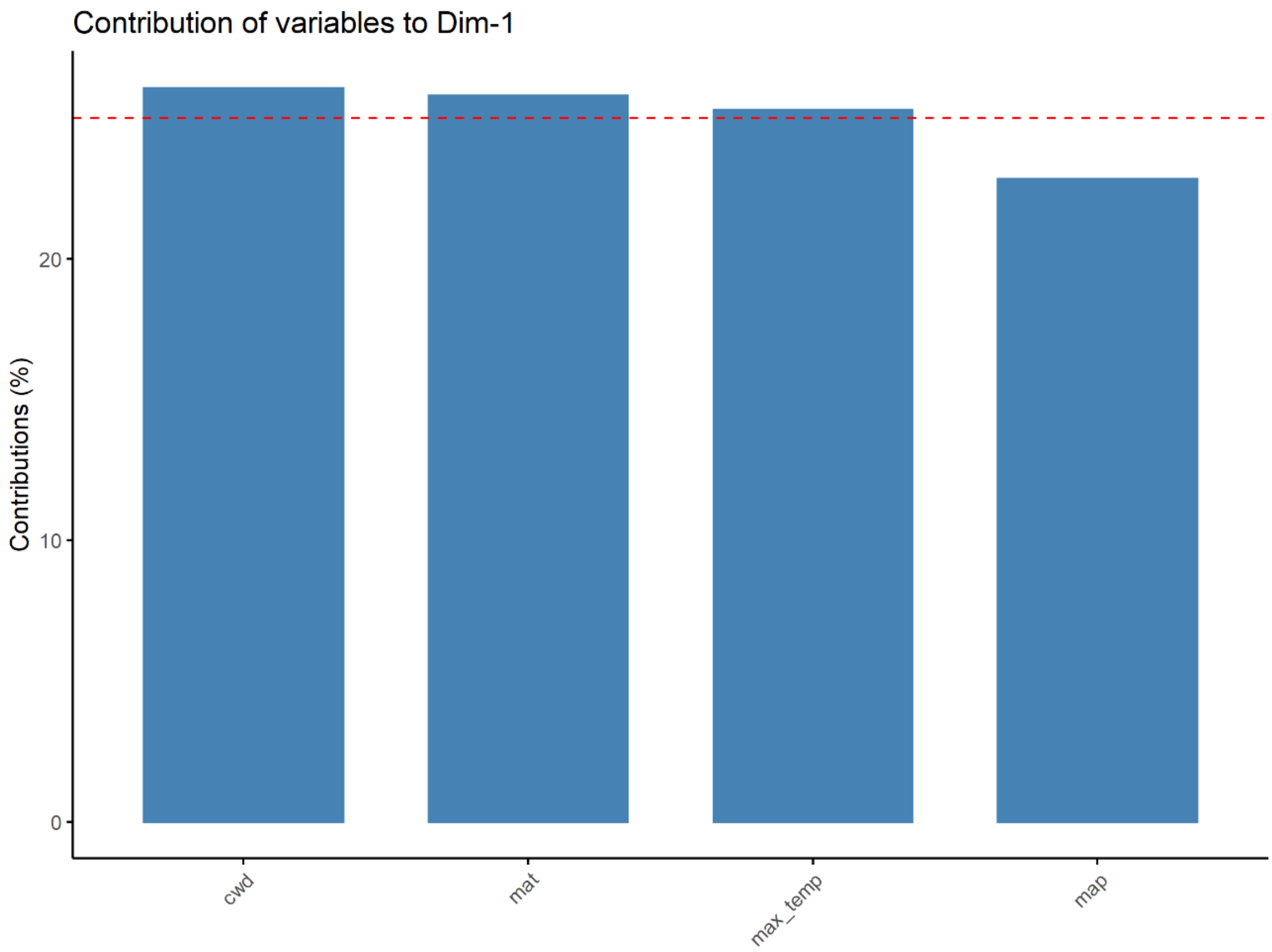

Figure S14. Contribution Plot of Climate Variables at Census Interval Level. This contribution plot displays the contribution of four climate variables—Mean Annual Temperature (mat), Maximum Temperature (max_temp), Mean Annual Precipitation (map), and Climatic Water Deficit (cwd)—to the first principal component (PC1) on the y-axis. The analysis encompasses data aggregated over complete years within each interval (n = 114). Source data are provided as a Source Data file.

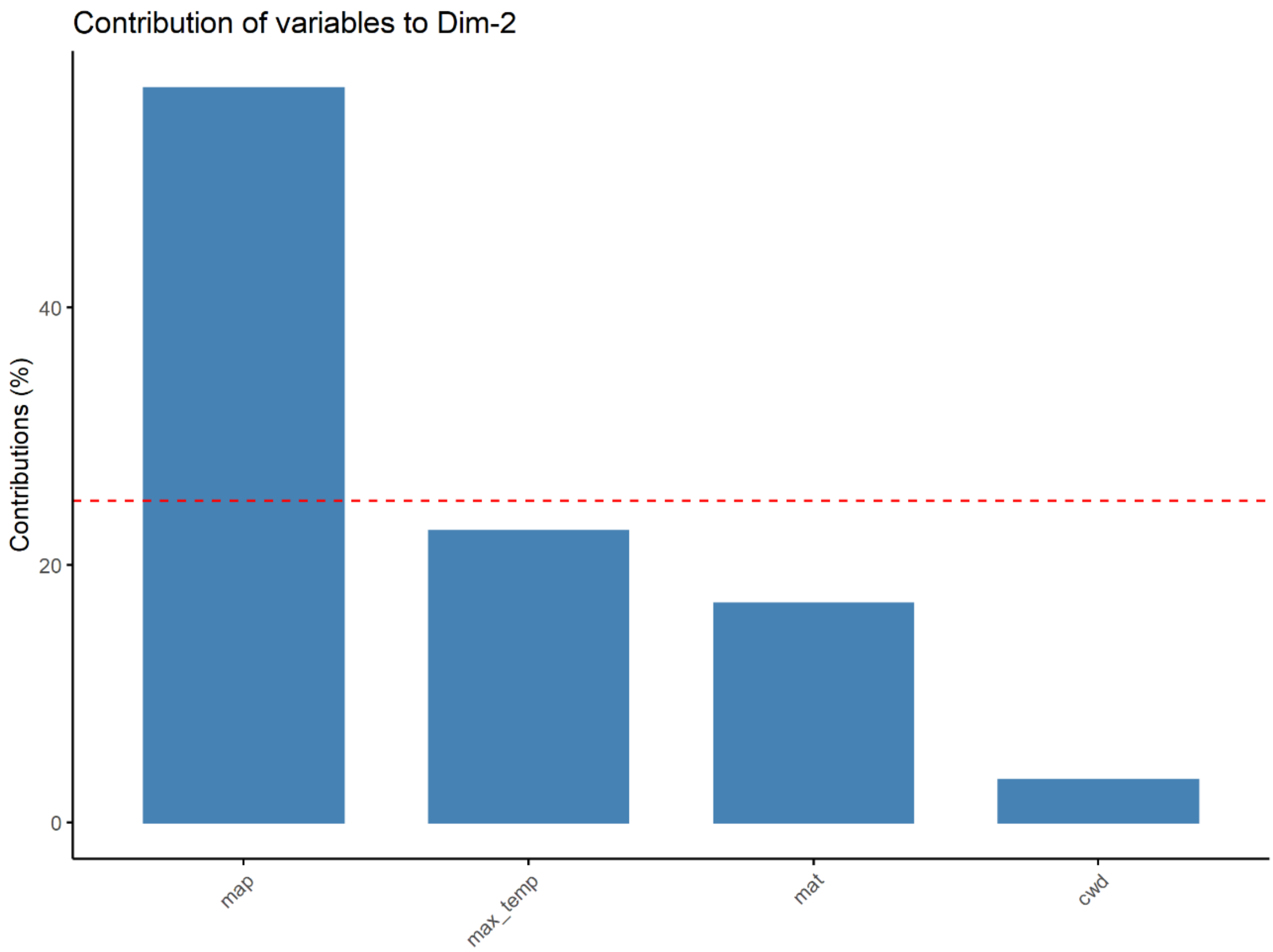

Figure S15. Contribution Plot of Climate Variables to PC2 at Census Level. This contribution plot displays the contribution of four climate variables—Mean Annual Temperature (mat), Maximum Temperature (max_temp), Mean Annual Precipitation (map), and Climatic Water Deficit (cwd)—to the second principal component (PC2) on the y-axis. The analysis encompasses data aggregated over complete years within each interval (n = 163). Source data are provided as a Source Data file.

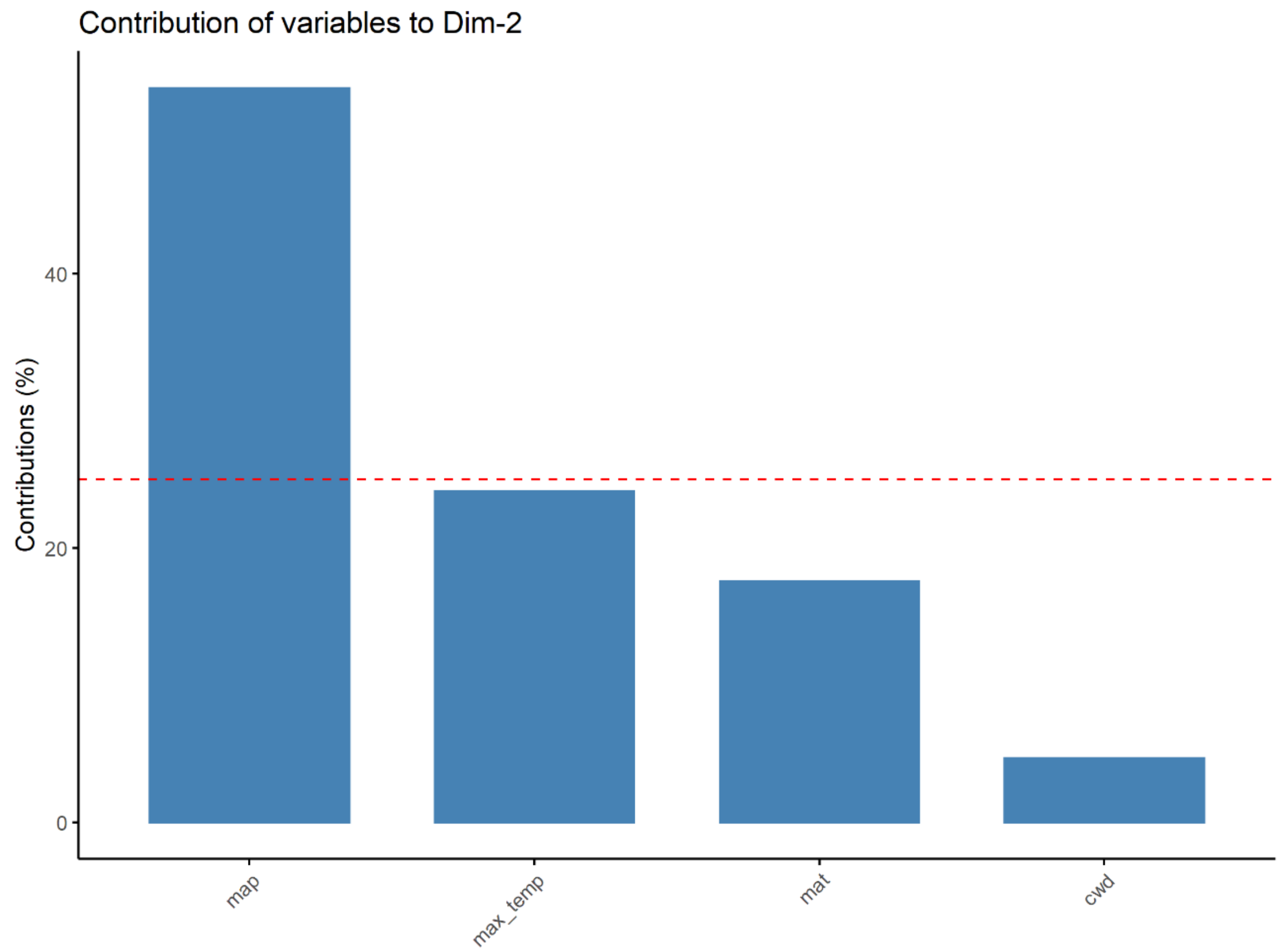

Figure S16. Contribution Plot of Climate Variables to PC2 at Census Interval Level. This contribution plot displays the contribution of four climate variables—Mean Annual Temperature (mat), Maximum Temperature (max_temp), Mean Annual Precipitation (map), and Climatic Water Deficit (cwd)—to the second principal component (PC2) on the y-axis. The analysis encompasses data aggregated over complete years within each interval (n = 114). Source data are provided as a Source Data file.

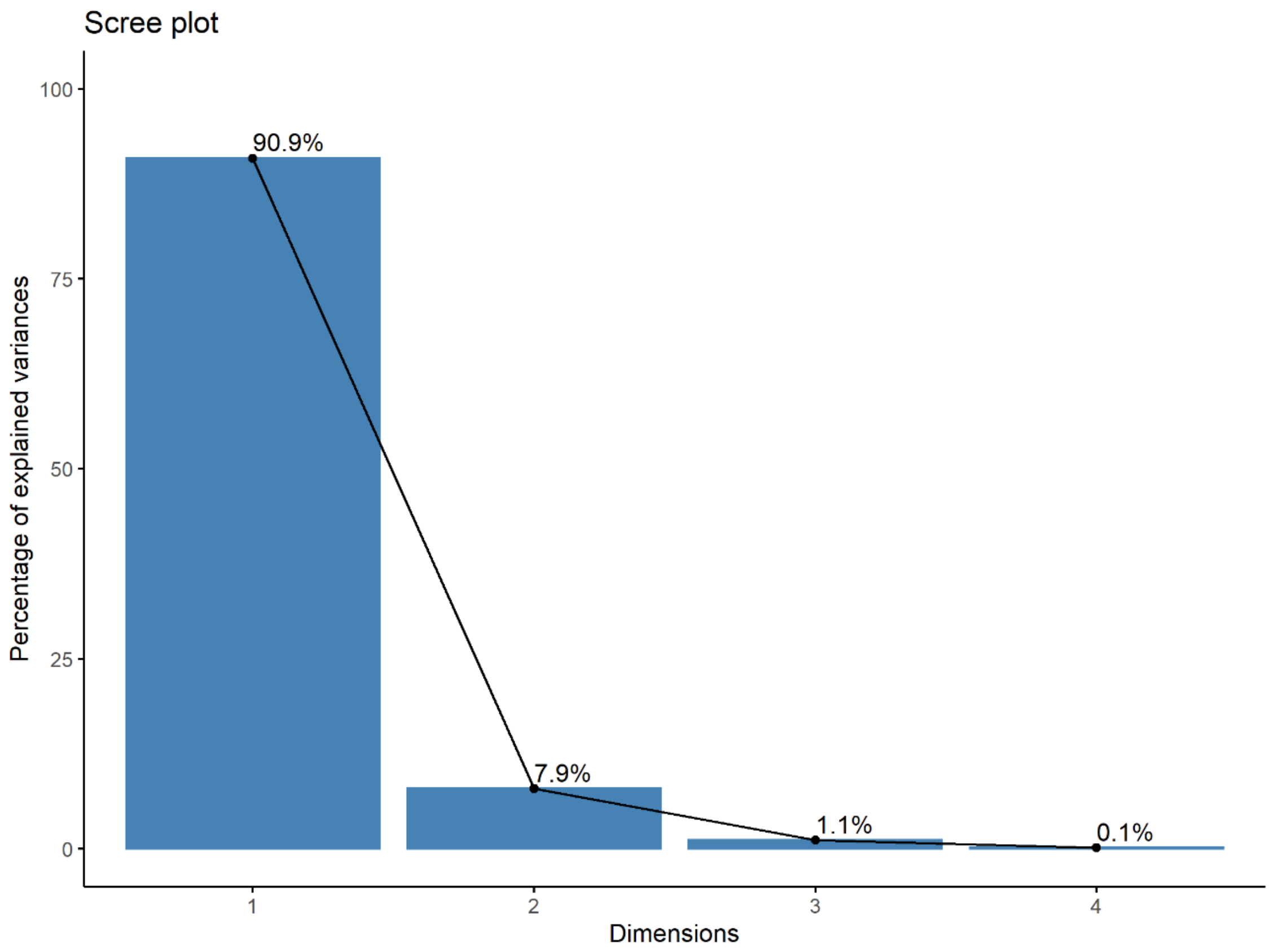

Figure S17. Scree Plot of Principal Components at Census Level. This scree plot shows the percentage of explained variance of the principal components derived from climate data at the census level. The analysis encompasses data aggregated over complete years within each interval (n = 163). Source data are provided as a Source Data file.

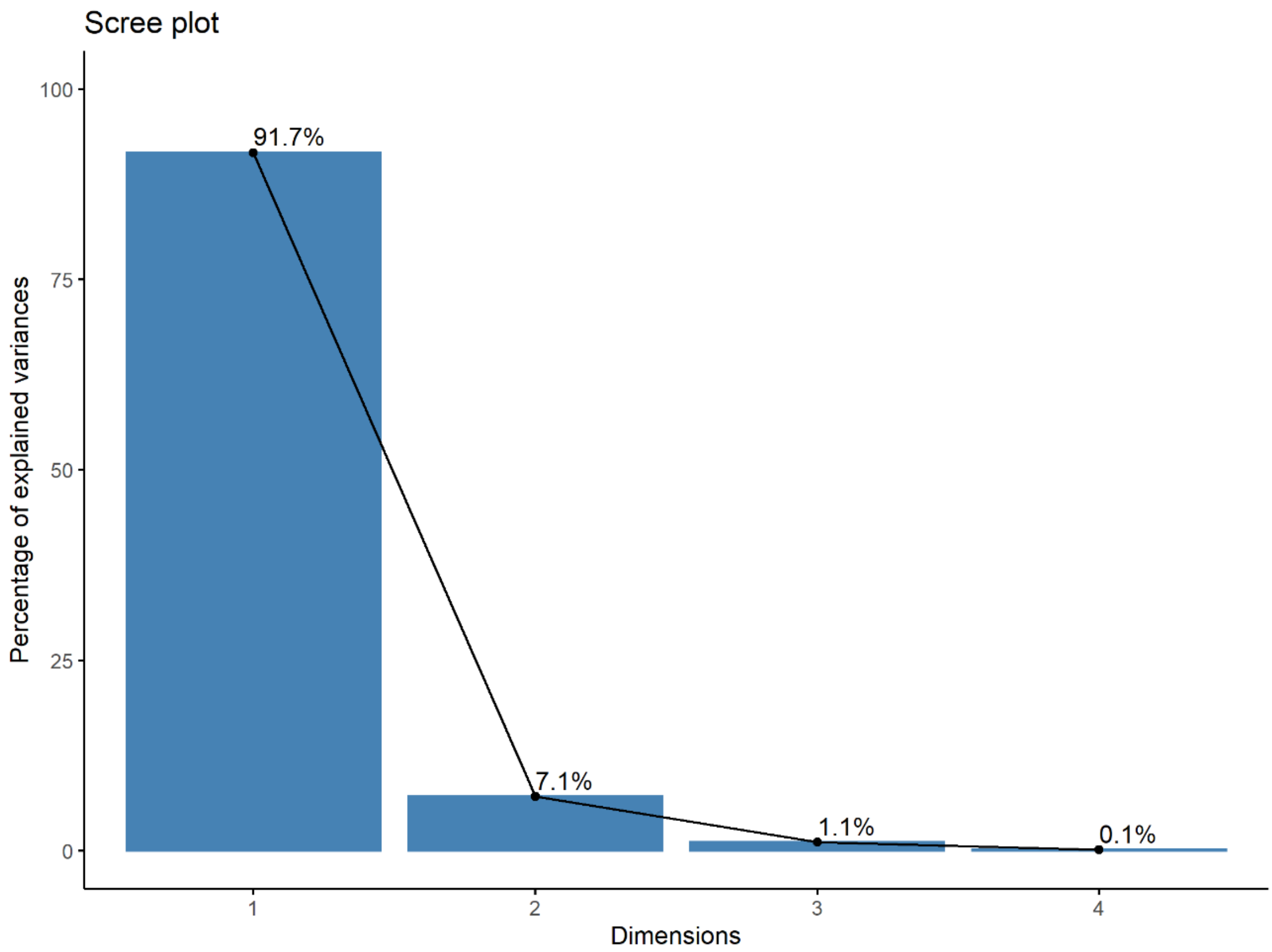

Figure S18. Scree Plot of Principal Components at Census Interval Level. This scree plot shows the percentage of explained variance of the principal components derived from climate data at the census interval level. The analysis encompasses data aggregated over complete years within each interval (n = 114). Source data are provided as a Source Data file.

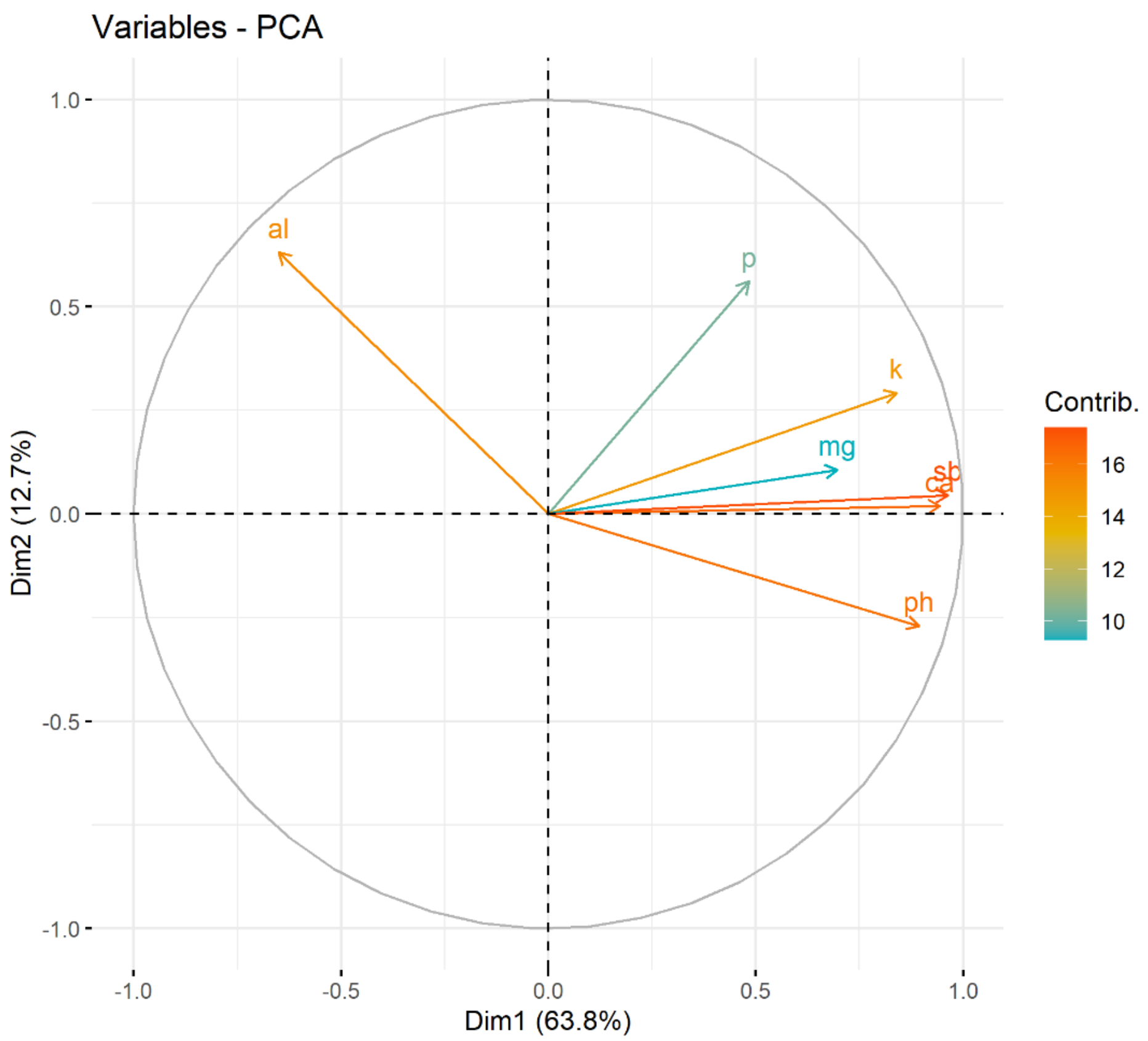

Figure S19. Principal Component Analysis (PCA) Biplot of Soil Variables (Dim1 vs Dim2) at Census Level. This PCA biplot visualizes the relationship among seven soil variables: pH (ph), Phosphorus (p), Potassium (k), Calcium (ca), Magnesium (mg), Aluminum (al), and Base Saturation (sb). The analysis encompasses data aggregated over complete years within each interval (n = 49). Source data are provided as a Source Data file.

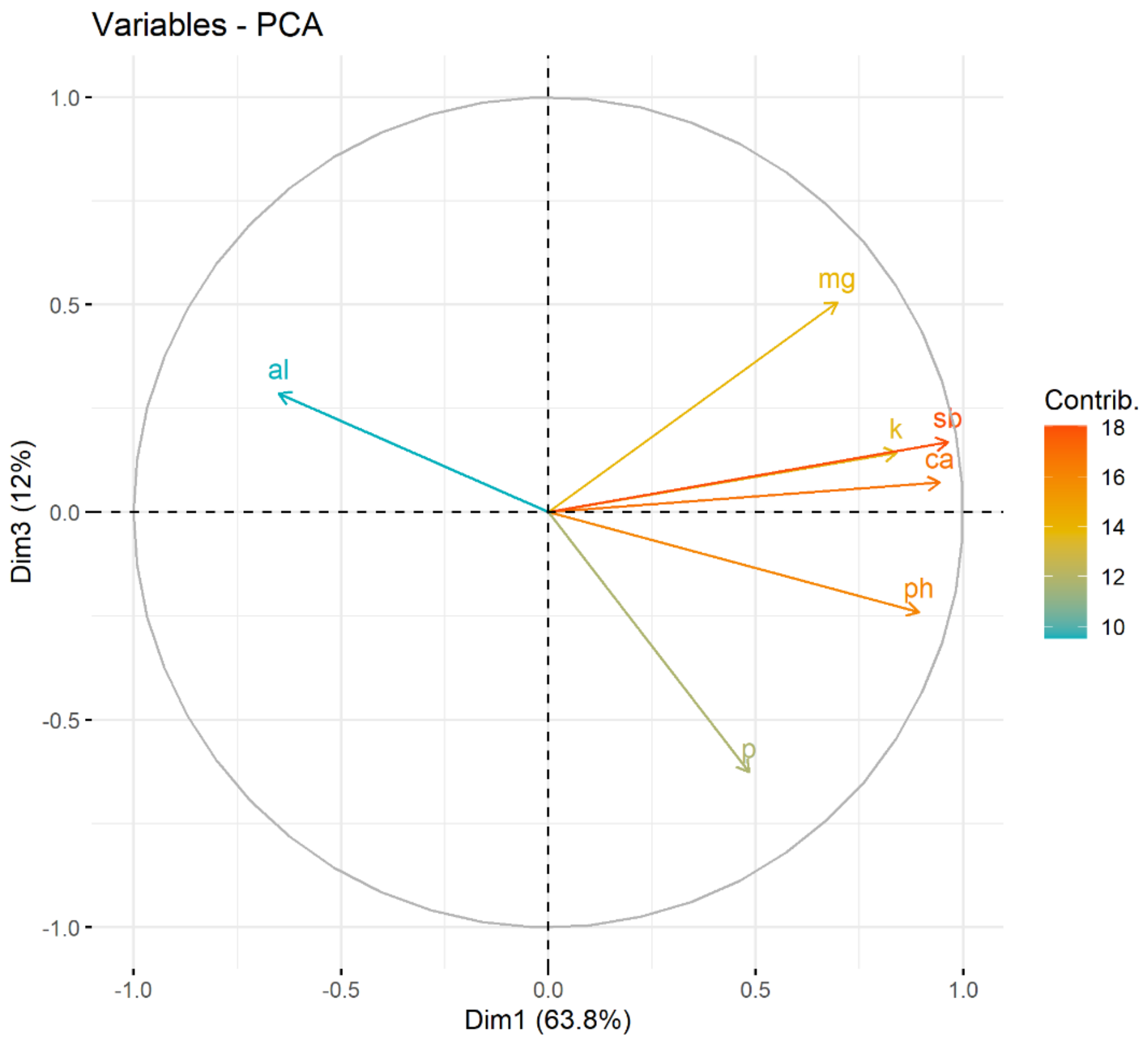

Figure S20. Principal Component Analysis (PCA) Biplot of Soil Variables (Dim1 vs Dim3) at Census Level. This PCA biplot visualizes the relationship among seven soil variables: pH (ph), Phosphorus (p), Potassium (k), Calcium (ca), Magnesium (mg), Aluminum (al), and Base Saturation (sb). The analysis encompasses data aggregated over complete years within each interval (n = 49). Source data are provided as a Source Data file.

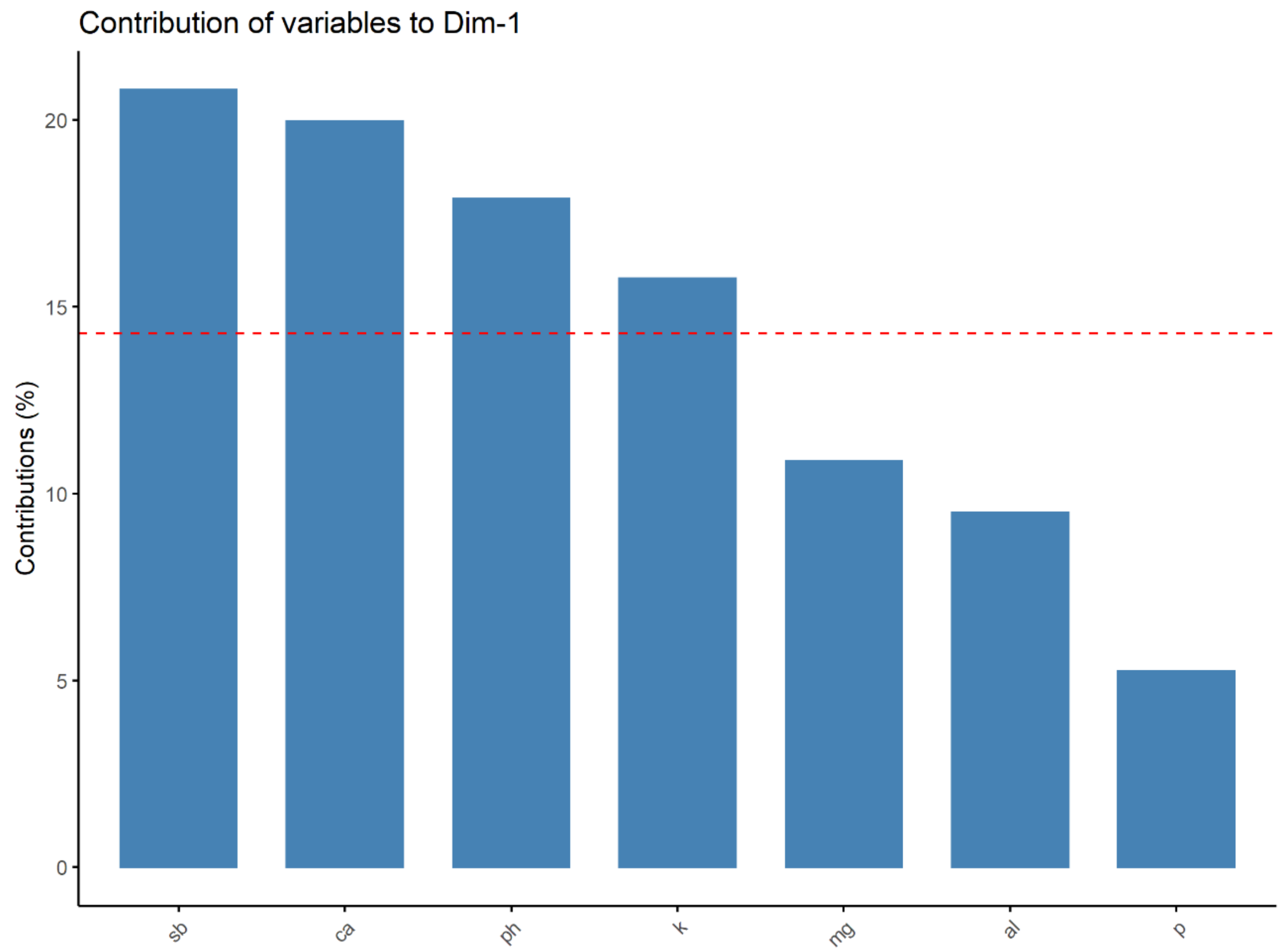

Figure S21. Contribution Plot of Soil Variables to PC1 at Census Level. This contribution plot displays the contribution of seven soil variables—pH (ph), Phosphorus (p), Potassium (k), Calcium (ca), Magnesium (mg), Aluminum (al), and Base Saturation (sb)—to the first principal component (PC1) on the y-axis. The analysis encompasses data aggregated over complete years within each interval (n = 49). Source data are provided as a Source Data file.

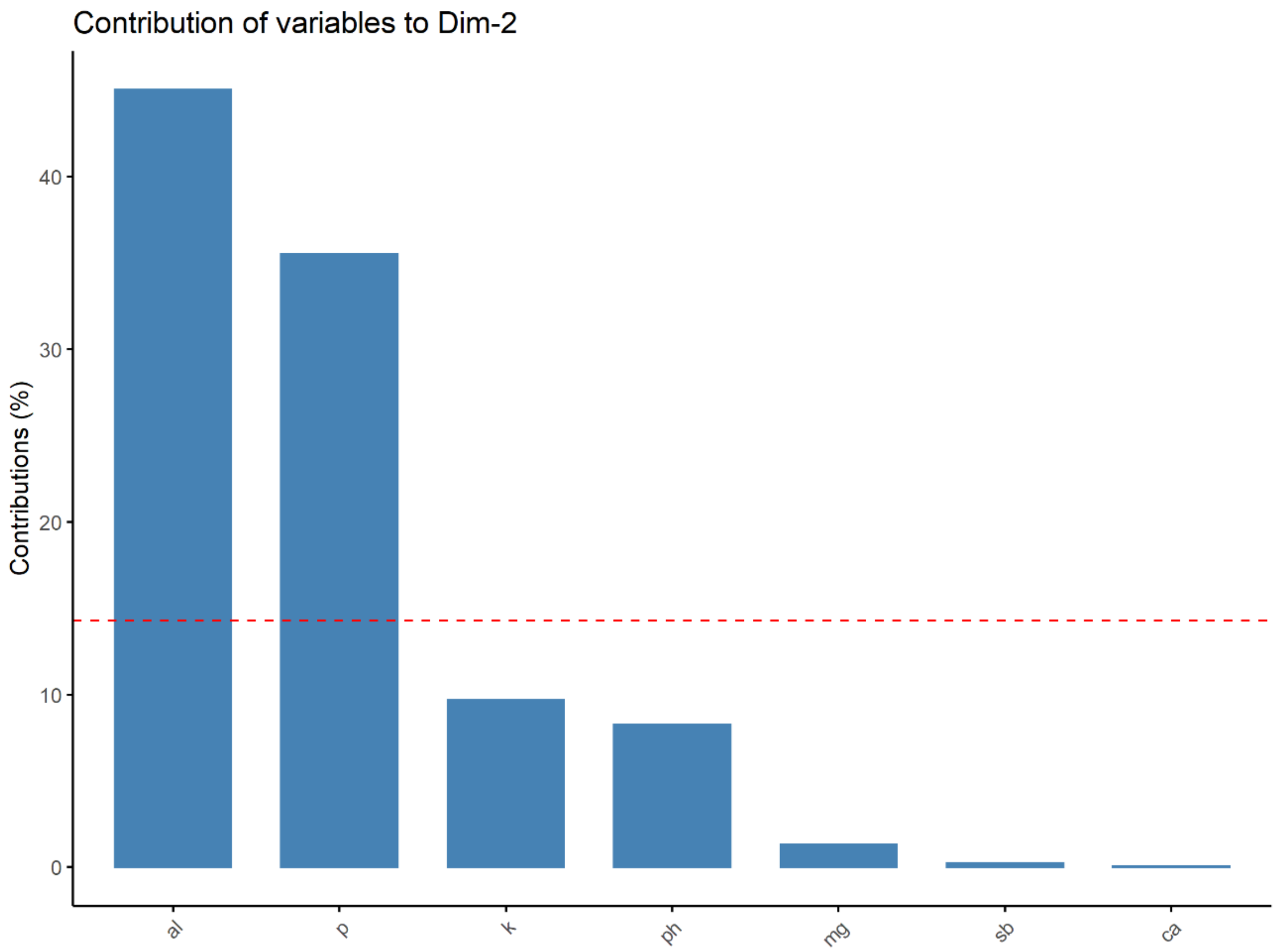

Figure S22. Contribution Plot of Soil Variables to PC2 at Census Level. This contribution plot displays the contribution of seven soil variables—pH (ph), Phosphorus (p), Potassium (k), Calcium (ca), Magnesium (mg), Aluminum (al), and Base Saturation (sb)—to the second principal component (PC2) on the y-axis. The analysis encompasses data aggregated over complete years within each interval (n = 49). Source data are provided as a Source Data file.

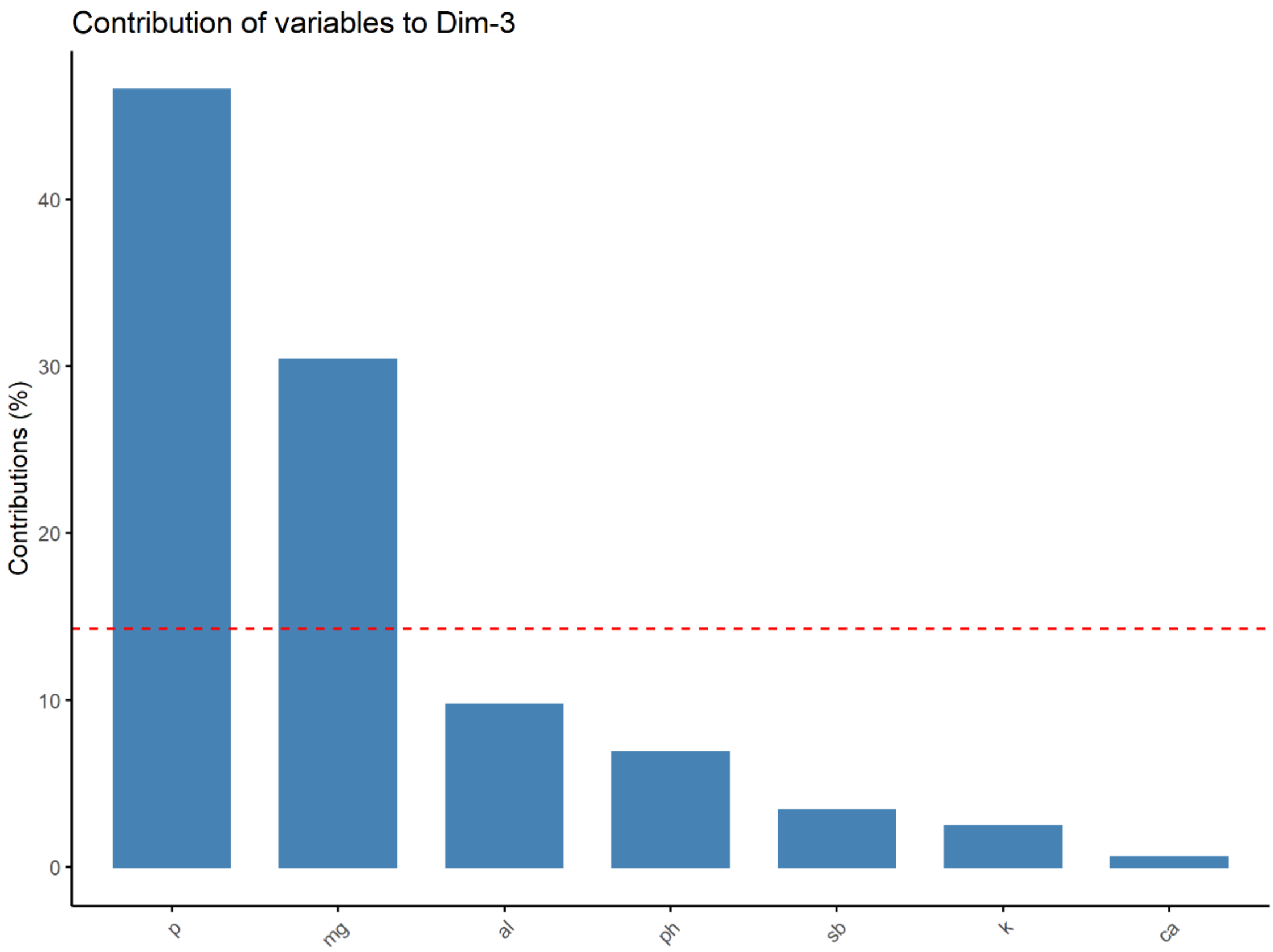

Figure S23. Contribution Plot of Soil Variables to PC3 at Census Level. This contribution plot displays the contribution of seven soil variables—pH (ph), Phosphorus (p), Potassium (k), Calcium (ca), Magnesium (mg), Aluminum (al), and Base Saturation (sb)—to the third principal component (PC3) on the y-axis. The analysis encompasses data aggregated over complete years within each interval (n = 49). Source data are provided as a Source Data file.

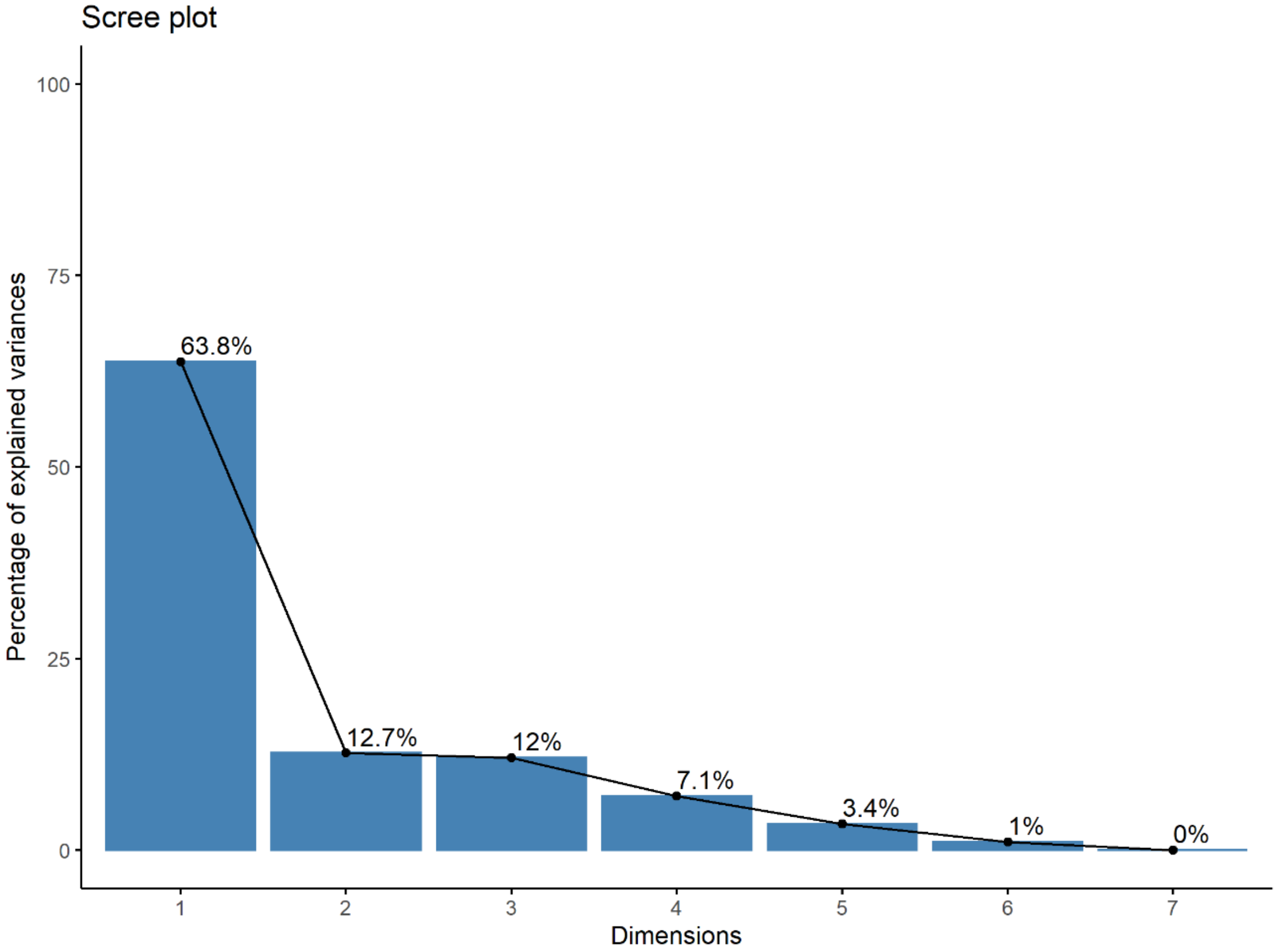

Figure S24. Scree Plot of Principal Components for Soil Variables at Census Level. This scree plot shows the percentage of explained variance of the principal components derived from soil data at the census level. The analysis encompasses data aggregated over complete years within each interval (n = 49). Source data are provided as a Source Data file.

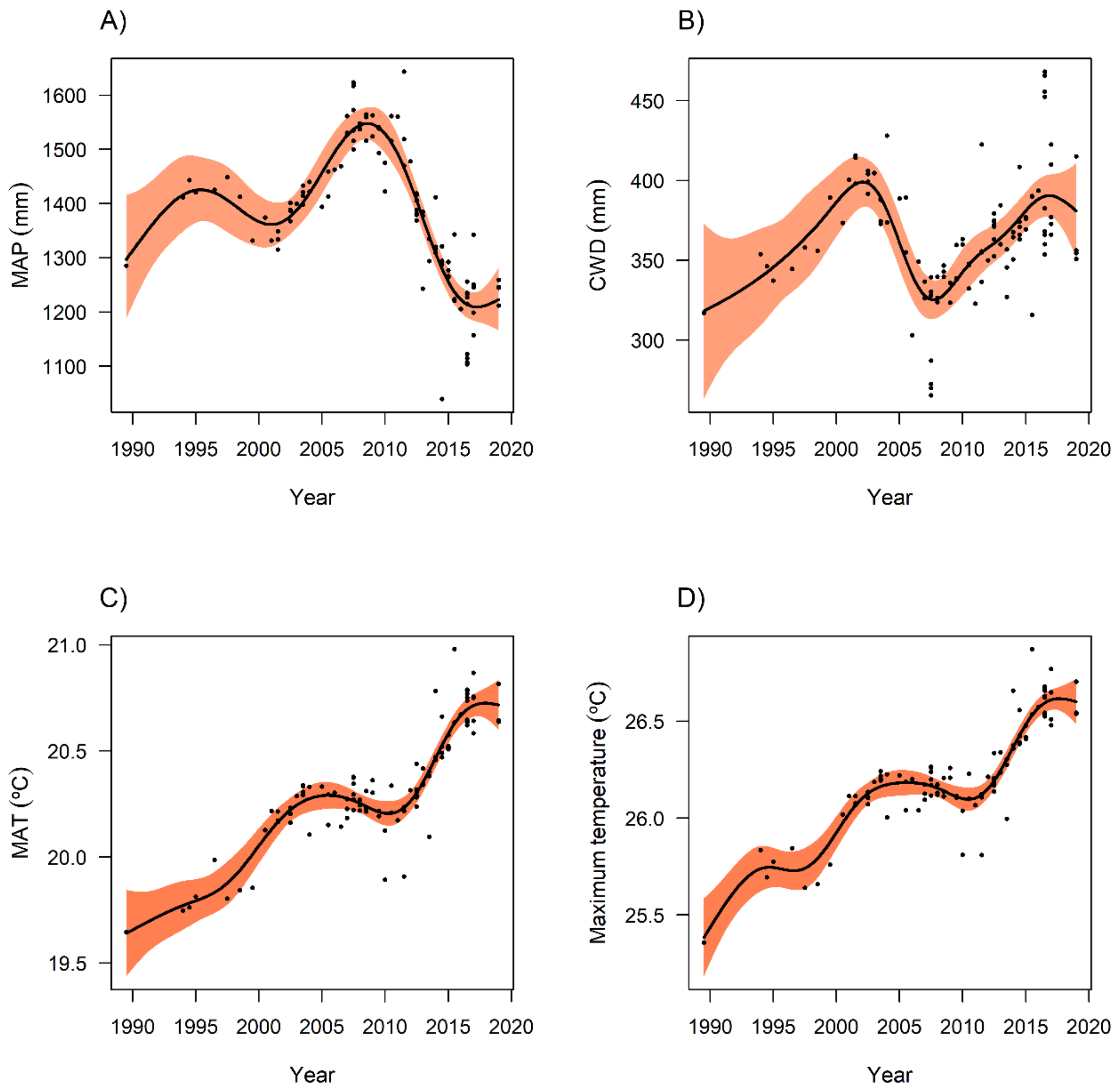

Figure S25. Long-term trends of A) mean annual precipitation (MAP), B) climatological water deficit (CWD), C) mean annual temperature (MAT) and D) maximum temperature. Curves are smooth splines fitted with generalized additive models (GAM). Points are partial residuals (after accounting for the random effect of sites, n = 49), corresponding to each census interval (n = 114). The shades are the 95% confidence bands of the smooths. Statistical significance of the smooth terms was assessed using two-sided F-tests. See table S1 for statistical details. Source data are provided as a Source Data file.

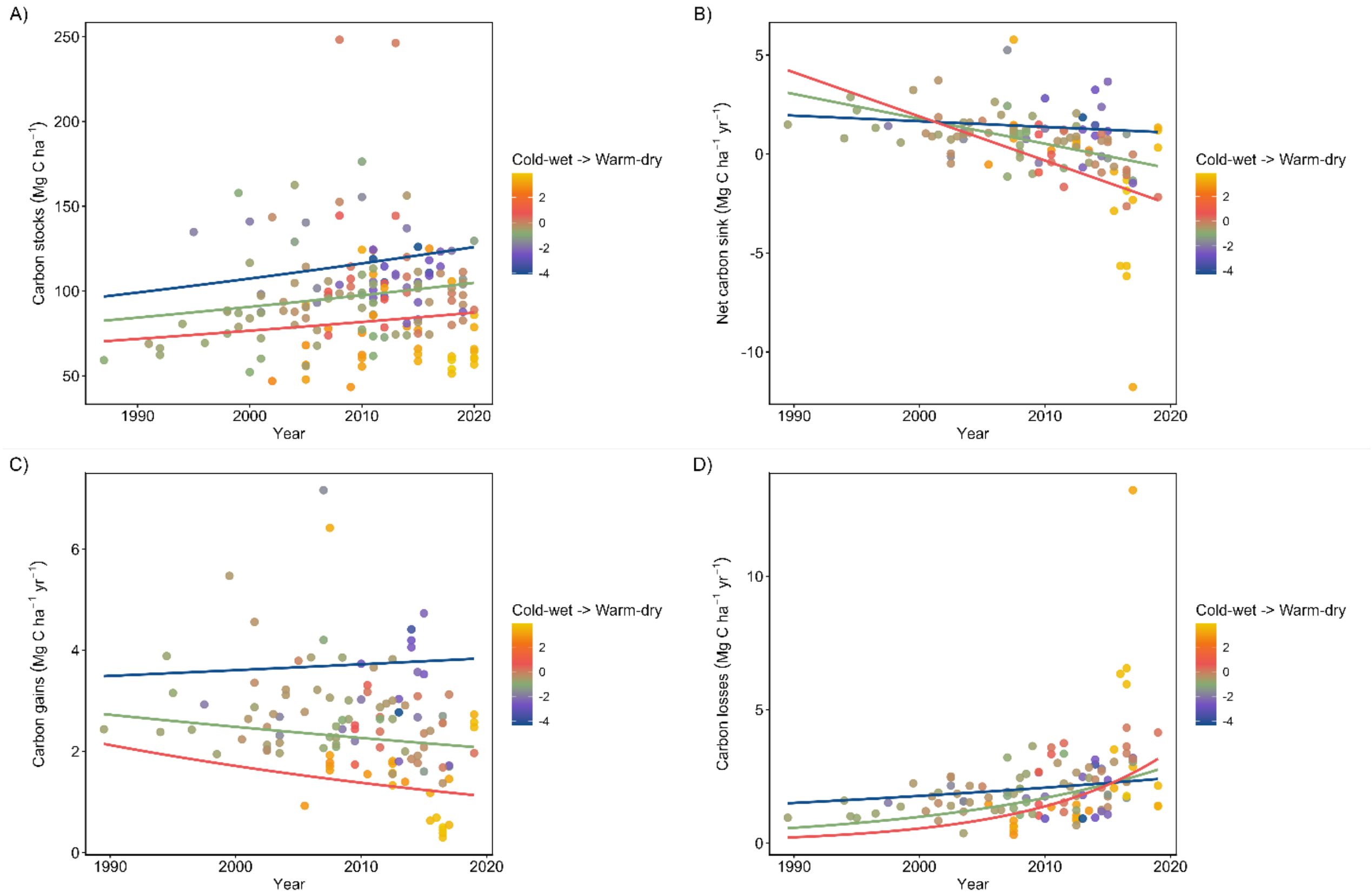

Figure S26. Long-term trends of A) carbon stocks (n = 163), B) net carbon sink (n = 114), C) carbon gains (n = 114), D) carbon losses (n = 114) on varying levels of climate PCA axis. Points are raw residuals and the curves were fitted with linear mixed-effects models (LMM, including a random effect of site). The statistical significance was assessed using two-sided asymptotic z-tests. See tables S2-S9 for statistical details. Source data are provided as a Source Data file.

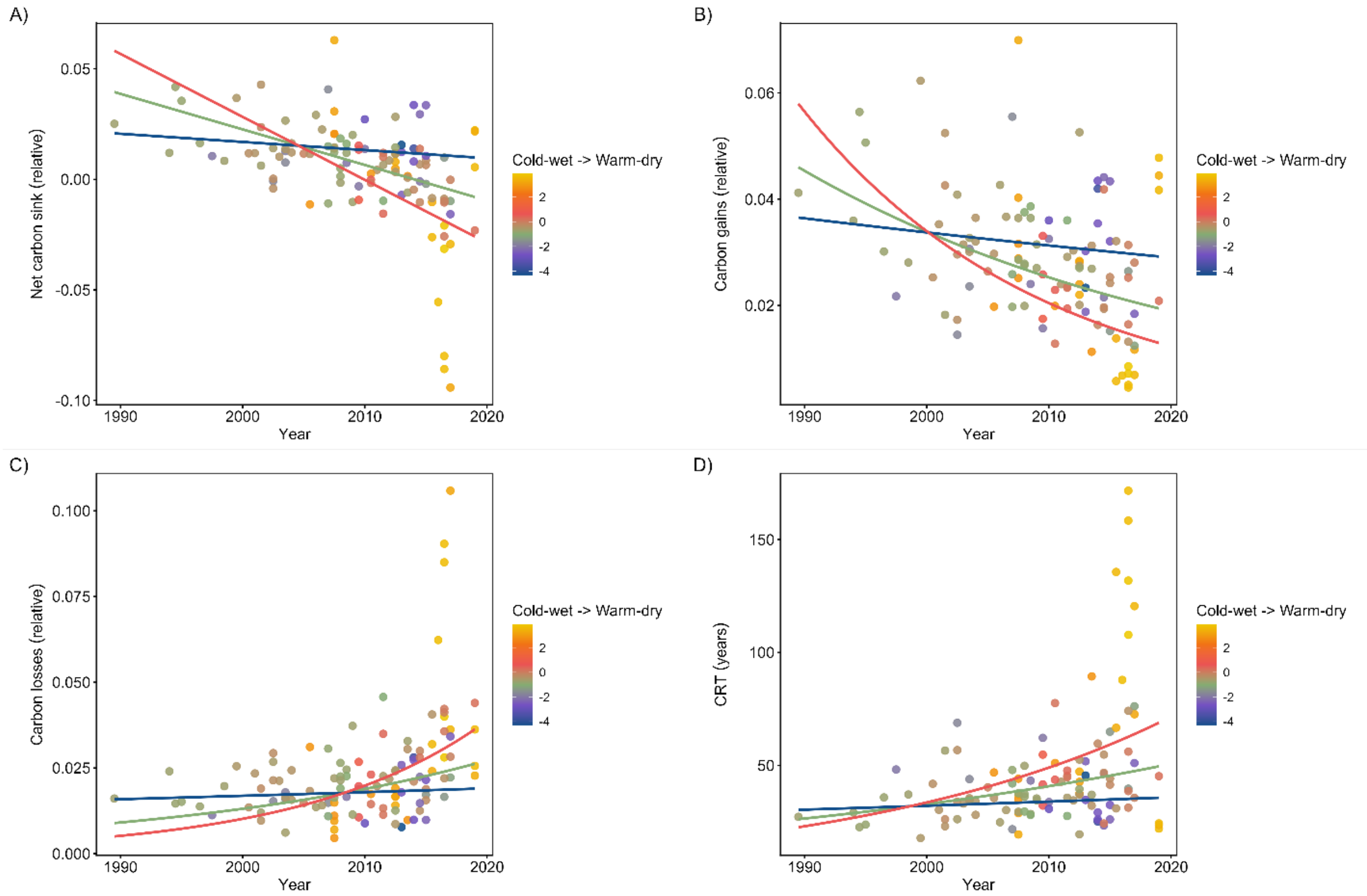

Figure S27. Long-term trends of A) relative net carbon sink, B) relative carbon gains, C) relative carbon losses, D) carbon residence time (CRT) on varying levels of climate PCA axis. Points (n = 114) are raw residuals and the curves were fitted with linear mixed-effects models (LMM, including a random effect of site). The statistical significance was assessed using two-sided asymptotic z-tests. See tables S2-S9 for statistical details. Source data are provided as a Source Data file.

Supplementary tables

Table S1. Responses variables as a function of time (year) from GAMs. Where: edf, estimated degrees of freedom; Ref df, reference degrees of freedom; F, F statistic; P-value, p value from a two-sided F test. Source data are provided as a Source Data file.

Response	edf	Ref df	F	P-value
Carbon stocks	5.267173	6.368528	22.785825	0.000000
Net carbon sink	2.879636	3.638888	9.050494	0.000008
Net carbon sink (relative)	2.759264	3.474413	11.043627	0.000001
Carbon gains	2.253499	2.805165	5.369606	0.003970
Carbon gains (relative)	1.385291	1.663393	13.424628	0.000032
Carbon losses	2.562523	3.209600	8.765392	0.000024
Carbon losses (relative)	3.423694	4.289889	7.173547	0.000027
CRT	1.000279	1.000541	19.951173	0.000023
MAP	7.150681	8.131909	40.235877	0.000000
CWD	6.902408	7.935555	10.791391	0.000000
MAT	7.123157	8.115427	50.651630	0.000000
Max. temp	7.624166	8.477826	52.576264	0.000000
Taxonomic diversity (Shannon)	2.643250	3.329096	11.497351	0.000001
Phylogenetic diversity (PD)	1.000236	1.000471	17.589336	0.000055
WD	3.828963	4.756431	2.909544	0.019216
WD dispersion	2.968709	3.725949	5.748158	0.000463
SLA	2.774617	3.489652	3.666618	0.011914
SLA dispersion	1.000104	1.000208	6.309056	0.013407

Table S2. Multimodel inference results of Carbon stocks; n = 163. Estimate, averaged model coefficient; Std. Error, standard error of the estimate; Adjusted SE, adjusted standard error; z value, test statistic; Pr(>|z|), p value from a two-sided asymptotic z test; CI 95%, 95% confidence interval of the estimates. The models were fitted with the response variable in the log-scale and centred and scaled predictors. Source data are provided as a Source Data file.

Carbon stocks (Mg C ha-1) $R^2_{marginal}$ = 31%						
Predictor	Estimate	Std. Error	Adjusted SE	z value	Pr(>|z|)	CI 95%
Intercept	4.579634	0.037493	0.037800	121.155874	0.000000	[4.506, 4.653]
SLA	0.049364	0.033407	0.033668	1.466203	0.142593	[-0.016, 0.115]
WD disp	-0.030525	0.036496	0.036779	0.829952	0.406566	[-0.102, 0.041]
Aluminum - phosphorus	-0.047013	0.040370	0.040684	1.155567	0.247858	[-0.126, 0.032]
Phosphorus -> magnesium	-0.097023	0.037493	0.037798	2.566863	0.010262	[-0.171, -0.024]
Site area	-0.012407	0.042098	0.042431	0.292398	0.769982	[-0.095, 0.070]
Soil fertility	0.041675	0.054518	0.054926	0.758753	0.448000	[-0.065, 0.149]
Cold-wet -> Warm-dry	-0.176131	0.045683	0.046032	3.826294	0.000130	[-0.266, -0.087]
Year	0.050539	0.007585	0.007640	6.614698	0.000000	[0.036, 0.065]
SLA:Year	-0.013200	0.007203	0.007259	1.818468	0.068993	[-0.027, 0.001]
WD disp:Year	-0.039612	0.008057	0.008118	4.879502	0.000001	[-0.055, -0.024]
Phylogenetic diversity	0.013409	0.021882	0.022059	0.607889	0.543261	[-0.029, 0.056]

Carbon stocks (Mg C ha-1) $R^2_{marginal}$ = 31%						
Predictor	Estimate	Std. Error	Adjusted SE	z value	Pr(>|z|)	CI 95%
Phylogenetic diversity:Year	0.014041	0.006755	0.006809	2.061996	0.039208	[0.001, 0.027]
Taxonomic diversity	0.049461	0.043017	0.043351	1.140952	0.253890	[-0.035, 0.134]
Taxonomic diversity:Year	-0.012174	0.007641	0.007696	1.581908	0.113671	[-0.027, 0.003]
SLA disp	-0.018419	0.030425	0.030650	0.600970	0.547860	[-0.078, 0.041]
Cold-wet -> Warm-dry:Year	-0.005112	0.013017	0.013091	0.390479	0.696183	[-0.031, 0.020]
WD	-0.001735	0.037769	0.038055	0.045594	0.963634	[-0.076, 0.072]
SLA disp:Year	0.010462	0.009961	0.010030	1.043075	0.296914	[-0.009, 0.030]

Table S3. Multimodel inference results of the net carbon sink; n = 114. Estimate, averaged model coefficient; Std. Error, standard error of the estimate; Adjusted SE, adjusted standard error; z value, test statistic; Pr(>|z|), p value from a two-sided asymptotic z test; CI 95%, 95% confidence interval of the estimates. The models were fitted with centred and scaled predictors. Source data are provided as a Source Data file.

Net carbon sink (Mg C ha-1 yr-1) R^2 marginal = 33%						
Predictor	Estimate	Std. Error	Adjusted SE	z value	Pr(>|z|)	CI 95%
Intercept	0.556241	0.145949	0.147695	3.766138	0.000166	[0.270, 0.842]
SLA	-0.393825	0.206672	0.208607	1.887879	0.059042	[-0.799, 0.011]
SLA disp	0.327199	0.244644	0.246813	1.325697	0.184940	[-0.152, 0.807]
WD disp	-0.461510	0.246593	0.249044	1.853129	0.063864	[-0.945, 0.022]
Aluminum - phosphorus	-0.301833	0.197566	0.199640	1.511888	0.130563	[-0.689, 0.085]
Phosphorus -> magnesium	-0.110359	0.193036	0.195244	0.565237	0.571913	[-0.489, 0.268]
Site area	0.021427	0.190028	0.191892	0.111661	0.911092	[-0.351, 0.394]
Soil fertility	0.692060	0.282155	0.285368	2.425154	0.015302	[0.139, 1.245]
Cold-wet -> Warm-dry	-0.822855	0.437181	0.440659	1.867328	0.061856	[-1.680, 0.034]
Year	-0.774901	0.161989	0.163893	4.728095	0.000002	[-1.092, -0.457]
Cold-wet -> Warm-dry:Year	-0.598828	0.227349	0.229909	2.604625	0.009198	[-1.044, -0.153]
WD	0.389411	0.183573	0.185518	2.099051	0.035812	[0.030, 0.749]
Phylogenetic diversity	0.234202	0.177669	0.179571	1.304234	0.192154	[-0.114, 0.582]

Net carbon sink (Mg C ha-1 yr-1) R^2 marginal = 33%						
Predictor	Estimate	Std. Error	Adjusted SE	z value	Pr(>|z|)	CI 95%
WD disp:Year	-0.370327	0.235098	0.237124	1.561742	0.118349	[-0.831, 0.090]
SLA disp:Year	0.223720	0.174510	0.176505	1.267499	0.204977	[-0.118, 0.566]
Taxonomic diversity	-0.113984	0.208701	0.211066	0.540040	0.589169	[-0.523, 0.295]
SLA:Year	0.012501	0.168626	0.170536	0.073307	0.941562	[-0.318, 0.343]
WD:Year	0.023567	0.215661	0.218240	0.107985	0.914008	[-0.399, 0.446]
Phylogenetic diversity:Year	-0.007159	0.168455	0.170505	0.041985	0.966511	[-0.337, 0.323]

Table S4. Multimodel inference results of the net carbon sink relativized by carbon stocks; n = 114. Estimate, averaged model coefficient; Std. Error, standard error of the estimate; Adjusted SE, adjusted standard error; z value, test statistic; Pr(>|z|), p value from a two-sided asymptotic z test; CI 95%, 95% confidence interval of the estimates. The models were fitted with centred and scaled predictors. Source data are provided as a Source Data file.

Net carbon sink (relative) R^2 marginal = 35%						
Predictor	Estimate	Std. Error	Adjusted SE	z value	Pr(>|z|)	CI 95%
Intercept	0.006952	0.001554	0.001572	4.420970	0.000010	[0.004, 0.010]
SLA	-0.004426	0.002184	0.002205	2.007431	0.044704	[-0.009, -0.000]
SLA disp	0.003312	0.002666	0.002688	1.232307	0.217835	[-0.002, 0.009]
WD disp	-0.005389	0.002751	0.002777	1.940600	0.052307	[-0.011, 0.000]
Aluminum - phosphorus	-0.002660	0.002096	0.002119	1.255337	0.209357	[-0.007, 0.001]
Phosphorus -> magnesium	-0.001487	0.002058	0.002082	0.714577	0.474870	[-0.006, 0.003]
Site area	0.000891	0.001976	0.001996	0.446147	0.655491	[-0.003, 0.005]
Soil fertility	0.006164	0.003015	0.003049	2.021576	0.043220	[0.000, 0.012]
Cold-wet -> Warm-dry	-0.006390	0.004802	0.004839	1.320680	0.186608	[-0.016, 0.003]
Year	-0.009930	0.001711	0.001731	5.735809	0.000000	[-0.013, -0.007]
Cold-wet -> Warm-dry:Year	-0.007654	0.002361	0.002388	3.204509	0.001353	[-0.012, -0.003]
WD	0.004547	0.002011	0.002032	2.237730	0.025239	[0.001, 0.008]

Net carbon sink (relative) R^2 marginal = 35%						
Predictor	Estimate	Std. Error	Adjusted SE	z value	Pr(>|z|)	CI 95%
SLA disp:Year	0.002913	0.001884	0.001904	1.529500	0.126140	[-0.001, 0.007]
Phylogenetic diversity	0.001633	0.001894	0.001915	0.853021	0.393647	[-0.002, 0.005]
Taxonomic diversity	-0.001523	0.002269	0.002294	0.664017	0.506679	[-0.006, 0.003]
SLA:Year	0.000535	0.001802	0.001822	0.293677	0.769004	[-0.003, 0.004]
WD:Year	-0.001694	0.002374	0.002401	0.705461	0.480523	[-0.006, 0.003]
WD disp:Year	0.000230	0.002395	0.002423	0.094885	0.924406	[-0.004, 0.005]

Table S5. Multimodel inference results of Carbon gains; n = 114. Estimate, averaged model coefficient; Std. Error, standard error of the estimate; Adjusted SE, adjusted standard error; z value, test statistic; Pr(>|z|), p value from a two-sided asymptotic z test; CI 95%, 95% confidence interval of the estimates. The models were fitted with the response variable in the log-scale and centred and scaled predictors. Source data are provided as a Source Data file.

Carbon gains (Mg C ha-1 yr-1) R^2 marginal = 47%						
Predictor	Estimate	Std. Error	Adjusted SE	z value	Pr(>|z|)	CI 95%
Intercept	0.821191	0.032009	0.032379	25.361439	0.000000	[0.758, 0.884]
WD	0.109238	0.043792	0.044221	2.470261	0.013501	[0.023, 0.195]
Phylogenetic diversity	0.099530	0.036639	0.037061	2.685593	0.007240	[0.028, 0.171]
Aluminum - phosphorus	-0.173479	0.039974	0.040428	4.291095	0.000018	[-0.252, -0.095]
Phosphorus -> magnesium	-0.090452	0.041673	0.042173	2.144802	0.031969	[-0.172, -0.009]
Site area	-0.040149	0.037374	0.037800	1.062143	0.288171	[-0.113, 0.033]
Soil fertility	0.178459	0.060303	0.061019	2.924665	0.003448	[0.060, 0.297]
Cold-wet -> Warm-dry	-0.492510	0.081006	0.081834	6.018424	0.000000	[-0.651, -0.334]
Year	-0.056749	0.036048	0.036460	1.556456	0.119600	[-0.127, 0.014]
WD:Year	-0.076396	0.044267	0.044788	1.705734	0.088058	[-0.163, 0.010]
Cold-wet -> Warm-dry:Year	-0.076418	0.055270	0.055829	1.368788	0.171066	[-0.185, 0.032]
SLA	-0.043193	0.037424	0.037873	1.140483	0.254085	[-0.117, 0.030]

Carbon gains (Mg C ha-1 yr-1) R^2 marginal = 47%						
Predictor	Estimate	Std. Error	Adjusted SE	z value	Pr(>|z|)	CI 95%
WD disp	-0.043629	0.057118	0.057748	0.755505	0.449946	[-0.156, 0.068]
Taxonomic diversity	-0.020075	0.043485	0.044003	0.456222	0.648230	[-0.105, 0.065]
Phylogenetic diversity:Year	-0.011679	0.037956	0.038404	0.304098	0.761053	[-0.086, 0.063]
SLA disp	-0.013412	0.044611	0.045116	0.297270	0.766260	[-0.101, 0.074]
WD disp:Year	0.082075	0.048617	0.049213	1.667766	0.095362	[-0.013, 0.177]

Table S6. Multimodel inference results of the carbon gains relativized by carbon stocks; n = 114. Estimate, averaged model coefficient; Std. Error, standard error of the estimate; Adjusted SE, adjusted standard error; z value, test statistic; Pr(>|z|), p value from a two-sided asymptotic z test; CI 95%, 95% confidence interval of the estimates. The models were fitted with the response variable in the log-scale and centred and scaled predictors. Source data are provided as a Source Data file.

Carbon gains (relative) R^2 marginal = 39%						
Predictor	Estimate	Std. Error	Adjusted SE	z value	Pr(>|z|)	CI 95%
Intercept	-3.669469	0.038732	0.039188	93.636380	0.000000	[-3.745, -3.594]
SLA	-0.149916	0.052763	0.053247	2.815463	0.004871	[-0.253, -0.047]
WD	0.121441	0.048721	0.049206	2.467997	0.013587	[0.026, 0.217]
Phylogenetic diversity	0.079824	0.047747	0.048221	1.655357	0.097852	[-0.014, 0.173]
SLA disp	0.095463	0.059678	0.060302	1.583076	0.113404	[-0.022, 0.212]
Aluminum - phosphorus	-0.124752	0.049131	0.049691	2.510554	0.012054	[-0.221, -0.028]
Phosphorus -> magnesium	-0.008538	0.048665	0.049227	0.173436	0.862309	[-0.104, 0.087]
Site area	-0.002034	0.049107	0.049611	0.041001	0.967296	[-0.098, 0.094]
Soil fertility	0.137643	0.070570	0.071400	1.927775	0.053883	[-0.001, 0.276]
Cold-wet -> Warm-dry	-0.206914	0.120210	0.121043	1.709429	0.087372	[-0.443, 0.029]
Year	-0.180305	0.038936	0.039374	4.579307	0.000005	[-0.257, -0.104]
Cold-wet -> Warm-dry:Year	-0.133545	0.052664	0.053269	2.507016	0.012176	[-0.237, -0.030]
WD disp	-0.136724	0.069834	0.070454	1.940613	0.052305	[-0.274, 0.000]

<table>
<tr><th colspan="7">Carbon gains (relative) R^2 marginal = 39%</th></tr>
<tr><th>Predictor</th><th>Estimate</th><th>Std. Error</th><th>Adjusted SE</th><th>z value</th><th>Pr(>|z|)</th><th>CI 95%</th></tr>
<tr><td>SLA:Year</td><td>0.045195</td><td>0.039243</td><td>0.039706</td><td>1.138237</td><td>0.255022</td><td>[-0.032, 0.122]</td></tr>
<tr><td>SLA disp:Year</td><td>0.051895</td><td>0.040612</td><td>0.041085</td><td>1.263106</td><td>0.206551</td><td>[-0.028, 0.131]</td></tr>
<tr><td>WD:Year</td><td>0.022404</td><td>0.051674</td><td>0.052300</td><td>0.428386</td><td>0.668370</td><td>[-0.079, 0.124]</td></tr>
<tr><td>Taxonomic diversity</td><td>0.006681</td><td>0.055609</td><td>0.056267</td><td>0.118735</td><td>0.905485</td><td>[-0.102, 0.116]</td></tr>
<tr><td>Phylogenetic diversity:Year</td><td>-0.027791</td><td>0.039892</td><td>0.040380</td><td>0.688236</td><td>0.491304</td><td>[-0.106, 0.050]</td></tr>
<tr><td>WD disp:Year</td><td>0.005850</td><td>0.053378</td><td>0.054019</td><td>0.108301</td><td>0.913757</td><td>[-0.099, 0.110]</td></tr>
</table>

Table S7. Multimodel inference results of Carbon losses; n = 114. Estimate, averaged model coefficient; Std. Error, standard error of the estimate; Adjusted SE, adjusted standard error; z value, test statistic; Pr(>|z|), p value from a two-sided asymptotic z test; CI 95%, 95% confidence interval of the estimates. The models were fitted with the response variable in the log-scale and centred and scaled predictors. Source data are provided as a Source Data file.

Carbon losses (Mg C ha-1 yr-1) R^2 marginal = 29%						
Predictor	Estimate	Std. Error	Adjusted SE	z value	Pr(>|z|)	CI 95%
Intercept	0.510866	0.043770	0.044294	11.533597	0.000000	[0.425, 0.597]
SLA	0.131965	0.061065	0.061655	2.140367	0.032325	[0.012, 0.252]
WD	-0.108497	0.052318	0.052926	2.049990	0.040365	[-0.211, -0.006]
SLA disp	-0.110089	0.064717	0.065441	1.682263	0.092518	[-0.237, 0.017]
Aluminum - phosphorus	-0.077008	0.058791	0.059422	1.295952	0.194992	[-0.192, 0.038]
Phosphorus -> magnesium	-0.090556	0.058841	0.059492	1.522141	0.127974	[-0.206, 0.025]
Site area	0.001013	0.054406	0.054985	0.018420	0.985304	[-0.106, 0.108]
Soil fertility	-0.037946	0.083903	0.084870	0.447111	0.654795	[-0.202, 0.127]
Cold-wet -> Warm-dry	-0.215817	0.117763	0.118901	1.815104	0.069508	[-0.447, 0.015]
Year	0.334201	0.049591	0.050150	6.664074	0.000000	[0.237, 0.431]
Cold-wet -> Warm-dry:Year	0.234645	0.072890	0.073628	3.186920	0.001438	[0.092, 0.378]
WD disp	0.067195	0.073984	0.074729	0.899185	0.368554	[-0.078, 0.212]
WD disp:Year	0.153583	0.073780	0.074401	2.064269	0.038992	[0.009, 0.298]

Carbon losses (Mg C ha-1 yr-1) R^2 marginal = 29%						
Predictor	Estimate	Std. Error	Adjusted SE	z value	Pr(>|z|)	CI 95%
WD:Year	0.072693	0.078332	0.078927	0.921019	0.357041	[-0.081, 0.226]
Taxonomic diversity	0.024848	0.060733	0.061446	0.404391	0.685925	[-0.094, 0.144]
SLA disp:Year	-0.020691	0.049232	0.049835	0.415181	0.678010	[-0.117, 0.076]
Phylogenetic diversity	0.024858	0.051936	0.052519	0.473324	0.635982	[-0.077, 0.127]
SLA:Year	0.002476	0.048033	0.048612	0.050937	0.959376	[-0.092, 0.097]

Table S8. Multimodel inference results of the carbon losses relativized by carbon stocks; n = 114. Estimate, averaged model coefficient; Std. Error, standard error of the estimate; Adjusted SE, adjusted standard error; z value, test statistic; Pr(>|z|), p value from a two-sided asymptotic z test; CI 95%, 95% confidence interval of the estimates. The models were fitted with the response variable in the log-scale and centred and scaled predictors. Source data are provided as a Source Data file.

Carbon losses (relative) R^2 marginal = 18%						
Predictor	Estimate	Std. Error	Adjusted SE	z value	Pr(>|z|)	CI 95%
Intercept	-3.980855	0.042528	0.043025	92.524688	0.000000	[-4.064, -3.897]
Aluminum - phosphorus	-0.030591	0.051454	0.052049	0.587734	0.556711	[-0.131, 0.070]
Phosphorus -> magnesium	-0.026566	0.054076	0.054705	0.485619	0.627237	[-0.133, 0.079]
Site area	0.015773	0.045683	0.046228	0.341209	0.732946	[-0.074, 0.105]
Soil fertility	-0.077174	0.078875	0.079800	0.967090	0.333499	[-0.232, 0.077]
Cold-wet -> Warm-dry	0.042482	0.107756	0.108917	0.390043	0.696505	[-0.169, 0.254]
Year	0.225384	0.050224	0.050743	4.441692	0.000009	[0.127, 0.324]
Cold-wet -> Warm-dry:Year	0.187986	0.074952	0.075633	2.485514	0.012937	[0.041, 0.335]
WD	-0.100477	0.059499	0.060100	1.671810	0.094562	[-0.217, 0.016]
WD:Year	0.143830	0.068797	0.069407	2.072259	0.038241	[0.009, 0.279]
WD disp	-0.009245	0.070357	0.071179	0.129889	0.896655	[-0.147, 0.129]
WD disp:Year	0.137956	0.059450	0.060074	2.296420	0.021652	[0.021, 0.254]
Phylogenetic diversity	0.012294	0.048608	0.049146	0.250152	0.802470	[-0.083, 0.108]

Carbon losses (relative) R^2 marginal = 18%						
Predictor	Estimate	Std. Error	Adjusted SE	z value	Pr(>|z|)	CI 95%
Taxonomic diversity	0.024991	0.056435	0.057100	0.437679	0.661619	[-0.086, 0.136]
SLA disp	-0.005910	0.052127	0.052726	0.112086	0.910755	[-0.108, 0.096]
SLA	0.016778	0.047992	0.048563	0.345493	0.729724	[-0.077, 0.111]
SLA:Year	0.055728	0.046160	0.046714	1.192951	0.232889	[-0.035, 0.146]
Taxonomic diversity:Year	0.064964	0.058271	0.058971	1.101620	0.270627	[-0.049, 0.179]

Table S9. Multimodel inference results of carbon residence time (CRT); n = 114. Estimate, averaged model coefficient; Std. Error, standard error of the estimate; Adjusted SE, adjusted standard error; z value, test statistic; Pr(>|z|), p value from a two-sided asymptotic z test; CI 95%, 95% confidence interval of the estimates. The models were fitted with the response variable in the log-scale and centred and scaled predictors. Source data are provided as a Source Data file.

CTR R^2 marginal = 34%						
Predictor	Estimate	Std. Error	Adjusted SE	z value	Pr(>|z|)	CI 95%
Intercept	3.701483	0.033644	0.034026	108.785051	0.000000	[3.636, 3.767]
SLA	0.132979	0.043252	0.043680	3.044409	0.002331	[0.048, 0.218]
WD	-0.100024	0.042333	0.042750	2.339773	0.019296	[-0.183, -0.017]
Aluminum - phosphorus	0.115180	0.042804	0.043285	2.660973	0.007792	[0.031, 0.199]
Phosphorus -> magnesium	0.004586	0.042202	0.042688	0.107434	0.914445	[-0.078, 0.087]
Site area	-0.018426	0.042649	0.043081	0.427696	0.668873	[-0.102, 0.065]
Soil fertility	-0.110778	0.061286	0.062000	1.786748	0.073978	[-0.231, 0.009]
Cold-wet -> Warm-dry	0.179405	0.106382	0.107066	1.675647	0.093807	[-0.029, 0.388]
Year	0.133720	0.034849	0.035226	3.796105	0.000147	[0.065, 0.202]
Cold-wet -> Warm-dry:Year	0.099710	0.046434	0.046969	2.122919	0.033761	[0.009, 0.191]
WD disp	0.113765	0.059223	0.059764	1.903594	0.056963	[-0.002, 0.230]
Phylogenetic diversity	-0.072232	0.039967	0.040377	1.788915	0.073629	[-0.151, 0.006]
SLA disp	-0.071311	0.051399	0.051946	1.372775	0.169823	[-0.172, 0.029]

CTR R^2 marginal = 34%						
Predictor	Estimate	Std. Error	Adjusted SE	z value	Pr(>|z|)	CI 95%
SLA:Year	-0.041269	0.034302	0.034709	1.188986	0.234445	[-0.109, 0.026]
WD:Year	-0.022018	0.047545	0.048046	0.458265	0.646762	[-0.115, 0.071]
SLA disp:Year	-0.038585	0.036212	0.036622	1.053613	0.292060	[-0.110, 0.032]
Phylogenetic diversity:Year	0.023482	0.035215	0.035641	0.658849	0.509993	[-0.046, 0.093]
Taxonomic diversity	-0.001350	0.047397	0.047955	0.028150	0.977543	[-0.094, 0.092]
WD disp:Year	0.018913	0.050510	0.050958	0.371136	0.710536	[-0.080, 0.118]
Taxonomic diversity:Year	-0.061890	0.037296	0.037753	1.639336	0.101143	[-0.135, 0.011]
