# Supplementary material for: Long-term decline in above-ground carbon sinks in Brazilian tropical and subtropical forests
Source: Nat Commun. 2026 Aug 17;17:9856. doi: 10.1038/s41467-026-74921-0 (PMC13578287; doi:10.1038/s41467-026-74921-0)
Supplement: Supplementary file 2 [file 41467_2026_74921_MOESM2_ESM.pdf]

Description of Additional Supplementary Files

File Name: Supplementary Data 1
Description: Table containing descriptive information for each forest plot included in the study: plot code (unique identifier), geographic coordinates (latitude and longitude in decimal degrees), forest type, sampled area (hectares), total number of censuses conducted, year of the first census, year of the last census, and mean interval length between consecutive censuses (years).

File Name: Supplementary Data 2
Description: List of which species were matched from the dataset to the phylogeny.

File Name: Supplementary Data 3
Description: Table containing, for each plot (identified by plot code), site metadata including geographic coordinates (latitude and longitude), soil and climate characteristics, sampling information and census dates, as well as estimates of forest carbon stocks.

File Name: Supplementary Data 4
Description: Table containing, for each plot (identified by plot code) and census interval, site metadata including geographic coordinates (latitude and longitude), soil and climate characteristics, sampling information and census dates defining each interval, as well as estimates of forest carbon dynamics.
