# Supplementary material for: Long-term decline in above-ground carbon sinks in Brazilian tropical and subtropical forests
Source: Nat Commun. 2026 Aug 17;17:9856. doi: 10.1038/s41467-026-74921-0 (PMC13578287; doi:10.1038/s41467-026-74921-0)
Supplement: Supplementary file 3 [file 41467_2026_74921_MOESM7_ESM.pdf]

Corresponding author(s): Vinícius Maia, Rubens Santos

Last updated by author(s): 03/05/2026

Reporting Summary

Nature Portfolio wishes to improve the reproducibility of the work that we publish. This form provides structure for consistency and transparency in reporting. For further information on Nature Portfolio policies, see our Editorial Policies and the Editorial Policy Checklist.

Statistics

For all statistical analyses, confirm that the following items are present in the figure legend, table legend, main text, or Methods section.

n/a	Confirmed	
☐	☒	The exact sample size (*n*) for each experimental group/condition, given as a discrete number and unit of measurement
☐	☒	A statement on whether measurements were taken from distinct samples or whether the same sample was measured repeatedly
☐	☒	The statistical test(s) used AND whether they are one- or two-sided<br>*Only common tests should be described solely by name; describe more complex techniques in the Methods section.*
☐	☒	A description of all covariates tested
☐	☒	A description of any assumptions or corrections, such as tests of normality and adjustment for multiple comparisons
☐	☒	A full description of the statistical parameters including central tendency (e.g. means) or other basic estimates (e.g. regression coefficient) AND variation (e.g. standard deviation) or associated estimates of uncertainty (e.g. confidence intervals)
☐	☒	For null hypothesis testing, the test statistic (e.g. *F*, *t*, *r*) with confidence intervals, effect sizes, degrees of freedom and *P* value noted<br>*Give P values as exact values whenever suitable.*
☒	☐	For Bayesian analysis, information on the choice of priors and Markov chain Monte Carlo settings
☐	☒	For hierarchical and complex designs, identification of the appropriate level for tests and full reporting of outcomes
☒	☐	Estimates of effect sizes (e.g. Cohen's *d*, Pearson's *r*), indicating how they were calculated

Our web collection on statistics for biologists contains articles on many of the points above.

Software and code

Policy information about availability of computer code

Data collection

No software was used to collect data.

Data analysis

R Core Team. R: A language and environment for statistical computing. Version 4.4.1. R Foundation for Statistical Computing, Vienna, Austria. https://www.r-project.org/ (2024).

Carvalho G. et al. Flora: Tools for Interacting with the Brazilian Flora 2020. R package version 0.3.4. https://CRAN.R-project.org/package=flora. (2020).

Réjou-Méchain, M., Tanguy, A., Piponiot, C., Chave, J., & Hérault, B. BIOMASS: an r package for estimating above-ground biomass and its uncertainty in tropical forests (version 2.2.4-1). Methods in Ecology and Evolution, 8(9), 1163–1167. doi: 10.1111/2041-210X.12753. (2017).

Hsieh T.C., Ma K.H. and Chao A. iNEXT: iNterpolation and EXTrapolation for species diversity. R package version 2.0.20

Wickham, H. Ggplot2: Elegant Graphics for Data Analysis (version 4.0.1). Springer-Verlag New York. ISBN 978-3-319-24277-4, https://ggplot2.tidyverse.org. (2016).

Wei, T., & Simko, V. R package "corrplot": Visualization of a Correlation Matrix (Version 0.84). Retrieved from https://github.com/taiyun/corrplot. (2017).

Garnier, S. Viridis: Default Color Maps from 'matplotlib'. R package version 0.5.1. https://CRAN.R-project.org/package=viridis. (2018).

Wilke, C. Cowplot: Streamlined Plot Theme and Plot Annotations for 'ggplot2'. R package version 1.0.0.

Schloerke, B., Cook, D., Larmarange, J., Briatte, F., Marbach, M., Thoen, E., Elberg, A. & Crowley, J. GGally: Extension to 'ggplot2'. R package version 2.1.1.

Bates, D., Mächler, M., Bolker, B., & Walker, S. Fitting linear mixed-effects models using lme4 (version 1.1-37). Journal of Statistical Software, 67(1), 1–48. doi: 10.18637/jss.v067.i01. (2015).

Wood, S. Generalized Additive Models: An Introduction with R, 2 edition (mgcv version 1.9-1). Chapman and Hall/CRC. (2017).

Bivand R.S., Pebesma E., Gomez-Rubio V. Applied spatial data analysis with R, Second edition (spdep version 1.4-2). Springer, NY. http://www.asdar-book.org/. (2013).

Lê, S., Josse, J., Husson, F. FactoMineR: A Package for Multivariate Analysis (version 2.12). Journal of Statistical Software, 25(1), 1–18. doi:10.18637/jss.v025.i01. (2008).

Kassambara, A., & Mundt, F. Factoextra: Extract and Visualize the Results of Multivariate Data Analyses. R package version 1.0.7. https://CRAN.R-project.org/package=factoextra. (2020).

R version: R 4.4.1,

Packages: flora 0.3.4, BIOMASS 2.2.4-1, iNEXT 2.0.20, ggplot2 4.0.1, corrplot 0.84, viridis 0.5.1, cowplot 1.0.0, GGally 2.1.1, lme4 1.1-37, mgcv 1.9-1, spdep 1.4-2, FactoMineR 2.12, factoextra 1.0.7, MuMIn 1.42.1.

The code of the manuscript is publicly available at https://doi.org/10.5281/zenodo.18881288

For manuscripts utilizing custom algorithms or software that are central to the research but not yet described in published literature, software must be made available to editors and reviewers. We strongly encourage code deposition in a community repository (e.g. GitHub). See the Nature Portfolio guidelines for submitting code & software for further information.

Data

Policy information about availability of data

All manuscripts must include a data availability statement. This statement should provide the following information, where applicable:

- Accession codes, unique identifiers, or web links for publicly available datasets
- A description of any restrictions on data availability
- For clinical datasets or third party data, please ensure that the statement adheres to our policy

Data Availability

The data generated in this study are provided in the Supplementary Data files. The raw tree-level forest inventory data are available under restricted access because they are subject to third-party data-use agreements defined by ForestPlots.net (www.forestplots.net) (see area codes in Supplementary Data 1); access can be obtained by submitting a request directly to ForestPlots.net in accordance with their data access policies. These raw data are not publicly available. The Supplementary Data files contain all processed data necessary to evaluate the conclusions of the paper (census level: Supplementary Data 3; census-interval level: Supplementary Data 4). Climate data were obtained from the Climatic Research Unit (CRU TS version 4.04; released 24 April 2020; https://crudata.uea.ac.uk/cru/data/hrg) and WorldClim (https://www.worldclim.org/). Phylogenetic data were obtained from Constructing a broadly inclusive seed plant phylogeny (https://doi.org/10.1002/ajb2.1019). Trait data were compiled from the following sources: Conservative species drive biomass productivity in tropical dry forests (https://doi.org/10.1111/1365-2745.12543); Towards a Worldwide Wood Economics Spectrum (https://doi.org/10.5061/dryad.234); Bamboo dominance in an Atlantic Forest community: a structural, functional and taxonomic approach (https://repositorio.ufjf.br/jspui/handle/ufjf/10249); Trait diversity on the phylogeny of cerrado woody species (https://doi.org/10.1111/j.1600-0706.2011.19513.x); Phylogeny, traits, environment, and space in cerrado plant communities at Emas National Park (Brazil) (https://doi.org/10.1016/j.flora.2011.07.004); The influence of fire on phylogenetic and functional structure of woody savannas: Moving from species to individuals (https://doi.org/10.1016/j.ppees.2011.11.004); Megafauna biogeography explains plant functional trait variability in the tropics (https://doi.org/10.1111/geb.13111); Leaf habit does not predict leaf functional traits in cerrado woody species (https://doi.org/10.1016/j.baae.2013.05.002); and Shifting species and functional diversity due to abrupt changes in water availability in tropical dry forests (https://doi.org/10.1111/1365-2745.13031). Map data was obtained from INSTITUTO BRASILEIRO DE GEOGRAFIA E ESTATÍSTICA (IBGE) https://www.ibge.gov.br/geociencias/organizacao-do-territorio/malhas-territoriais/15774-malhas.html?=&t=acesso-ao-produto. Source data are provided with this paper.

Code Availability

The code is publicly available at https://doi.org/10.5281/zenodo.18881288

Research involving human participants, their data, or biological material

Policy information about studies with human participants or human data. See also policy information about sex, gender (identity/presentation), and sexual orientation and race, ethnicity and racism.

Reporting on sex and gender — *Use the terms sex (biological attribute) and gender (shaped by social and cultural circumstances) carefully in order to avoid confusing both terms. Indicate if findings apply to only one sex or gender; describe whether sex and gender were considered in study design; whether sex and/or gender was determined based on self-reporting or assigned and methods used.*

	Provide in the source data disaggregated sex and gender data, where this information has been collected, and if consent has been obtained for sharing of individual-level data; provide overall numbers in this Reporting Summary. Please state if this information has not been collected.<br>*Report sex- and gender-based analyses where performed, justify reasons for lack of sex- and gender-based analysis.*
Reporting on race, ethnicity, or other socially relevant groupings	*Please specify the socially constructed or socially relevant categorization variable(s) used in your manuscript and explain why they were used. Please note that such variables should not be used as proxies for other socially constructed/relevant variables (for example, race or ethnicity should not be used as a proxy for socioeconomic status).*<br>*Provide clear definitions of the relevant terms used, how they were provided (by the participants/respondents, the researchers, or third parties), and the method(s) used to classify people into the different categories (e.g. self-report, census or administrative data, social media data, etc.)*<br>*Please provide details about how you controlled for confounding variables in your analyses.*
Population characteristics	*Describe the covariate-relevant population characteristics of the human research participants (e.g. age, genotypic information, past and current diagnosis and treatment categories). If you filled out the behavioural & social sciences study design questions and have nothing to add here, write "See above."*
Recruitment	*Describe how participants were recruited. Outline any potential self-selection bias or other biases that may be present and how these are likely to impact results.*
Ethics oversight	*Identify the organization(s) that approved the study protocol.*

Note that full information on the approval of the study protocol must also be provided in the manuscript.

Field-specific reporting

Please select the one below that is the best fit for your research. If you are not sure, read the appropriate sections before making your selection.

☐ Life sciences ☐ Behavioural & social sciences ☒ Ecological, evolutionary & environmental sciences

For a reference copy of the document with all sections, see nature.com/documents/nr-reporting-summary-flat.pdf

Life sciences study design

All studies must disclose on these points even when the disclosure is negative.

Sample size	*Describe how sample size was determined, detailing any statistical methods used to predetermine sample size OR if no sample-size calculation was performed, describe how sample sizes were chosen and provide a rationale for why these sample sizes are sufficient.*
Data exclusions	*Describe any data exclusions. If no data were excluded from the analyses, state so OR if data were excluded, describe the exclusions and the rationale behind them, indicating whether exclusion criteria were pre-established.*
Replication	*Describe the measures taken to verify the reproducibility of the experimental findings. If all attempts at replication were successful, confirm this OR if there are any findings that were not replicated or cannot be reproduced, note this and describe why.*
Randomization	*Describe how samples/organisms/participants were allocated into experimental groups. If allocation was not random, describe how covariates were controlled OR if this is not relevant to your study, explain why.*
Blinding	*Describe whether the investigators were blinded to group allocation during data collection and/or analysis. If blinding was not possible, describe why OR explain why blinding was not relevant to your study.*

Behavioural & social sciences study design

All studies must disclose on these points even when the disclosure is negative.

Study description	*Briefly describe the study type including whether data are quantitative, qualitative, or mixed-methods (e.g. qualitative cross-sectional, quantitative experimental, mixed-methods case study).*
Research sample	*State the research sample (e.g. Harvard university undergraduates, villagers in rural India) and provide relevant demographic information (e.g. age, sex) and indicate whether the sample is representative. Provide a rationale for the study sample chosen. For studies involving existing datasets, please describe the dataset and source.*
Sampling strategy	*Describe the sampling procedure (e.g. random, snowball, stratified, convenience). Describe the statistical methods that were used to predetermine sample size OR if no sample-size calculation was performed, describe how sample sizes were chosen and provide a rationale for why these sample sizes are sufficient. For qualitative data, please indicate whether data saturation was considered, and what criteria were used to decide that no further sampling was needed.*
Data collection	*Provide details about the data collection procedure, including the instruments or devices used to record the data (e.g. pen and paper, computer, eye tracker, video or audio equipment) whether anyone was present besides the participant(s) and the researcher, and whether the researcher was blind to experimental condition and/or the study hypothesis during data collection.*
Timing	*Indicate the start and stop dates of data collection. If there is a gap between collection periods, state the dates for each sample*

	cohort.
Data exclusions	*If no data were excluded from the analyses, state so OR if data were excluded, provide the exact number of exclusions and the rationale behind them, indicating whether exclusion criteria were pre-established.*
Non-participation	*State how many participants dropped out/declined participation and the reason(s) given OR provide response rate OR state that no participants dropped out/declined participation.*
Randomization	*If participants were not allocated into experimental groups, state so OR describe how participants were allocated to groups, and if allocation was not random, describe how covariates were controlled.*

Ecological, evolutionary & environmental sciences study design

All studies must disclose on these points even when the disclosure is negative.

Study description	Our study uses data from 49 forest sites distributed along the Brazilian territory. All sites were measured at least twice, totalizing 163 census (carbon stocks level), for carbon dynamics level, our we have 114 census intervals. Therefore, we have 163 censuses nested within 49 sites and 144 census intervals nested within 49 sites. We investigated the long-term trends of carbon variables, diversity (taxonomic, phylogenetic and functional) and functional composition. We also tested the effects of these variables on the carbon variables, allowing them to interact with time, so we tested if the carbon sink of the most diverse forests had a more stable temporal trend, for example.
Research sample	long-term dataset of 49 forest sites 49 forest sites, Encompassing 163 census, 114 census intervals
Sampling strategy	No sample size calculation was performed. We selected all available plots with more than one census.
Data collection	In the field, trees with a quadratic mean diameter at 1.3 m (DBH ≥ 5 cm) were measured in each census. The quadratic mean diameter (√ d12+d22+dn2; where d is the diameter at 1.3 m of each stem of a given tree) allows the inclusion of trees with multiple stems (stems with d < 5cm) if the quadratic mean diameter of the tree reaches 5 cm (MacDicken 1991). However, in 8 semideciduous forests sites trees with at least one stem with d DBH ≥ 5 cm were measured. The height of the point of measurement (POM) was marked in each stem of each tree and used as a reference for the ensuing censuses in most sites. See figures S2 and S3 for information on site structure (DBH distribution). Plant taxonomy was based on Angiosperm Phylogeny Group IV (APG IV 2016) and species names were standardized using Flora do Brasil 2020, for this we used the R package flora (Carvalho et al. 2020). We dropped Arecaceae, Dicksoniaceae, Cyateaceae, and Cactaceae (~1% of the stems) to avoid biases in biomass calculation.
Timing and spatial scale	We used forest data from 49 repeatedly measured permanent forest plots in Brazil (comprising deciduous, semideciduous, evergreen and subtropical forests, supplementary file 1; data of 32 sites were used in Maia et al. 2020). The sites are located in south and southeastern Brazil, spanning the latitudes 28º 19’ S to 14º 16’ S, encompassing both tropical and subtropical regions (Figure 1). Sites’ mean annual temperature (MAT) ranged from 13.3 to 25.2 º C (mean 20.6 ºC), mean annual precipitation (MAP) between 669 and 1998 mm (mean 1450 mm) (climate data obtention is detailed below) and altitude ranged from 450 to 1536 m (mean 900 m); altitude was negatively correlated with MAT (r = -0.85). Note that MAT and MAP follow the latitudinal gradient, whereby MAT increases and MAP decreases northwards (Figure S1). The driest and warmest sites are deciduous forests located northward and established on fertile soils, while the coldest and wettest sites are tropical evergreen forests (in southeast Brazil) and subtropical forests (in southern Brazil). The semideciduous forests are under an intermediate climate in the tropical region.<br>Spatial sampling spanned from 0.2 to 5 ha (mean of 1.03 ha) per site, totalling 50.4 ha. Sampling was carried out using subplots of 400 m² (in most cases), which are located mainly in the interior of the sites, but edges were also captured because sampling was designed to cover within-site heterogeneity in most sites. The number of censuses per site ranges from 2 to 8 (mean 3), the total monitoring length varied from 1 to 31 years (mean 11 years) (Figure S1). The first and last census varies with site, but the first census was in 1987 and the last in 2020. The length of the census intervals varied among the sites and within the same site over time, between 1 and 9 years (mean 5 years) (Figure S1). Forest census data is hosted in the ForestPlots.net (https://www.forestplots.net).
Data exclusions	We dropped Arecaceae, Dicksoniaceae, Cyatheaceae, and Cactaceae (~1% of the stems) to avoid biases in biomass calculation.
Reproducibility	Our analysis does not include experimental findings.
Randomization	*Describe how samples/organisms/participants were allocated into groups. If allocation was not random, describe how covariates were controlled. If this is not relevant to your study, explain why.*
Blinding	*Describe the extent of blinding used during data acquisition and analysis. If blinding was not possible, describe why OR explain why blinding was not relevant to your study.*

Did the study involve field work? ☒ Yes ☐ No

Field work, collection and transport

Field conditions	The data was collected during decades by different people, and the field conditions during data collection don't affect the measurements.
Location	The study includes forest sites spread across southeastern and southern Brazil, encompassing deciduous, semideciduous, evergreen and mixed forests. The exact locations of the sites are provided in the figures and supplementary data of the paper.

Access & import/export	Field measurements were conducted with permission from landowners when sites were located on private properties. For sites located within protected areas or lands administered by public institutions, access and research activities were carried out with authorization from the responsible authorities. In Brazil, this commonly includes agencies such as ICMBio (Chico Mendes Institute for Biodiversity Conservation) and other relevant environmental management bodies. These forest monitoring plots are part of long-term research efforts that have been maintained for decades, with repeated measurements conducted under the appropriate institutional permissions and in compliance with local, national, and international regulations governing research access and environmental monitoring.
Disturbance	No significant disturbances were caused by our field measurements. Trees were tagged using a single aluminum nail (no iron), avoiding damage to trees due to corrosion.

Reporting for specific materials, systems and methods

We require information from authors about some types of materials, experimental systems and methods used in many studies. Here, indicate whether each material, system or method listed is relevant to your study. If you are not sure if a list item applies to your research, read the appropriate section before selecting a response.

Materials & experimental systems

n/a	Involved in the study	
☒	☐	Antibodies
☒	☐	Eukaryotic cell lines
☒	☐	Palaeontology and archaeology
☒	☐	Animals and other organisms
☒	☐	Clinical data
☒	☐	Dual use research of concern
☐	☒	Plants

Methods

n/a	Involved in the study	
☒	☐	ChIP-seq
☒	☐	Flow cytometry
☒	☐	MRI-based neuroimaging

Antibodies

Antibodies used	*Describe all antibodies used in the study; as applicable, provide supplier name, catalog number, clone name, and lot number.*
Validation	*Describe the validation of each primary antibody for the species and application, noting any validation statements on the manufacturer's website, relevant citations, antibody profiles in online databases, or data provided in the manuscript.*

Eukaryotic cell lines

Policy information about cell lines and Sex and Gender in Research

Cell line source(s)	*State the source of each cell line used and the sex of all primary cell lines and cells derived from human participants or vertebrate models.*
Authentication	*Describe the authentication procedures for each cell line used OR declare that none of the cell lines used were authenticated.*
Mycoplasma contamination	*Confirm that all cell lines tested negative for mycoplasma contamination OR describe the results of the testing for mycoplasma contamination OR declare that the cell lines were not tested for mycoplasma contamination.*
Commonly misidentified lines (See ICLAC register)	*Name any commonly misidentified cell lines used in the study and provide a rationale for their use.*

Palaeontology and Archaeology

Specimen provenance	*Provide provenance information for specimens and describe permits that were obtained for the work (including the name of the issuing authority, the date of issue, and any identifying information). Permits should encompass collection and, where applicable, export.*
Specimen deposition	*Indicate where the specimens have been deposited to permit free access by other researchers.*
Dating methods	*If new dates are provided, describe how they were obtained (e.g. collection, storage, sample pretreatment and measurement), where they were obtained (i.e. lab name), the calibration program and the protocol for quality assurance OR state that no new dates are provided.*

☐ Tick this box to confirm that the raw and calibrated dates are available in the paper or in Supplementary Information.

Ethics oversight	*Identify the organization(s) that approved or provided guidance on the study protocol, OR state that no ethical approval or guidance was required and explain why not.*

Note that full information on the approval of the study protocol must also be provided in the manuscript.

Animals and other research organisms

Policy information about studies involving animals; ARRIVE guidelines recommended for reporting animal research, and Sex and Gender in Research

Laboratory animals	*For laboratory animals, report species, strain and age OR state that the study did not involve laboratory animals.*
Wild animals	*Provide details on animals observed in or captured in the field; report species and age where possible. Describe how animals were caught and transported and what happened to captive animals after the study (if killed, explain why and describe method; if released, say where and when) OR state that the study did not involve wild animals.*
Reporting on sex	*Indicate if findings apply to only one sex; describe whether sex was considered in study design, methods used for assigning sex. Provide data disaggregated for sex where this information has been collected in the source data as appropriate; provide overall numbers in this Reporting Summary. Please state if this information has not been collected. Report sex-based analyses where performed, justify reasons for lack of sex-based analysis.*
Field-collected samples	*For laboratory work with field-collected samples, describe all relevant parameters such as housing, maintenance, temperature, photoperiod and end-of-experiment protocol OR state that the study did not involve samples collected from the field.*
Ethics oversight	*Identify the organization(s) that approved or provided guidance on the study protocol, OR state that no ethical approval or guidance was required and explain why not.*

Note that full information on the approval of the study protocol must also be provided in the manuscript.

Clinical data

Policy information about clinical studies

All manuscripts should comply with the ICMJE guidelines for publication of clinical research and a completed CONSORT checklist must be included with all submissions.

Clinical trial registration	*Provide the trial registration number from ClinicalTrials.gov or an equivalent agency.*
Study protocol	*Note where the full trial protocol can be accessed OR if not available, explain why.*
Data collection	*Describe the settings and locales of data collection, noting the time periods of recruitment and data collection.*
Outcomes	*Describe how you pre-defined primary and secondary outcome measures and how you assessed these measures.*

Dual use research of concern

Policy information about dual use research of concern

Hazards

Could the accidental, deliberate or reckless misuse of agents or technologies generated in the work, or the application of information presented in the manuscript, pose a threat to:

No	Yes	
☒	☐	Public health
☒	☐	National security
☒	☐	Crops and/or livestock
☐	☒	Ecosystems
☒	☐	Any other significant area

Hazards	*Please describe the agents/technologies/information that may pose a threat, including any agents subject to oversight for dual use research of concern.*

For examples of agents subject to oversight, see the United States Government Policy for Institutional Oversight of Life Sciences Dual Use Research of Concern.

Experiments of concern

Does the work involve any of these experiments of concern:

No	Yes	
☒	☐	Demonstrate how to render a vaccine ineffective
☒	☐	Confer resistance to therapeutically useful antibiotics or antiviral agents
☒	☐	Enhance the virulence of a pathogen or render a nonpathogen virulent
☒	☐	Increase transmissibility of a pathogen
☒	☐	Alter the host range of a pathogen
☒	☐	Enable evasion of diagnostic/detection modalities
☒	☐	Enable the weaponization of a biological agent or toxin
☒	☐	Any other potentially harmful combination of experiments and agents

Precautions and benefits

Biosecurity precautions	*Describe the precautions that were taken during the design and conduct of this research, or will be required in the communication and application of the research, to minimise biosecurity risks. These may include bio-containment facilities, changes to the study design/ methodology or redaction of details from the manuscript.*
Biosecurity oversight	*Describe any evaluations and oversight of biosecurity risks of this work that you have received from people or organizations outside of your immediate team.*
Benefits	*Describe the benefits that application or use of this work could bring, including benefits that may mitigate risks to public health, national security, or the health of crops, livestock or the environment.*
Communication benefits	*Describe whether the benefits of communicating this information outweigh the risks, and if so, how.*

Plants

Seed stocks	*Report on the source of all seed stocks or other plant material used. If applicable, state the seed stock centre and catalogue number. If plant specimens were collected from the field, describe the collection location, date and sampling procedures.*
Novel plant genotypes	*Describe the methods by which all novel plant genotypes were produced. This includes those generated by transgenic approaches, gene editing, chemical/radiation-based mutagenesis and hybridization. For transgenic lines, describe the transformation method, the number of independent lines analyzed and the generation upon which experiments were performed. For gene-edited lines, describe the editor used, the endogenous sequence targeted for editing, the targeting guide RNA sequence (if applicable) and how the editor was applied.*
Authentication	*Describe any authentication procedures for each seed stock used or novel genotype generated. Describe any experiments used to assess the effect of a mutation and, where applicable, how potential secondary effects (e.g. second site T-DNA insertions, mosiacism, off-target gene editing) were examined.*

ChIP-seq

Data deposition

☐ Confirm that both raw and final processed data have been deposited in a public database such as GEO.

☐ Confirm that you have deposited or provided access to graph files (e.g. BED files) for the called peaks.

Data access links<br>*May remain private before publication.*	*For "Initial submission" or "Revised version" documents, provide reviewer access links. For your "Final submission" document, provide a link to the deposited data.*
Files in database submission	*Provide a list of all files available in the database submission.*
Genome browser session<br>(e.g. UCSC)	*Provide a link to an anonymized genome browser session for "Initial submission" and "Revised version" documents only, to enable peer review. Write "no longer applicable" for "Final submission" documents.*

Methodology

Replicates	*Describe the experimental replicates, specifying number, type and replicate agreement.*
Sequencing depth	*Describe the sequencing depth for each experiment, providing the total number of reads, uniquely mapped reads, length of reads and whether they were paired- or single-end.*
Antibodies	*Describe the antibodies used for the ChIP-seq experiments; as applicable, provide supplier name, catalog number, clone name, and lot number.*

Peak calling parameters	*Specify the command line program and parameters used for read mapping and peak calling, including the ChIP, control and index files used.*
Data quality	*Describe the methods used to ensure data quality in full detail, including how many peaks are at FDR 5% and above 5-fold enrichment.*
Software	*Describe the software used to collect and analyze the ChIP-seq data. For custom code that has been deposited into a community repository, provide accession details.*

Flow Cytometry

Plots

Confirm that:

☐ The axis labels state the marker and fluorochrome used (e.g. CD4-FITC).

☐ The axis scales are clearly visible. Include numbers along axes only for bottom left plot of group (a 'group' is an analysis of identical markers).

☐ All plots are contour plots with outliers or pseudocolor plots.

☐ A numerical value for number of cells or percentage (with statistics) is provided.

Methodology

Sample preparation	*Describe the sample preparation, detailing the biological source of the cells and any tissue processing steps used.*
Instrument	*Identify the instrument used for data collection, specifying make and model number.*
Software	*Describe the software used to collect and analyze the flow cytometry data. For custom code that has been deposited into a community repository, provide accession details.*
Cell population abundance	*Describe the abundance of the relevant cell populations within post-sort fractions, providing details on the purity of the samples and how it was determined.*
Gating strategy	*Describe the gating strategy used for all relevant experiments, specifying the preliminary FSC/SSC gates of the starting cell population, indicating where boundaries between "positive" and "negative" staining cell populations are defined.*

☐ Tick this box to confirm that a figure exemplifying the gating strategy is provided in the Supplementary Information.

Magnetic resonance imaging

Experimental design

Design type	*Indicate task or resting state; event-related or block design.*
Design specifications	*Specify the number of blocks, trials or experimental units per session and/or subject, and specify the length of each trial or block (if trials are blocked) and interval between trials.*
Behavioral performance measures	*State number and/or type of variables recorded (e.g. correct button press, response time) and what statistics were used to establish that the subjects were performing the task as expected (e.g. mean, range, and/or standard deviation across subjects).*

Acquisition

Imaging type(s)	*Specify: functional, structural, diffusion, perfusion.*
Field strength	*Specify in Tesla*
Sequence & imaging parameters	*Specify the pulse sequence type (gradient echo, spin echo, etc.), imaging type (EPI, spiral, etc.), field of view, matrix size, slice thickness, orientation and TE/TR/flip angle.*
Area of acquisition	*State whether a whole brain scan was used OR define the area of acquisition, describing how the region was determined.*

Diffusion MRI ☐ Used ☐ Not used

Preprocessing

Preprocessing software	*Provide detail on software version and revision number and on specific parameters (model/functions, brain extraction, segmentation, smoothing kernel size, etc.).*
Normalization	*If data were normalized/standardized, describe the approach(es): specify linear or non-linear and define image types used for transformation OR indicate that data were not normalized and explain rationale for lack of normalization.*

Normalization template	*Describe the template used for normalization/transformation, specifying subject space or group standardized space (e.g. original Talairach, MNI305, ICBM152) OR indicate that the data were not normalized.*
Noise and artifact removal	*Describe your procedure(s) for artifact and structured noise removal, specifying motion parameters, tissue signals and physiological signals (heart rate, respiration).*
Volume censoring	*Define your software and/or method and criteria for volume censoring, and state the extent of such censoring.*

Statistical modeling & inference

Model type and settings	*Specify type (mass univariate, multivariate, RSA, predictive, etc.) and describe essential details of the model at the first and second levels (e.g. fixed, random or mixed effects; drift or auto-correlation).*
Effect(s) tested	*Define precise effect in terms of the task or stimulus conditions instead of psychological concepts and indicate whether ANOVA or factorial designs were used.*

Specify type of analysis: ☐ Whole brain ☐ ROI-based ☐ Both

Statistic type for inference<br>(See Eklund et al. 2016)	*Specify voxel-wise or cluster-wise and report all relevant parameters for cluster-wise methods.*
Correction	*Describe the type of correction and how it is obtained for multiple comparisons (e.g. FWE, FDR, permutation or Monte Carlo).*

Models & analysis

n/a	Involved in the study
☐	☐ Functional and/or effective connectivity
☐	☐ Graph analysis
☐	☐ Multivariate modeling or predictive analysis

Functional and/or effective connectivity	*Report the measures of dependence used and the model details (e.g. Pearson correlation, partial correlation, mutual information).*
Graph analysis	*Report the dependent variable and connectivity measure, specifying weighted graph or binarized graph, subject- or group-level, and the global and/or node summaries used (e.g. clustering coefficient, efficiency, etc.).*
Multivariate modeling and predictive analysis	*Specify independent variables, features extraction and dimension reduction, model, training and evaluation metrics.*
