# Supplementary material for: Long-term decline in above-ground carbon sinks in Brazilian tropical and subtropical forests
Source: Nat Commun. 2026 Aug 17;17:9856. doi: 10.1038/s41467-026-74921-0 (PMC13578287; doi:10.1038/s41467-026-74921-0)
Supplement: Supplementary file 4 [file 41467_2026_74921_MOESM8_ESM.pdf]

nature portfolio

Peer Review File

Long-term decline in above-ground carbon sinks in Brazilian tropical and subtropical forests

Corresponding Author: Mr Vinícius Maia

This file contains all reviewer reports in order by version, followed by all author rebuttals in order by version.

Version 0:

Reviewer comments:

Reviewer #1

(Remarks to the Author)
The authors use a long-term and widespread dataset from south and southeastern Brazil tropical and subtropical forests to investigate the long-term trends in carbon stocks, carbon dynamics and diversity. Besides taxonomic diversity the authors also calculated functional and phylogenetic diversity and the community level trait values for wood density and specific leaf area functional traits. The authors focus on finding out if carbon dynamics of the most diverse forests were more stable than those of the less diverse forests. The paper shows interesting analyses and important results, it is well written, with most methods and results supporting the conclusions of the authors.
Below I describe some concerns that I have for the text across the manuscript, in some cases specifically for some of the results and for some of the statements from the discussion that I do not think are totally supported by the results shown.

1) Overall, it seems that there is an excessive number of figures and if this is an issue perhaps the authors could keep the most relevant in the main text such as 2-6, 9,10 and leave the others as supplementary material. Please also include the R codes used to run the analysis as to have a clearer idea of the process and how the results were obtained.
1.1) A sthe authors include data for Deciduous forests, and deciduousness can be see as an adaptation to dry environments, which may have implications for the adaptation of species to a drying climate it would be great if the authors could include 'deciduousness' as a functional trait in their analysis. Perhaps deciduousness can explain more of the changes, on top of the already explained variance, in the carbon metrics analysed.

2) Results

L 153 - 154 - why? communities may be getting more deciduous as a strategy in comparison to acquiring more conservative traits. Perhaps the authors could also discuss this possibility.
155-157 → But also as an adaptation to lower water availability
160-163 → Unless they were already at their physiological limits of water availability and further droughts may push them towards alternative states.
180 → Exemplifies my points above regarding deciduous trees as method of adaptation to drier conditions.
187 → Please clarify if the "subplots" are the measuring unit of the analysis-thus the 49 plots
189-190 → How do you think edge effects could be driving your results? Please also specify the number of plots that are within 100m of the forest edge. Perhaps being at the forest edge has an impact on microclimate conditions and affects community composition and phenological responses? e. g. Nunes, M.H., Camargo, J.L.C., Vincent, G. et al. Forest fragmentation impacts the seasonality of Amazonian evergreen canopies. Nat Commun 13, 917 (2022). https://doi.org/10.1038/s41467-022-28490-7
225 → "estimations from"
280-284 → Please provide information about the traits assignation to the genus level and family level as well.
301-302 → While using a matrix of species abundance as input is feasible such data may not relate directly to the DBH or Basal area of the individuals in the plot, which greatly also relates to the competition between individuals and their functional composition. Therefore, I would advice to rerun the functional diversity analyses using the commutative basal area of the species, something that would also relate more with how the calculations for the other metrics were made.
332-333 → Same than above in line 301-302

3) Climate Data

361-362 → please provide the reasoning for defining the climate of a plot for a given census by the 5 preceding years, up to now it seems subjective.
194-163 → Introduction, at the end: The authors only seem to make the argument for the impact of climate but do not mention the impact of soil conditions in the diversity, carbon stocks and dynamics. However, soil data is presented in the methods and used in analysis and results, something with which I agree. Therefore, it would be great if the authors could include some information about the role of soil in the introduction section besides the short paragraph in lines 95 to 98.
119- 129 → I do not totally agree with this sentence. Grime 1998 does not suggest this only for low diversity ecosystems. In high diversity ecosystems the most abundant species tend to have more weight in the functioning of the system. Thus I would rephrase this sentence accounting for this observation. Eg. Van der Sande, Masha T., et al. "Old-growth Neotropical forests are shifting in species and trait composition." Ecological Monographs 86.2 (2016): 228-243.
135 → It would be great to see other references than that of Prado-Junior et al. 2016 when argumenting about the role of plant traits and the relation to carbon stock and the other questions addressed. There is much more literature, including recent one, about this out there.
136-140 → Please also discuss the role of deciduousness here, as this may change the way how plant species behave in such environments in comparison to other acquisitive ones with similar characteristics but not being deciduous

416 → Please specify what are the results for the Moran I analysis and if needed how did the authors accounted for the spatial autocorrelation in the models. If the Moran I test indicates the authors need to account for the spatial autocorrelation and they have not done it then it would be great if the authors can account for this in new analyses.

524-520 → Although I simpatize with the idea of describing the patterns obtained despite the P-values not being significant, in this case the authors are using P-values making figures of model results with no significant effects which can be misleading. E.g. Figure 10A for WD dispersion by year. Please include slope and P-values also for Figure 10, so the reader can judge the strength and significance of effects.
710-712 → But the results from the models do not show significant effects of F. Div. Why are the authors stating this? Either rephrase this specifically stating this effect is not significant or remove this from the text. Moreover, as far as I can see from the results the F. Div effect was only found for the "relative carbon sink' and not for the other metrics, however, the general text across these lines seems to infer that overall forests with high functional diversity were less resilient, which is also in contrast to other recent analysis. (e.g. Aguirre-Gutierrez et al 2022 Nature Ecol. Evol.)
710-712 → Moreover, the authors only used two functional traits to calculate the F. Div. metrics, which hardly may depict the functional composition and diversity of the forests analysis. I would advise on rephrasing the text along these lines (710-712).
710 - 712 → Given that the authors only used two functional traits to calculate the F. Div., that they also use the single traits in their models and because most results related to F. Div. are not significant I would strongly advise on leaving this metric out of their analysis for the final version of the manuscript. Again, in my opinion using only two traits hardly represent the functional diversity and strategies tehreon of the forests analysed.
708-709 → But WDd, had significant negative effects on Net and Relative C sink, thus this does not seem to support the statement presented across these lines.
Figure 10 →Please include significance levels
713-714 → Perhaps for SLA for Net C sink only because the results in Figures 7 and Figure 8 do not show any significant effect for WD. Please rephrase the text here and be specific about the findings.
714-715 → Strange sentence, please rephrase.
718-719→ Let the reader decide this, better eliminate this sentence.

Muito bom manuscrito, foi um prazer ler o estudo.

Jesus Aguirre Gutierrez

Reviewer #2

(Remarks to the Author)
The manuscript by Maia et al. investigates the temporal trend of forest carbon, diversity and composition along a climate gradient in Brazil. The paper is based on a good forest plot dataset with a remarquable effort done on floristic identification, soil measurements and plot remeasurement. It is a sequel of a recent paper led by part of the same group that focused on 32 out of the 49 plots used in the present study but, here, the main originality is to bring diversity in the analyses. I congratulate the authors for this effort. The results are interesting and of wide interest but need to be better statistically assessed in my opinion. The paper is relatively well written even if not always easy to follow, especially in the material and method and results sections due to the large number of analyses done and a lack of justification for some of the analyses as pointed out in my specific comments below. My overall assessment is positive but here is below some major concerns and several specific ones (that should not be always considered as minor).

Major concerns

There is little discussion and evidence of the absence of historical disturbances in the studied plots. I know that this kind of information is hard to obtain but given their importance in the studied topics, it should, in my opinion, be better discussed. Speaking of that, the diameter distribution shown in Figure S2 suggests that several sites suffer from a striking lack of tree regeneration (e.g. LAG_07, LUM_01). How can it be explained ?

The analysis on the impact of climate on AGC stocks is circular and likely to be impacted by AGB calculation methodology. The authors use the Chave E parameter to model differences in the height-diameter relationship among plots. This E parameter is derived from climatic data so that AGC estimates at least partly derived from climatic data that are then used to predict AGC… Furthermore, the use of several AGB equations, or their mixing, may introduce significant bias between groups of sites located along the climate gradient. The best solution would be to apply the same biomass equation (Chave Eq4 for instance) using site-specific height-diameter allometries. If local tree height data are not available, regional ones, such as the Feldpausch equation, would be, in my opinion, better conceptually.

My most important concern is about the way the models have been implemented. I do have at least 4 major concerns here :

1) Because the plot is the statistical unit, implementing a random effect on it is stricly equivalent to implementing a fixed plot effect. It results on a model with no degree of freedom, which may lead to wrong inferences.

2) According to the map of the study sites, the forest plots are not well distributed along the climatic gradient but spatially clustered in four locations. Thus, spatial autocorrelation in climate, soil, forest composition, etc. is likely very high and probably strongly impacted the model. Note that assessing the autocorrelation in the residuals does not control for the influence of this spatial autocorrelation because spatial components have been probably captured in the model.

One potential solution to solve problems 1 and 2 is to use the location (n=4) as a random effect (rather than the plots themselve), to account for the non-independence of plots located in the same location and thus adress the autocorrelation issue.

3) The models were implemented with a huge number of parameters due to the inclusion of several predictors and interactions. The problem is that the number of statistical units does not allow to account for such a large number of parameters to estimate. I even can hardly imagine how models can be succesfully fitted given that plot identity was also considered as a random effect (see point 1). I believe that this may have resulted in overfitting issues that, in turn, may have generated difficult to interpret results (e.g. the fact that WD and SLA dispersion provided opposite result to that obtain with functional diversity on the long term stability for instance). The consequence is that the authors have to find a « plausible » explanation for each of the (sometimes contradictory) results and that, at the end, it is quite hard to get a take home message.

4) Few plot measurements were available before 2000 so that some of the relationships should be carefully interpreted (e.g. Fig. 4A, 4E, 4F, 5B) as it appears that only a few observations (n<4) significantly drove the functional form of the predictions. Similarly, several significant results seem to be the result of a few observations only, rather than a general trend (e.g. Fig. 6 and Fig. 9A). The authors should at least acknowledge this and carefully check those peculiar observations.

Finally, given the topic of the paper, I was surprise not to see any discussion on the carbon residence time.

Specific comments

Lines 48-49 : « context-dependent » seems inappropriate here as the sentence only suggests that the results depend on the biodiversity metrics used.

Line 71 : I would replace « ecological metrics » by « ecological dimensions » here.

Line 76 : idem or even better, I would simply say : « However, it is largely unknown how diversity affects…. » to better link with the previous sentence. BTW, I am not a native english speaker but « Brazilian neglected non-Amazon forests » sounds weird to me (if ok don't worry about this comment).

Line 79 : I would rather say « with functional trade offs more associated…. »

Line 80-83 : Please reformulate : insights cannot be crucial to understand.

Line 109 : What do you mean by « temporal complementarity» ? Also, please consider mentioning the « Insurance hypothesis » by Yachi & Loreau 1999 PNAS.

Line 120 : Can we really simply summarize the mass ratio hypothesis to « functional composition » ?

line 149 : Please change to « long-term dataset of 49 forest plots ». You do not have really 49 sites according to the map or if you want to keep site everywhere in the ms, you should provide information on the minimum distance between the studied plots.

Line 153 : « ecological factors » is inappropriate here. Biotic conditions or forest composition would be more appropriated for instance.

Lines 154-155 : It would be better to have a proper expectation here. For instance, if you hypothesize that the forest will be more dominated by species with conservative strategies, it should in turn decrease the C productivity.

Lines 188-190 : Not sure to understand this sentence.

Line 191-192 : How can you estimate C dynamics with only one year census ?!

Line 194 : Change to : « hosted in the ForestPlots.net platform »

Line 200 : This is not a quadratic mean. To get a mean you should divide the quadratic sum by the number of stems, but it is not what you want here so please just reformulate the wording.

Line 215 : Provide references for the global wood density database (both Zanne et al. 2019 and Chave et al. 2019).

Line 226 : It should read AGWC for consistency with AGWB.

Lines 232-233 and 240-242 : Using the exact date of census or the year only ? In the second case it may introduce huge uncertainty.

Line 236-237 : Please explain rapidly how this correction works.

Line 245 : Avoid using « Note that » in two subsequent sentences.

Lines 246 -247 : The correlation matrix should be rather referred to in the result section.

Line 254 : was not homogenous

Line 258 : was strongly correlated

Line 267 : Why did you use different wood density database for estimating AGB and for computing the mean wood density ? The same database (considered as the most accurate) should be used in both analyses.

Line 271 : Have you considered using the TRY database also ?

Lines 284-286 : I disagree. The focus of this paper is on AGC so that the weight should be BA. Using diameter as weight overemphasize the importance of small trees in AGC dynamics.

Lines 307-309 : Unclear. How can you build these metrics from the mid point ? Please reformulate.

Line 304 : Really impressive botanical identification effort !

Line 320 : Did you consider adding the missing species on the phylogeny based on the litterature ?

Line 332 : Also in Myrs ?

Line 341 : Another option is to use rarefied version of indices, see e.g. the R package Pdcalc (Nipperess and Matsen 2013). Using SES does not represent phylogenetic diversity per se but rather represent whether the community is less or more diverse than expected by chance so it is not a measure of diversity per se but rather a measure of phylogenetic overdispersion/underdispersion.

Line 344 : Why not using PCA components of soil and climate rather than selecting only one variable ?

Line 380 : I feel that this section only provides redundant information.

Line 392 : It is more common to put this para at the end of the statistical section.

Line 409 : I don't understand this sentence.

Line 415 : What is P ?

Line 424 : « whenever possible » Can you be more specific here ?

Line 435 : I disagree, the relationship between diversity and AGC is expected to be causal according to the complementary or selection effects.

Line 443-446 : The use of interaction in the model should be explained. Why do you expect interactions ? It results in a huge number of parameters to estimate (if I well counted them at least 25 parameters) compared to the rather low number of statistical observations (n=49* by the number of recensuses) so that each interaction should have clear justifications and a more parcimonious model should probably be adopted.

Lines 470-471 : Unclear. How did you ensure at least 10 observations per predictor given that they are continuous predictors ?

Line 488 : Please revise figure numbering.

Figure 2 and 3 looks highly redundant in their information. You may consider to move one of these two in the SI.

Line 554-556 : It looks like a tautology.

Line 565-567 : Do you find any evidence of an increase in functional homogenity along your climate gradient to support this statement?

What does bring Figure S3 compare to S2 ?

Line 571 : Why especially rare species ? I may have missed something but is there any evidence of this in your result ? In fact we can make the opposite hypothesis : some species that are currently rare because not in their optimal niche may find more suitable conditions in the future with climate change and may thus increase in abundance.

Line 606 : But according to Figure 4, the correlation between C loss and gain is quite low and deemly significant only thank to a few observations(n=4).

Line 622 : Tucker et al. is inappropriately cited here. It should be mentioned once in the mat & met section only.

Line 632-635 : Sampling effect is likely to be better represented by PD than by MNTD (see e.g. Tucker et al. interpretation of phylogenetic indices).

Line 653-654 : Consider rewording this sentence.

Line 680-690 : Not really convincing in my opinion.

Reviewer #3

(Remarks to the Author)
This study uses data from 49 inventory plots (in 4 clumps) in S/SE Brazil, where forests are relatively under-studied, to understand how the C balance of these forests is changing, and how this is governed by tree diversity and functional trait composition. They find that C has been declining, particularly in the drier sites, which is consistent with an earlier result using 32 of these sites (Maia et al. 2020, Science Advances). Novel results include recent (since ~2010 or earlier) declines in various metrics of species diversity and functional trait (wood density, specific leaf area) dispersion. Other results appear to be more tenuous.

The topic is important, and the authors' work monitoring these forests is extremely valuable. I commend the authors on the importance and ambitious scope of this paper. I do, however, have some concerns:

A lot of the results that are discussed as significant seem tenuous. The analyses examine many relationships, and thus we'd expect some relationships with $p<0.05$ just by chance. In addition, some relationships that appear to be driven by outliers (e.g., Figs. 6, 9a) and/or marginally miss the $p=0.05$ threshold are discussed as significant (and highlighted in the abstract). I'd recommend careful examination to distinguish truly robust relationships from those that are tenuous and context dependent. A more focused presentation highlighting only the fully significant and consistent results will also improve the presentation (see comment below).

The presentation needs to be made far more focused and concise to be suitable for Nature Communications. There are 11 display items (10 figures + 1 table), and the text is generally long and at times repetitive. Many analyses are presented, including several variations of the same basic analysis (e.g., absolute and relative change in C stocks, GAM and full models). While running these various models is unquestionably valuable, the presentation would be improved by focusing just on the versions that the authors consider most important, with others relegated to supporting information. Similarly, the methods section presents the process by which the authors defined the analyses, which is valuable but superfluous if word count is an issue.

Specific comments:

l. 48-52- The results highlighted here are tenuous and context dependent, and are unlikely to represent robust causal relationships.
l.77- "Brazilian neglected non-Amazon forests" – the wording here sounds funny. Suggested re-wording: "Brazilian forests outside the Amazon, which receive comparably little research attention". Also, giving statistics on their area would be informative to readers and help illustrate their importance.
l. 92- Reword to "net productivity is positively correlated with temperature at the global scale" (because I don't think many people expect warming to increase productivity). Schuur reference is missing from citations. A more recent reference here is Banbury Morgan et al. 2021 (https://doi.org/10.1111/gcb.15574)
l. 182-185- It would be helpful to specify as much as possible about stand age, as temporal trends may be attributable to either succession following disturbance or global change. This possibility is discussed as unlikely in the discussion (l. 615-617), but that should be clarified earlier in the manuscript, and more info given on the confidence in aging these forests.

l. 242, 245 (and elsewhere)- You can drop “Note that…”
l. 256- Are all the diversity metrics (e.g., exponential of Shannon’s extropy index) scale-independent?
l. 265- The approach for assigning traits to species (using species-specific values when available, otherwise assigning values from the same genus or family) follows common practice in the field and is the only feasible approach in most cases, but traits can vary significantly within genera and even more so within families. The implication is that the metrics of trait diversity analyzed here are not very reliable.
l. 270, 274- Does “field collections” refer to collections made at your sites?
l. 352, 357 – This means CWD is calculated from one variable that is downscaled (PRE) and one that is not (PET), making a strange compilation that is likely to be particularly biased at high-elevation sites (where PET is probably overestimated). It’s not clear how large this bias would be, but I’d like to have some assurance that it is not large enough to bias the analyses overall. CWD was the variable used in the analyses because it shows better correlation with C variables (l. 365). Would results be consistent if other variables were used?
l. 386- Change in aboveground biomass stock is not equivalent to the net C sink because it accounts only for live biomass, not deadwood, soils, etc.
l. 497-498, Fig. 6- These relationships (between changes in taxonomic diversity and changes in C stocks) appear to be driven by outliers, without which there would be no correlation.
l. 499-500- Example of a repetitive sentence that could be cut to make the manuscript more concise.
l. 508-510- I’m not sure how trustworthy this result is, given my concern about the CWD variable.
l. 517, Fig. 9a- This relationship (between C stocks and sMNTD) appears to be driven by outliers.
l. 518-522 – This result (just barely) misses the p=0.05 significance cutoff, so I’d probably drop it.
l. 519-521- This could be attributable to the tendency for both species diversity and C stocks to increase as stands age, leading to the null expectation that older stands would have higher diversity and lower C sequestration rates. How certain is it that all of these forests are mature/ not recovering from significant past disturbances?
l. 523, 528 (and check elsewhere) – change “negative effect” to “negative correlation” (correlation is not causation)
l. 530-539 – Some of the results, including these, seem more likely to be statistical artifacts (detected because of large number of variables included) than causal, biologically meaningful relationships.
l. 645- do you mean competition-driven mortality? This relationship falls short of significant, and I don’t see that it makes sense biologically, as competition occurs between individuals, not species.
Fig. 4 – Make the figure stand-alone by explaining the abbreviations for the various metrics of P. diversity

Version 1:

Reviewer comments:

Reviewer #1

(Remarks to the Author)
Dear authors,

Thank you for your thoughtful review and for addressing my comments/concerns.
You have done a great job and I have not further comments now beyond of checking some grammatical errors that you have across the manuscript.

(Remarks on code availability)

Reviewer #4

(Remarks to the Author)
Thanks for giving an opportunity to review this revised manuscript. The authors have revised the manuscript based on the comments raised by three reviewers. Meanwhile, the concerns from reviewers are addressed. In general, the manuscript presents interesting findings regarding changes in carbon stocks, carbon sinks, and tree species diversity in tropical and subtropical Brazilian forests, using long-term plot census data. The relationship between carbon stocks, carbon sinks, tree species diversity, and various climatic and soil factors has been thoroughly examined. However, the manuscript contains several language and grammatical errors in its current form. As such, it would be suitable for publication only after careful and thorough language editing.

Line 3: change the title to “Temporal trends of carbon stock, sink, and tree species diversity in tropical and subtropical Brazilian forests”.

Line 50: change “Carbon-diversity” to “Carbon-diversity relationship”.

Line 55: replace “uptake” with “sequestration”.

Line 57: add a full stop before “To fully”.

Line 60: add a full stop “In the” and replace “=” with “some”.

Line 64: add a full stop at the end of the sentence.

Line 66: “oscillating and/or extreme climate events” is ambiguous, please rephrase it.

Line 67, 69: add full stop after the sentence. Please check the entire manuscript, many sentences did not have full stop.

Line 88: delete “()”.

Line 136: delete the sentence “See correlation matrix of carbon dynamics variables in Figure S4”.

Line 169: what does “->” mean?

Line 347: change “showed” to “show”.

Line 432: please indicate if the growth of each surviving tree was calculated using the measurement of DBH for each tree at plot investigation time.

Line 632-637: it is difficult to understand.

(Remarks on code availability)

Reviewer #5

(Remarks to the Author)
I was brought on to follow up on Reviewer 2 and their initial response, and I have focused on their concerns as instructed. Thank you for the opportunity to review the manuscript by Maia et al. It is an impressive resurvey effort, in an important and less studied region. They have also done an incredible amount of work on identification and basic data processing over time. It is a valuable dataset. However, their conclusions are based on statistics which are equivocal in supporting their conclusions, due in large part to the design of the plots and lack of data to test alternative explanations. Further the statistics are not clearly presented and make for tenuous connections to their conclusions. At the least these alternative hypotheses should be clearly and distinctly described in the main manuscript as untested alternative hypotheses.

Discussion of disturbance:
This is not well addressed. The authors respond that it is addressed in the discussion, but it has not been addressed. In fact the concept of disturbance only arises once, noting that the sites are protected from anthropogenic disturbance. The methods note the sites are > 50 years old, which is hardly old enough to be considered stable with respect to “advanced” post-disturbance successional processes (line 386) without additional justification. Windthrow is also not mentioned, which is a potential important disturbance agent in these forests. This is a concern because the patterns observed could reasonably be attributed to typical post-disturbance community assembly processes (homogenization, slowing of C stock gain, wood density trends, etc) and not necessarily the soil chemistry variables and other factors offered as explanatory factors. They could merely be correlates, especially with the relatively small sample size and flexible statistical methods utilized. The authors apparently do not have tree cores or similar. Perhaps this could be addressed with a diameter – estimated age relationship dataset that would convince the reader the patterns observed are not due to post-disturbance development.

Circular climate and biomass calculations
I concur with the prior reviewer that this is a significant issue. If the plots were well distributed in space this would be less of a concern, but the locations of plots are highly clustered. The authors attempted to respond by arguing that any bias introduced is small, but I confess either I am not understanding their argument clearly or it is not sufficient. In either case, it needs to be clarified.

Modeling framework:
Mixed effect models: The authors response appears appropriate, and I concur it is an adequate used of a mixed model as I understand their use (Line 585). Random effects to address the repeated measures makes sense. I strongly suggest the authors expand on the statistical structure of the mixed effect model in the text, however, as I would be confused without the response to reviewer text on their use of the framework.
Spatial clustering:
I again share the first reviewers concern. The authors argue that because spatial autocorrelation (SAC) was not detected, there is no significant concerns to be had. I disagree – it is an indication of model fit in geographic space, but hardly conclusive evidence that SAC doesn’t matter for the conclusions. Spatial issues arise from design, and cannot always be addressed with statistical methodologies or checked via statistics. I sympathize with the authors, as they did not have control over plot locations for this study, but it is still an issue. One option presented was using a “space” variable to address the clustering. The authors dismiss this, noting that it would also be correlated with climate (“this variable is the synthesis of the space itself, so it would be, in essence, the space.”). However I think they missed the first reviewers point, which was to drop the random effect for the plots and then use the random effect for the cluster. I agree with the authors this would be inappropriate, now that they have clarified their initial use of the random effect structure. But we cannot dismiss the spatial arrangement of clusters. Any number of factors NOT in the model but which ARE correlated in space (like a disturbance event, or a past pest outbreak, or anything else) could EASILY have caused the patterns seen and not be considered. How much variance in their response metrics is explained simply by the cluster locations? Is it comparable to those explained by

climate, for example? They are obviously correlated, so this itself does not say it is NOT climate, but it does suggest it could be something else distributed similarly in space to climate (in the case of just four clustered areas, this could be as simple as a 100 year prior wind disturbance that would not be detected per their methods).
One potential solution (though not ideal, and perhaps not even appropriate, as the authors know their data best) would be to include an interaction term between the site and cluster as a random effect. Perhaps this is incorporated, but the equations and the random effect structure is not clear in the manuscript methods. There are other options. But as it stands, it is difficult to support the authors conclusions without major questions about other factors not considered. I note that reviewer 3 also noted this, with the same response from the authors.
In a later comment, the original reviewer noted that the authors don't really have 49 plots. The authors noted there are 49 data points, aggregated from smaller plots. A separation of 1km in a forest is almost nothing in the context of these explanatory variables. The large distance for some plots noted (>1000km) is not really useful either, since the clusters are the key concern. I agree with the reviewer in the spatial sense, it appears to be primarily four clustered units at the broad scale. Given the many metrics that are correlated with space, it seems difficult to argue the 49 plots represent 49 truly independent observations statistically speaking.

Large number of parameters:
The authors have simplified the equations. However the structural issues still exist as noted above.

Relationships and careful interpretation:
The use of GAM's is highly flexible and always should be interpreted with caution. And why not GAMM's?

Overall the statistics are written up in a very confusing manner. Several places are missing equations (line 633). Alternative hypotheses are not formulated or tested.

The net effect of the lack of clarity and consideration of potential alternative explanations - exemplified by the spatial structure concerns - means the reader is not convinced that the conclusions drawn are valid. Perhaps this could be addressed with simply more language around the limitations, but they are substantial and mostly stem from the underlying design of the plot network and the apparent lack of key covariates needed to dismiss alternative hypotheses for these particular conclusions.

This is not meant to dismiss the value of the plot network nor the amount of work the authors have done. These long-term networks are incredibly important. But they have limitations in what they can tell us, especially when the history of the plot location is not known. It can be easy to come to the incorrect conclusions if key variables are not included (or indeed, if one doesn't know to include those variables because of that lack of history).

(Remarks on code availability)

Version 2:

Reviewer comments:

Reviewer #5

(Remarks to the Author)
I thank the authors for their responses. The significant concerns I had were primarily related to the fundamentals of the study design (outside the authors control) with respect to spatial distribution, disturbance history, and known stand age. With their edits, the authors have done a stronger job outlining the limitations of their results as a result of those fundamental limitations. I know that unmeasured variables in the past can create misinterpretations, and this is especially challenging in datasets like this. It is a fundamental challenge. I think the authors have now done a stronger job explaining the value and the limitations. It is a valuable dataset, and I'm happy to see the manuscript as written.

(Remarks on code availability)

Open Access This Peer Review File is licensed under a Creative Commons Attribution 4.0 International License, which permits use, sharing, adaptation, distribution and reproduction in any medium or format, as long as you give appropriate credit to the original author(s) and the source, provide a link to the Creative Commons license, and indicate if changes were made.
In cases where reviewers are anonymous, credit should be given to 'Anonymous Referee' and the source.
The images or other third party material in this Peer Review File are included in the article's Creative Commons license, unless indicated otherwise in a credit line to the material. If material is not included in the article's Creative Commons license and your intended use is not permitted by statutory regulation or exceeds the permitted use, you will need to obtain permission directly from the copyright holder.
To view a copy of this license, visit https://creativecommons.org/licenses/by/4.0/

REVIEWER COMMENTS

Reviewer #1 (Remarks to the Author):

The authors use a long-term and widespread dataset from south and southeastern Brazil tropical and subtropical forests to investigate the long-term trends in carbon stocks, carbon dynamics and diversity. Besides taxonomic diversity the authors also calculated functional and phylogenetic diversity and the community level trait values for wood density and specific leaf area functional traits. The authors focus on finding out if carbon dynamics of the most diverse forests were more stable than those of the less diverse forests. The paper shows interesting analyses and important results, it is well written, with most methods and results supporting the conclusions of the authors.
Below I describe some concerns that I have for the text across the manuscript, in some cases specifically for some of the results and for some of the statements from the discussion that I do not think are totally supported by the results shown.
Response: Thank you for reviewing our paper and providing your feedback. We appreciate your comments and carefully revised the manuscript incorporating the points you raised.

1) Overall, it seems that there is an excessive number of figures and if this is an issue perhaps the authors could keep the most relevant in the main text such as 2-6, 9,10 and leave the others as supplementary material. Please also include the R codes used to run the analysis as to have a clearer idea of the process and how the results were obtained.
Response: We reduced the number of figures to 7 (maximum allowed is 10). The codes can be found at this link:
https://drive.google.com/drive/folders/157Ge4kh18LN8JEN_bjScmdEfjdX1VhEq?usp=sharing
(note that the vegetation data at individual level was not included because it’s restricted by ForestPlots data policy), after publication the codes will be publicly available in a repository.

1.1) As the authors include data for Deciduous forests, and deciduousness can be see as an adaptation to dry environments, which may have implications for the adaptation of species to a drying climate it would be great if the authors could include 'deciduousness'

as a functional trait in their analysis. Perhaps deciduousness can explain more of the changes, on top of the already explained variance, in the carbon metrics analysed.

Response: We agree that deciduousness is an important predictor, however, despite the fact that we don't have this data, it would be circular in our analyses because the forest types in our study are intrinsically associated with deciduousness (deciduous, semideciduous, evergreen). Therefore, because of the climate clusters related to forest types, deciduousness and climate would become too much confounding. Therefore, we opt to keep climate because deciduousness is a long term response to climate. We will recognize this limitation and include deciduousness aspects in the discussion.

L 153 - 154 - why? communities may be getting more deciduous as a strategy in comparison to acquiring more conservative traits. Perhaps the authors could also discuss this possibility.

Response: We consider this possibility in the answer to the following question (below).

155-157 → But also as an adaptation to lower water availability

Response: Thanks for the input. We agree that the forests could theoretically be moving towards an acquisitive and mostly deciduous composition, but evidence indicates the opposite: in a previous study that encompassed many of the sites that we investigate here, we found that deciduous forests have been the most vulnerable (Maia et al. 2020; Sci. Adv.). To consider your input and these observations, we changed the sentence to the following:

> *"However, it could be possible that these communities have moved towards an acquisitive and mostly deciduous composition, as a response to higher temperatures and drought, but recent evidence points to the opposite direction, showing greater mortality among deciduous forest communities (Maia et al., 2020)."*

160-163 → Unless they were already at their physiological limits of water availability and further droughts may push them towards alternative states.

Response: This is a valid point that we agree should be mentioned. Thus, we changed the end of the introduction's last paragraph to the following:

> *"It is possible that less diverse forests dominated by species with low SLA and high WD (theoretically more tolerant to drought and rising temperatures) have been more stable over time, but it remains unresolved whether these species are currently at their physiological limits and, therefore, would be more vulnerable under changing climatic conditions (Allen et al., 2017; Maia et al., 2020)."*

180 → Exemplifies my points above regarding deciduous trees as method of adaptation to drier conditions.

Response: We incorporate the deciduousness aspects as detailed above.

187 → Please clarify if the "subplots" are the measuring unit of the analysis-thus the 49 plots

Response: We rephrased to:

> *"Spatial sampling spanned from 0.2 to 5 ha (mean of 1.03 ha) per site, totalling 50.4 ha. Sampling was carried out using subplots of 400 m² (in most cases). The design follows the following hierarchy: each of the 49 sites has n subplots, each subplot has ~ 400 m² and the sum of the area of the subplots within each site ranges from 0.2 to 5 ha. The subplots are located mainly in the interior of the sites (...)".*

189-190 → How do you think edge effects could be driving your results? Please also specify the number of plots that are within 100m of the forest edge. Perhaps being at the forest edge has an impact on microclimate conditions and affects community composition and phenological responses? e. g. Nunes, M.H., Camargo, J.L.C., Vincent, G. et al. Forest fragmentation impacts the seasonality of Amazonian evergreen canopies. Nat Commun 13, 917 (2022). https://doi.org/10.1038/s41467-022-28490-7

Response: We agree that edges may have important effects, that's why we added site size as a predictor in the models to control for this potential effect. Because of the subplot sampling the design, which aimed to maximize the covered area homogeneously, we can

expect that smaller the site, higher the edge effects, and also that, the chance of a subplot falling within 100 m of the edge increases. So, we believe that the site area is able to account for the edge effects, especially when considering that it's a control variable and not the main focus of our study. Regarding the count of plots in the edge, for many sites we don't have the coordinates of each subplot, so we cannot calculate the number of plots that are within 100 m of the forest edge accurately, so, site area is our best proxy of edge effect. We will added further explanation on this topic in the methods section:
We added this in the study design section in the methods: "The size of the sites ranges from 3.4 ha to 1179 ha (mean 112 ha)."

and added this section in the methods:

> *"Controlling for edge effects*
>
> *Because of the potential edge effects that can influence carbon dynamics we included site area in the models (details in the statistical analysis section) to control its effects in the carbon variables. The edge effects can influence vegetation mainly through changes in microclimatic conditions, affecting composition, phenology, structure and growth (Magnago et al. 2015; Nunes et al. 2022). Because of our sampling the design, which aimed to maximise the covered area homogeneously, we can expect that smaller the site, higher the edge effects."*

225 → "estimations from"

Response: This sentence was removed from the manuscript.

280-284 → Please provide information about the traits assignation to the genus level and family level as well.

Response: We added this information in the text:

> *"The representativity of WDi at genus and family levels were 27.3% at genus level and 10.5% at family level, while for SLAi the representativity was representativity 16.14% at genus level and 5.6% at family level (the distribution of the representativity of species with species-level traits among censuses, family, genus, species and overall are presented in supplementary figures (S6 and S7)."*

301-302 → While using a matrix of species abundance as input is feasible such data may not relate directly to the DBH or Basal area of the individuals in the plot, which greatly also relates to the competition between individuals and their functional composition. Therefore, I would advice to rerun the functional diversity analyses using the commutative basal area of the species, something that would also relate more with how the calculations for the other metrics were made.

Response: We reran all analyses using basal area instead of DBH.

332-333 → Same than above in line 301-302

Response: Same than above.

3) Climate Data

361-362 → please provide the reasoning for defining the climate of a plot for a given census by the 5 preceding years, up to now it seems subjective.

Response: It's because the average census interval length, which is used in the years within the monitoring time except the first census, is 5 years. Therefore we used this value to match the length of the census intervals, it's empirical and not expected to affect the results since the analyses are done in large scale.

194-163 → Introduction, at the end: The authors only seem to make the argument for the impact of climate but do not mention the impact of soil conditions in the diversity, carbon stocks and dy-namics. However, soil data is presented in the methods and used in analysis and results, something with which I agree. Therefore, it would be great if the authors could include some information about the role of soil in the introduction section besides the short paragraph in lines 95 to 98.

Response: We agree that some arguing on the effect of soil on productivity were lacking from our introduction, so we added more information on the interacting effects of soil and climate, along with additional references, to the second paragraph of the introduction.

119- 129 → I do not totally agree with this sentence. Grime 1998 does not suggest this only for low diversity ecosystems. In high diversity ecosystems the most abundant species tend to have more weight in the functioning of the system. Thus I wou-ld

rephrase this sentence account-ing for this observation. Eg. Van der Sande, Masha T., et al. "Old-growth Neotropical forests are shifting in species and trait composition." Ecological Monographs 86.2 (2016): 228-243.

Response: Thank you for pointing that out. Aiming to avoid implying that mass-ratio effects only refer to low-diversity ecosystems, we rephrased the sentence into the following:

> *"In contrast, in less diverse communities where niche complementarity effects are minimal, the functional composition of the most abundant species tends to better predict community productivity (i.e., mass-ratio effects, sensu Grime 1998). Despite its role in high-diversity communities (van der Sande et al. 2017), mass-ratio effects have greater weight in low-diversity communities, reflecting the optimal traits that promote growth and survival in a particular habitat (Prado-Júnior et al. 2016)."*

135 → It would be great to see other references than that of Prado-Junior et al. 2016 when argumenting about the role of plant traits and the relation to carbon stock and the other questions addressed. There is much more literature, including recent one, about this out there.

Response: Thank you for your suggestion, we added the following recent references on the subject:

https://www.sciencedirect.com/science/article/abs/pii/S0048969720367693
https://onlinelibrary.wiley.com/doi/full/10.1111/gcb.15982
https://www.nature.com/articles/s41559-022-01747-6

136-140 → Please also discuss the role of deciduousness here, as this may change the way how plant species behave in such environments in comparison to other acquisitive ones with similar characteristics but not being deciduous

Response: We included discussion on the role of deciduousness among acquisitive species in lines in the revised manuscript.

"Although acquisitive species may be vulnerable to hydraulic failure, stem breakage and herbivory (Reich et al. 2003; Wright et al. 2010; Prado-Júnior et al. 2016;

van der Sande et al. 2017), many are able to endure high temperatures and low humidity by shedding their leaves during the dry season (i.e., deciduousness), particularly in nutrient-rich environments (Oliveira et al. 2021). Such adaptation could theoretically increase the survival of acquisitive deciduous species under a hotter and drier climate, but recent evidence indicates that tropical deciduous forests have had higher tree mortality than wetter forests in recent years (Maia et al., 2020)."

416 → Please specify what are the results for the Moran I analysis and if needed how did the authors accounted for the spatial autocorrelation in the models. If the Moran I test indicates the authors need to account for the spatial autocorrelation and they have not done it then it would be great if the authors can account for this in new analyses.

Response: The Morans' I have not detected spatial autocorrelation, we clarified this in the text.

524-520 → Although I simpatize with the idea of describing the patterns obtained despite the P-values not being significant, in this case the authors are using P-values making figures of model results with no signifi-cant effects which can be misleading. E.g. Figure 10A for WD dispersion by year. Please include slope and P-values also for Figure 10, so the reader can judge the strength and significance of effects.

Response: We added the slopes and P-values in the interaction figure. The confidence interval figures can also be used to judge these effects.

710-712 → But the results from the models do not show significant effects of F. Div. Why are the authors stating this? Either rephrase this specifically stating this effect is not signifi-cant or remove this from the text. Moreover, as far as I can see from the results the F. Div effect was only found for the "relative carbon sink' and not for the other metrics, however, the general text across these lines seems to infer that overall forests with high functional diversity were less resilient, which is also in contrast to other recent analysis. (e.g. Aguirre-Gutierrez et al 2022 Nature Ecol. Evol.)

Response: We agree, however, because F.Div was removed this sentence was removed and we stated that this is context dependent.

"The relationships between carbon dynamics and diversity are context dependent as phylogenetic diversity is linked to higher carbon gains but WD diversity was associated with increasing carbon losses over time."

We also discussed the findings of the Aguirre-Gutierrez et al 2022.

710-712 → Moreover, the authors only used two functional traits to calculate the F. Div. metrics, which hardly may depict the functional composition and diversity of the forests analysis. I would advise on rephrasing the text along these lines (710-712).

Response: The sentence was removed, but we specified that our inferences are about SLA and WD in the next sentence:

"In addition, we found that forests composed of conservative species in WD and SLA are more prone to showing higher productivity."

710 - 712 → Given that the authors only used two functional traits to calculate the F. Div., that they also use the single traits in their models and because most results related to F. Div. are not significant I would strongly advise on leaving this metric out of their analysis for the final version of the manuscript. Again, in my opinion using only two traits hardly represent the functional diversity and strategies tehreon of the forests analysed.

Response: We dropped F. Div and recognized the limitation of only using two traits.

"We also recognize the limitations of the data, especially regarding phylogeny and functional traits, such as using only two traits, and also for the complex models (and less generalizable) we needed to explore the data and test our hypothesis.

708-709 → But WDd, had significant negative effects on Net and Relative C sink, thus this does not seem to support the statement presented across these lines.

Response: We removed the sentence.

Figure 10 →Please include significance levels

Response: Done.

713-714 → Perhaps for SLA for Net C sink only because the results in Figures 7 and Figure 8 do not show any significant effect for WD. Please rephrase the text here and be specific about the findings.

Response: We specified that was for both traits because in the new analyses WD had effects, probably because now we have weighted by basal area instead of DBH.

"In addition, we found that forests composed of conservative species in WD and SLA are more prone to showing higher productivity. "

714-715 → Strange sentence, please rephrase.

Response: We eliminated this sentence because it's not essential and may confuse the readers here.

718-719→ Let the reader decide this, better eliminate this sentence.

Response: Done.

Muito bom manuscrito, foi um prazer ler o estudo.

Jesus Aguirre Gutierrez

Reviewer #2 (Remarks to the Author):

The manuscript by Maia et al. investigates the temporal trend of forest carbon, diversity and composition along a climate gradient in Brazil. The paper is based on a good forest plot dataset with a remarquable effort done on floristic identification, soil measurements and plot remeasurement. It is a sequel of a recent paper led by part of the same group that focused on 32 out of the 49 plots used in the present study but, here, the main originality is to bring diversity in the analyses. I congratulate the authors for this effort. The results are interesting and of wide interest but need to be better statistically assessed in my opinion. The paper is relatively well written even if not always easy to follow, especially in the material and method and results sections due to the large number of analyses done

and a lack of justification for some of the analyses as pointed out in my specific comments below. My overall assessment is positive but here is below some major concerns and several specific ones (that should not be always considered as minor).

Response: Thank you for taking the time to review our paper and for your valuable comments. Your feedback certainly improved the manuscript.

Major concerns

There is little discussion and evidence of the absence of historical disturbances in the studied plots. I know that this kind of information is hard to obtain but given their importance in the studied topics, it should, in my opinion, be better discussed. Speaking of that, the diameter distribution shown in Figure S2 suggests that several sites suffer from a striking lack of tree regeneration (e.g. LAG_07, LUM_01). How can it be explained ?

Response: We discussed this topic, see lines/paragraphs in the discussion

The analysis on the impact of climate on AGC stocks is circular and likely to be impacted by AGB calculation methodology. The authors use the Chave E parameter to model differences in the height-diameter relationship among plots. This E parameter is derived from climatic data so that AGC estimates at least partly derived from climatic data that are then used to predict AGC… Furthermore, the use of several AGB equations, or their mixing, may introduce significant bias between groups of sites located along the climate gradient. The best solution would be to apply the same biomass equation (Chave Eq4 for instance) using site-specific height-diameter allometries. If local tree height data are not available, regional ones, such as the Feldpausch equation, would be, in my opinion, better conceptually.

Response: We agree that this kind of variable may introduce biases. However, this bias is probably small since indeed the height correlates with climate in the tropical region. In our specific case, which we don't have stem level data for many censuses, only the DBHq, using the height equation would introduce a large bias for these censuses since the input would not be the DBH but the DBHq of tree stems. Moreover, using height for some censuses and E for other censuses would prevent us from comparing the biomass because of the DBHq bias. Therefore, using an equation which estimates height only using climate

as a predictor, would introduce a smaller bias than the scenarios above, since all censuses are under the same rule, as the variance in the predictions would be smaller. However, we investigated if indeed the height equation is less correlated with climate. Specific equations from local data are not available as far as we know, so we computed the biomass using the estimated height from the Feldpausch equation. We regressed the individual level biomass as a function of the PCA climate component and found that the R2 of the model containing the biomass estimated using height was larger than the model using the Chave E parameter. The biomass using E and the biomass using height are 98% correlated. The equation using height estimated, on average, larger biomass values (probably because of the DBHq bias). Therefore, we considered that the potential biases related to the E Chave parameter are too small to affect any result and that the side effects of using height equation would be worse than the potential climate bias in our case.

```
>
> r2_heigh > r2_stress
[1] TRUE
> cor(vegetation_data$agwb_h, vegetation_data$agwb)
[1] 0.9877119
> mean(vegetation_data$agwb_h)
[1] 0.1843823
> mean(vegetation_data$agwb)
[1] 0.1588419
>
```

My most important concern is about the way the models have been implemented. I do have at least 4 major concerns here :

1) Because the plot is the statistical unit, implementing a random effect on it is stricly equivalent to implementing a fixed plot effect. It results on a model with no degree of freedom, which may lead to wrong inferences.

Response: We understand your concern, however, let me clarify what we did and why:

In our study design we have 49 sites (which is our random intercept/effect), so we have a random intercept for each of the 49 sites (at this point, the subplots were already aggregated). In each site we have *n* censuses over time, so we have repeated observations within site (samples nested within sites). For example, in the site 1, we have censuses in

2008, 2011, 2017, therefore, for this site we have 3 samples in the biomass data, one for each year, and two observations in the dynamics data, one for each census interval (interval 2008-2011, interval 2011-2017), so, in both cases we have samples nested within sites. These samples are assumed to be non-independent between them since they are nested within the same site, they are pseudoreplicates. Because most sites have more than one sample, which are not independent between them, we need to take this non-independence into account. The most common approach to deal with this design is to use mixed effects models, where the nesting structure (the group where samples are nested, in our case is site) is treated as a random effect. The random effects parameter is the variance in the intercepts, which because it's only one parameter, only counts as 1 degree of freedom, therefore, treating site as random effect requires only one degree of freedom. Moreover, the fact that some sites have only one sample in the dynamics data (all sites have at least two censuses, so these ones will have only one census interval) is usually not a problem for the random effects estimation (the effect is that the variance around their conditional means/BLUPs are large, but not affect the variance parameter of the random effects). It would be a problem if we want to predict the BLUPs (conditional mean/modes) for the sites with only one sample, which is not our case since we are using the random effect only to deal with the non-independence between samples nested within sites. Moreover, observation level random effects can be used even when there is no grouping factor, and each observation is treated as a random effect to reduce variance (see https://peerj.com/articles/616/, for example).

You can also check:
https://www.sciencedirect.com/science/article/abs/pii/S0169534709000196 , the famous Bolker paper, and his very useful FAQ: https://bbolker.github.io/mixedmodels-misc/glmmFAQ.html#should-i-treat-factor-xxx-as-fixed-or-random

The vignette of the lme4 package uses a example similar to our case, subjects (individuals) are treated as random effects because data was collected on each subject along days, so they have non-independent samples nested within individuals over time: https://cran.r-project.org/web/packages/lme4/vignettes/lmer.pdf

One of the limitations is when the number of levels of the random factor (number of sites in our case) is lower than 20, which is not our case (we have 49). However, when the

random effects are only to control the non-independence in the data and not to predict BLUPs, it's better to treat the group as random than fixed in most cases, even if there are less than 20 levels..

Moreover, fixed effects are sensitive to groups with fewer or single samples, and random effects do well on this.

See other papers with a similar design which used the same approach with random effects:

https://www.nature.com/articles/s41586-020-2035-0
https://www.nature.com/articles/nature14283
https://www.science.org/doi/10.1126/sciadv.abd4548

When using random effects only to deal with sampling design, it's not an issue to have random effects with zero variance, since it's the same as a linear model, but at the cost of one degree of freedom to estimate the random effect variance parameter. Thus, it's safer to stick with the random effects, since it costs only 1 df.

2) According to the map of the study sites, the forest plots are not well distributed along the climatic gradient but spatially clustered in four locations. Thus, spatial autocorrelation in climate, soil, forest composition, etc. is likely very high and probably strongly impacted the model. Note that assessing the autocorrelation in the residuals does not control for the influence of this spatial autocorrelation because spatial components have been probably captured in the model.

One potential solution to solve problems 1 and 2 is to use the location (n=4) as a random effect (rather than the plots themselve), to account for the non-independence of plots located in the same location and thus adress the autocorrelation issue.
Response: We agree that there are potential autocorrelations in these variables. However this autocorrelation is a problem when spatial autocorrelation (SAC) was not detected in model residuals. Spatial autocorrelation only invalidates the model inferences when it's not captured by the model predictors, and remains detectable in the model residuals.

This paper (https://link.springer.com/article/10.1186/s41610-019-0118-3), for example, has a concise statement on this: "residual SAC indicates the amount of SAC in the variance which is not explained by explanatory variables".

For example, in the famous Dormann paper on SAC (https://onlinelibrary.wiley.com/doi/10.1111/j.2007.0906-7590.05171.x).
The first sentence of the abstract states:
"Species distributional or trait data based on range map (extent-of-occurrence) or atlas survey data often display spatial autocorrelation, i.e. locations close to each other exhibit more similar values than those further apart. If this pattern remains present in the residuals of a statistical model based on such data, one of the key assumptions of standard statistical analyses, that residuals are independent and identically distributed (i.i.d), is violated. The violation of the assumption of i.i.d. residuals may bias parameter estimates and can increase type I error rates (falsely rejecting the null hypothesis of no effect)."

Therefore, SAC is not an issue in our models since SAC was not detected in the residuals of any model. The autocorrelation in the predictors does not affect our models because no inferences are being made on predictors, and as I said above, if the predictors capture the SAC the model will be less prone to have SAC in residuals, since the predictors explained the variance.

More conceptually, we expect that the predictors affect the response variables (e.g climate affects biomass), we expect that, if climate is clustered in space, the values of the response variables will also be clustered. For example, in a region with drier climate the biomass values will be lower there. So if the model has the climate variable, the biomass predicted values in this region will be lower, that's why the model would be absent of SAC in residuals, since the clustering values of biomass are explained by the clustering values of climate. Therefore SAC would be an issue if climate is not the model and then, the explanation of the clustering values of biomass is lacking, yielding to SAC in the residuals.

We can also say that the correlation in the predictors is part of the phenomena and must be part of the model, therefore the correlation in predictors is not a problem.

See this paper for example:

"*Residual* spatial autocorrelation is what is usually meant by "spatial autocorrelation" and denotes the situation where the residuals of a model are spatially structured *after* covariate effects have been accounted for. When modelling species distributions, the presence of such (residual) spatial autocorrelation is often an indication that an important covariate was not included in the model or that the model was mis-specified in some other way."

https://besjournals.onlinelibrary.wiley.com/doi/full/10.1111/2041-210X.12983

Moreover, if SAC was detected in model residuals, adding an autocorrelation structure would be a choice, however, when no SAC is detected adding an autocorrelation structure would have no effect because if the SAC is absent in residuals the parameters of the autocorrelation structure would be ~ 0 and it would cost degrees of freedom and increase model complexity.

The approach that the reviewer suggest have two problems:

1) it would be hard to find a *n* value where each site belongs to only one group, which makes it impossible to use this approach.
2) Conceptually if you add the group variable as predictor or random effect (in this case, it doesn't matter if it's random or fixed) this variable is the synthesis of the space itself, so it would be, in essence, the space. However, when the reviewer argues that the predictors are autocorrelated/clustered in space, this variable group wouldn't be either? For example, soil is clustered in space and forests with more fertile soils have greater biomass. So, if soil is a predictor, the forests in the fertile soils clusters will have larger biomass predictions values. Then, if a created group falls in a region with larger biomass and this group is a predictor (fixed or random) forests in this group will be also predicted to have larger greater biomass. Although the potential autocorrelation in the model's predictors is not a problem, this approach would not have any effect in this aspect, since it would be just another predictor, which is spatially clustered too. Although I stated above that this approach is impossible in our case, it would be an option if SAC remained in residuals and another predictor (or a spatial predictor, such as lat long in this case) would be useful to remove SAC from the residuals. So, adding this spatial variable

when there is no SAC in residuals, it's effect would be zero, or absorb the effects of the other predictors that the groups may match randomly.

However, the points I discussed above are related to technical aspects of the model but not related to the data. Our models are reliable with the data we have, but the data is indeed clustered and it limits the broadness of the inferences, so we acknowledged the limitations of our data in our inferences.

For example, in the last paragraph of the discussion we added:

"We recognise the limitations of the data, especially related to the phylogenetic and functional variables and the application of complex (and less generalisable) models for data exploration and hypothesis testing."

3) The models were implemented with a huge number of parameters due to the inclusion of several predictors and interactions. The problem is that the number of statistical units does not allow to account for such a large number of parameters to estimate. I even can hardly imagine how models can be succesfully fitted given that plot identity was also considered as a random effect (see point 1). I believe that this may have resulted in overfitting issues that, in turn, may have generated difficult to interpret results (e.g. the fact that WD and SLA dispersion provided opposite result to that obtain with functional diversity on the long term stability for instance). The consequence is that the authors have to find a « plausible » explanation for each of the (sometimes contradictory) results and that, at the end, it is quite hard to get a take home message.

Response: We agree that our models are too complex and we substantially reformulate it, so we removed predictors, interactions and the random slope of year within site (see methods section). However, as discussed above, this issue has no relationship with the random effects. As I said, we have the random intercepts of sites, which are needed to account for the non-independence of the samples nested within sites and it's just 1 parameter, which is not related to overfitting of the fixed effects as it would be if we are using the BLUPs.

4) Few plot measurements were available before 2000 so that some of the relationships should be carefully interpreted (e.g. Fig. 4A, 4E, 4F, 5B) as it appears that only a few observations (n<4) significantly drove the functional form of the predictions. Similarly,

several significant results seem to be the result of a few observations only, rather than a general trend (e.g. Fig. 6 and Fig. 9A). The authors should at least acknowledge this and carefully check those peculiar observations.

Response: We agree with the reviewer and have acknowledged this issue. However, evaluating the presence of outliers must not be checked in the x y plots, but in the residuals vs fitted plots, which we have not detected extreme residuals. It's because the model fit for a given predictor take into account the explanation of the other predictors (and therefore, the variance reduction), therefore, when you look the fitted curve of y and a given predictor with the observed data, some points can appear far from the curve, but they are not necessarily extreme values or have impacted the fit.

Finally, given the topic of the paper, I was surprise not to see any discussion on the carbon residence time.

Response: We agree, so we added the carbon residence time in our analyses and tested the effect of all predictors on it, such as we did for the other carbon variables.

Specific comments

Lines 48-49 : « context-dependent » seems inappropriate here as the sentence only suggests that the results depend on the biodiversity metrics used.

Response: We removed this sentence from the abstract.

Line 71 : I would replace « ecological metrics » by « ecological dimensions » here.

Response: Thank you for the suggestion. We replaced "ecological metrics" with "ecological dimensions".

Line 76 : idem or even better, I would simply say : « However, it is largely unknown how diversity affects…. » to better link with the previous sentence. BTW, I am not a native english speaker but « Brazilian neglected non-Amazon forests » sounds weird to me (if ok don't worry about this comment).

Response: We rephrased this part of the introduction entirely, attempting to accommodate reviewers 2 and 3's suggestions:

"In the Amazon, recent studies have found a positive effect of diversity in forest resilience to climate change (Poorter et al. 2017; Aleixo et al. 2019; Coelho de Souza et al. 2019; McDowell et al. 2020). However, these relationships have been little explored on a comprehensive scale in other Brazilian forests, such as the Atlantic Forest (157,200 km2; Ribeiro et al. 2009) and Caatinga dry forests (850,000 km2; Queiroz 2006), which experience lower water availability and different trait-productivity relationships (Prado Junior et al. 2016)."

Line 79 : I would rather say « with functional trade offs more associated…. »

Response: In rephrasing the paragraph, we removed this sentence.

Line 80-83 : Please reformulate : insights cannot be crucial to understand.

Response: In rephrasing the paragraph, we removed this sentence.

Line 109 : What do you mean by « temporal complementarity» ? Also, please consider mentioning the « Insurance hypothesis » by Yachi & Loreau 1999 PNAS.

Response: Thank you for this suggestion. We rephrased most of the paragraph and added the following, mentioning the insurance hypothesis:

"Diverse communities are expected to have higher resource-use efficiency due to niche complementarity effects (Tilman 1999; Díaz et al. 2011; Loreau and de Mazancourt 2013; Prado-Júnior et al. 2016) which, over time and under fluctuating environmental conditions, result in stable carbon dynamics (i.e., 'insurance hypothesis') (Yachi and Loreau 1999)."

Line 120 : Can we really simply summarize the mass ratio hypothesis to « functional composition » ?

Response: We rephrased the entire paragraph to contain the original essence we tried to convey, attempting to leave little room for misleading interpretations. Now, the part on mass-ratio and functional composition looks like the following:

"In contrast, in less diverse communities where niche complementarity effects are minimal, the functional composition of the most abundant species tends to better predict community productivity (i.e., mass-ratio effects, sensu Grime 1998).

Despite its role in high-diversity communities (e.g., van der Sande et al. 2017), mass-ratio effects have greater weight in low-diversity communities, reflecting the optimal traits that promote growth and survival in a particular habitat (Prado-Júnior et al. 2016).”

line 149 : Please change to « long-term dataset of 49 forest plots ». You do not have really 49 sites according to the map or if you want to keep site everywhere in the ms, you should provide information on the minimum distance between the studied plots.

Response: Our dataset was indeed composed of 49 sites. In each site, we had a number of subplots (mostly of 400 m²) that totalled 0.2 to 5 ha (mean = 1.03 ha), as detailed in the methods. We rephrased line 149 of the original manuscript into: *“Here, we use an extensive and unique long-term dataset of 49 forest plots in south and southeastern Brazil.”*. In the methods, we also added the information of the minimal distance between sites, as follows: “The minimum distance between sites is ~1 km and the maximum distances are > 1000 km.”

Line 153 : « ecological factors » is inappropriate here. Biotic conditions or forest composition would be more appropriated for instance.

Response: We replaced “ecological factors” with “biotic conditions”.

Lines 154-155 : It would be better to have a proper expectation here. For instance, if you hypothesize that the forest will be more dominated by species with conservative strategies, it should in turn decrease the C productivity.

Response: We thank the reviewer for their input. We changed this paragraph to highlight our expectation (i.e., decreasing carbon productivity) and to better describe alternative scenarios.

Lines 188-190 : Not sure to understand this sentence.

Response: We rephrased to:

“Spatial sampling spanned from 0.2 to 5 ha (mean of 1.03 ha) per site, totalling 50.4 ha. Sampling was carried out using subplots of 400 m² (in most cases). The design follows the following hierarchy: each of the 49 sites has n subplots, each

subplot has ~ 400 m² and the sum of the area of the subplots within each site ranges from 0.2 to 5 ha. The subplots, which are located mainly in the interior of the sites, (...).”

Line 191-192 : How can you estimate C dynamics with only one year census ?!

Response: We can’t. All sites have at least 2 census (1 census interval).

The text states this: “The number of censuses per site ranges from 2 to 8 (mean 3)...”.

Line 194 : Change to : « hosted in the ForestPlots.net platform »

Response: Done.

Line 200 : This is not a quadratic mean. To get a mean you should divide the quadratic sum by the number of stems, but it is not what you want here so please just reformulate the wording.

Response: Thanks, we fixed it in the manuscript.

Line 215 : Provide references for the global wood density database (both Zanne et al. 2019 and Chave et al. 2019).

Response: Done.

Line 226 : It should read AGWC for consistency with AGWB.

Response: Done.

Lines 232-233 and 240-242 : Using the exact date of census or the year only ? In the second case it may introduce huge uncertainty.

Response: We reran all analyses using the exact date.

Line 236-237 : Please explain rapidly how this correction works.

Response: Done.

Line 245 : Avoid using « Note that » in two subsequent sentences.

Response: We corrected this along the text.

Lines 246 -247 : The correlation matrix should be rather referred to in the result section.

Response: Done.

Line 254 : was not homogenous

Response: Done.

Line 258 : was strongly correlated

Response: Done.

Line 267 : Why did you use different wood density database for estimating AGB and for computing the mean wood density ? The same database (considered as the most accurate) should be used in both analyses.

Response: We didn’t use different databases for AGB and CWMs, we used the same This first sentence of the Carbon stocks and dynamics section states:

> *“To calculate tree above-ground woody biomass (AGWB) we obtained wood density (WDi) values from the global wood density database, literature and field collections; more details on traits are presented in the functional traits section.”*

Line 271 : Have you considered using the TRY database also ?

Response: Thank you for your suggestion regarding the use of the TRY database. Concerning wood density, we have incorporated both our measured wood density values and data from the Global Wood Density (GWD) database. This approach provides a more comprehensive source and enables us to filter GWD data collected solely within South America, thereby reducing potential issues related to intraspecific variability arising from diverse environmental conditions. Regarding specific leaf area, our local collected data combined with literature data collected under relatively similar environmental conditions, already encompasses 76% of all species sampled in our censuses and the field collections encompasses 64% of the basal area. This comprehensive coverage suggests that our analysis accounts for a substantial portion of the studies taxa.

Lines 284-286 : I disagree. The focus of this paper is on AGC so that the weight should be BA. Using diameter as weight overemphasize the importance of small trees in AGC dynamics.

Response: We agree, so we reran all analyses using BA instead of DBH.

Lines 307-309 : Unclear. How can you build these metrics from the mid point ? Please reformulate.

Response: The same way we did for the other variables, in the end of taxonomic diversity section in the methods we explained:

> *"We used the Shannon diversity, hereafter called taxonomic diversity, from the mid-point of the interval (i.e., the mean between the value of the start of the census interval and the value of the end of the interval) to represent the variable during the census interval."*

Line 304 : Really impressive botanical identification effort !

Response: Thank you.

Line 320 : Did you consider adding the missing species on the phylogeny based on the litterature ?

Response: We appreciate the reviewers' suggestion regarding the inclusion of missing lineages in our phylogenetic hypothesis. Our decision to use an incomplete phylogeny was primarily due to the absence of a comprehensive molecular phylogeny covering all the tree genera from both Mata Atlantica and Caatinga in our study. While we acknowledge the importance of a complete phylogeny, our dataset's high representativeness helps mitigate the potential drawbacks associated with missing lineages. In fact, our DNA-based phylogenetic hypothesis encompasses 87.66% of all species and 94.2% of the basal area recorded in the censuses. Adding missing lineages to the phylogeny would likely result in numerous unresolved nodes (polytomies) and yield to a phylogenetic tree that is not completely resolved. This, in turn, may lead to an underestimation of phylogenetic diversity. For example, a study by Swenson et al., 2009 demonstrated that while phylogenetic diversities calculated using phylogenies with polytomies generally correlate well with phylogenetic diversity from fully bifurcating phylogenies, various metrics tend to be increasingly underestimated as phylogenies become less resolved. Therefore, we believe that our approach strikes a balance between completeness and phylogenetic accuracy given the available data constraints.

Swenson et al., 2009:

https://journals.plos.org/plosone/article?id=10.1371/journal.pone.0004390

Line 332 : Also in Myrs ?

Response: Yes, it was, but this part was removed from the text because we kept only sesPD.

Line 341 : Another option is to use rarefied version of indices, see e.g. the R package Pdcalc (Nipperess and Matsen 2013). Using SES does not represent phylogenetic diversity per se but rather represent whether the community is less or more diverse than expected by chance so it is not a measure of diversity per se but rather a measure of phylogenetic overdispersion/underdispersion.

Response: thanks for pointing this out, however ses can indeed be used to deal with this issue. For example, in the package reference: (https://search.r-project.org/CRAN/refmans/picante/html/pd.html) it's simply stated:

https://search.r-project.org/CRAN/refmans/picante/html/pd.html   Pesquisar

Warning

If the root is to be included in all calculations (`include.root=TRUE`), the PD of all samples will include the branch length connecting taxa in those samples and the root node of the supplied tree. The root of the supplied tree may not be spanned by any taxa in the sample. If you want the root of your tree to correspond to the most recent ancestor of the taxa actually present in your sample, you should prune the tree before running `pd`:

```
prunedTree <- prune.sample(sample,tree)
```

Note

The data sets need not be species-community data sets but may be any sample data set with an associated phylogeny. PD is not statistically independent of species richness, it positively correlates with species richness across samples. The function `ses.pd` compares observed PD to the values expected under various randomizations and allows a way to standardize for unequal richness across samples.

If the root is to be included in all calculations of PD (`include.root=TRUE`), the tree must be rooted. Single-species samples will be assigned a PD value equal to the distance from the root to the present.

If the root is not included in all calculations by default (`include.root=FALSE`), the tree need not rooted, but in the case of single-species samples the PD will be equal to NA and a warning will be issued.

Author(s)

Matthew Helmus mrhelmus@gmail.com, Jonathan Davies davies@nceas.ucsb.edu, Steven Kembel steve.kembel@gmail.com

References

Faith D.P. (1992) Conservation evaluation and phylogenetic diversity. Biological Conservation, 61, 1-10.

Line 344 : Why not using PCA components of soil and climate rather than selecting only one variable ?

Response: We agree and have used PCA for climate and soil using all variables we have for each.

Line 380 : I feel that this section only provides redundant information.

Response: We agree. This part was removed from the manuscript because these analyses were dropped based on the comments of the reviewer and because it has a smaller fit with the manuscript main ideas, especially because the manuscript is already large and has many results.

Line 392 : It is more common to put this para at the end of the statistical section.

Response: We agree and moved this paragraph to the end of the statistical section.

Line 409 : I don't understand this sentence.

Response: It was because in the temporal changes models, where year is not a predictor, the random slope of year was not included, as it was in the other models. But this sentence was removed both because the temporal changes analyses were dropped and because we opt to keep only the random intercepts in sake of model parsimony.

Line 415 : What is P ?

Response: We referred to soil phosphorus, however, this was no longer needed because we replaced the soil variables with the soil PCA.

Line 424 : « whenever possible » Can you be more specific here ?

Response: We added this explanation:

> *"(for diversity and traits variables we used census level data instead of census-interval data to maximise the use of the available data)."*

Line 435 : I disagree, the relationship between diversity and AGC is expected to be causal according to the complementary or selection effects.

Response: These analyses were removed, as I stated above.

Line 443-446 : The use of interaction in the model should be explained. Why do you expect interactions ? It results in a huge number of parameters to estimate (if I well counted them at least 25 parameters) compared to the rather low number of statistical

observations (n=49* by the number of recensuses) so that each interaction should have clear justifications and a more parcimonious model should probably be adopted.

Response: We substantially reduced the model. See the statistical section in the methods for more details. In summary we stick with only sesPD to represent phylogenetic diversity, dropped RaoQ and carbon stocks (which was a predictor of the dynamics variables). Therefore, only climate, taxonomic and phylogenetic diversity, s, SLA and WD CWMs, and SLA and WD dispersion remained in the interaction. Climate was kept because it's an interaction we know a priori and it's important to control for its effect, and the other variables are the variables we are testing the hypothesis. We also dropped the random slopes of year within sites.

Lines 470-471 : Unclear. How did you ensure at least 10 observations per predictor given that they are continuous predictors ?

Response: If we 100 data points/samples, the model would be limited to 10 predictors, so we have 10 samples for each predictor in the model.

Line 488 : Please revise figure numbering.

Response: Done.

Figure 2 and 3 looks highly redundant in their information. You may consider to move one of these two in the SI.

Response: We opted to keep both figures because the information on relative values is new and now we have CRT, and we are within the maximum number of allowed figures.

Line 554-556 : It looks like a tautology.

Response: This part will be removed because carbon stocks were no longer a predictor.

Line 565-567 : Do you find any evidence of an increase in functional homogenity along your climate gradient to support this statement?

Response: We found evidence of functional homogenization over time (figure 4) but we have not inspected it over space since it's out of our scope, so we just stated this based on the literature we cited. We reformulated to:

"Although the direct drivers of this homogenization are not easy to assess and we have not inspected these aspects, the most recent literature suggests that drought and rising temperatures are likely to be the most important driversones (Greenwood et al. 2017; Gomes et al. 2019; Aguirre-Gutiérrez et al. 2020)"

What does bring Figure S3 compare to S2 ?

Response: It's just a continuous version of S2, which is discrete.

Line 571 : Why especially rare species ? I may have missed something but is there any evidence of this in your result ? In fact we can make the opposite hypothesis : some species that are currently rare because not in their optimal niche may find more suitable conditions in the future with climate change and may thus increase in abundance.

Response: We agree with the reviewer, the opposite hypothesis can be also true. We added this:

"Conversely, certain species, particularly those that are rare because they are not in the optimum environment, might experience benefits from climate change. This could occur in communities where the emerging conditions align more closely with the optimal niche requirements of these species, compared to the pre-existing conditions."

Line 606 : But according to Figure 4, the correlation between C loss and gain is quite low and deemly significant only thank to a few observations(n=4).

Response: Carbon stocks is not a predictor anymore, so this sentence was removed.

Line 622 : Tucker et al. is inappropriately cited here. It should be mentioned once in the mat & met section only.

Response: We agree and dropped this citation, and sMTND is not a predictor anymore too.

Line 632-635 : Sampling effect is likely to be better represented by PD than by MNTD (see e.g. Tucker et al. interpretation of phylogenetic indices).

Response: After the changes, we discussed the phylogenetic niche complementarity in terms of PD and carbon gains, so this part of the discussion was kept but relating it to PD.

Line 653-654 : Consider rewording this sentence.

Response: This sentence was removed as requested by another reviewer.

Line 680-690 : Not really convincing in my opinion.

Response: This sentence was also removed because we dropped the FDiv variable as requested by another reviewer and to simplify the models.

Reviewer #3 (Remarks to the Author):

This study uses data from 49 inventory plots (in 4 clumps) in S/SE Brazil, where forests are relatively under-studied, to understand how the C balance of these forests is changing, and how this is governed by tree diversity and functional trait composition. They find that C has been declining, particularly in the drier sites, which is consistent with an earlier result using 32 of these sites (Maia et al. 2020, Science Advances). Novel results include recent (since ~2010 or earlier) declines in various metrics of species diversity and functional trait (wood density, specific leaf area) dispersion. Other results appear to be more tenuous.

The topic is important, and the authors' work monitoring these forests is extremely valuable. I commend the authors on the importance and ambitious scope of this paper. I do, however, have some concerns:

A lot of the results that are discussed as significant seem tenuous. The analyses examine many relationships, and thus we'd expect some relationships with $p<0.05$ just by chance. In addition, some relationships that appear to be driven by outliers (e.g., Figs. 6, 9a) and/or marginally miss the $p=0.05$ threshold are discussed as significant (and highlighted in the abstract). I'd recommend careful examination to distinguish truly robust relationships from those that are tenuous and context dependent. A more focused presentation highlighting only the fully significant and consistent results will also improve the presentation (see comment below).

The presentation needs to be made far more focused and concise to be suitable for Nature Communications. There are 11 display items (10 figures + 1 table), and the text is generally long and at times repetitive. Many analyses are presented, including several variations of the same basic analysis (e.g., absolute and relative change in C stocks, GAM and full models). While running these various models is unquestionably valuable, the presentation would be improved by focusing just on the versions that the authors consider most important, with others relegated to supporting information. Similarly, the methods section presents the process by which the authors defined the analyses, which is valuable but superfluous if word count is an issue.

Response: Thank you for your constructive comments and advice on how to improve the manuscript, we have taken your suggestions into account and thus have improved the new version of the manuscript.

Specific comments:

l. 48-52- The results highlighted here are tenuous and context dependent, and are unlikely to represent robust causal relationships.

Response: These results were replaced with the results of the new analyses.

l.77- "Brazilian neglected non-Amazon forests" – the wording here sounds funny. Suggested re-wording: "Brazilian forests outside the Amazon, which receive comparably little research attention". Also, giving statistics on their area would be informative to readers and help illustrate their importance.

Response: Thank you for the suggestion. We rephrased it to:

> *"However, these relationships have been little explored on a comprehensive scale in other Brazilian forests, such as the Atlantic Forest (157,200 km2; Ribeiro et al. 2009) and Caatinga dry forests (850,000 km2; Queiroz 2006), which experience lower water availability and different trait-productivity relationships (Prado Junior et al. 2016)."*

l. 92- Reword to "net productivity is positively correlated with temperature at the global scale" (because I don't think many people expect warming to increase productivity).

Schuur reference is missing from citations. A more recent reference here is Banbury Morgan et al. 2021 (https://doi.org/10.1111/gcb.15574)

Response: We have changed the sentence to accommodate the reviewer's suggestion. Thank you for pointing out the missing reference. We replaced it for Banbury Morgan et al. 2021.

l. 182-185- It would be helpful to specify as much as possible about stand age, as temporal trends may be attributable to either succession following disturbance or global change. This possibility is discussed as unlikely in the discussion (l. 615-617), but that should be clarified earlier in the manuscript, and more info given on the confidence in aging these forests.

Response: We agree, and added further explanation on this. However, as stated in the text, the historical record was based on field observations and personal communication with reserve managers, local people and landowners. The most reliable information we can consider is that the forests are likely to have > 50 years. We rephrased to:

> *"The forests are at advanced successional stages and had not suffered the effects of fire, landslides, floods and logging during the monitoring period and before the first census of each site. The historical record was based on field observations and personal communication with reserve managers, local people and landowners. Although we don't have information on the exact age of each forest, based on the records and field observations, all forests are likely to be > 50 years."*

l. 242, 245 (and elsewhere)- You can drop "Note that…"

Response: Done.

l. 256- Are all the diversity metrics (e.g., exponential of Shannon's extropy index) scale-independent?

Response: Yes, they are. They are correlated between them, so we kept only shannon which is correlated with both others. The values of the index between samples are comparable because they are rarefied using 90% of sample completeness (coverage-based rarefaction and extrapolation sampling curves)

l. 265- The approach for assigning traits to species (using species-specific values when available, otherwise assigning values from the same genus or family) follows common practice in the field and is the only feasible approach in most cases, but traits can vary significantly within genera and even more so within families. The implication is that the metrics of trait diversity analyzed here are not very reliable.

Response: We agree. We acknowledged this limitation in the methods section:

"This methodology of using average traits from literature, although largely used because of the practical difficulties of massive trait collection in the field, has a high level of uncertainty, therefore, the traits variables can only be viewed as a rough representation of reality instead of a precise metric."

l. 270, 274- Does "field collections" refer to collections made at your sites?

Response: Yes. We clarified this in the text:

"collections made at some of the sites used in the study".

l. 352, 357 – This means CWD is calculated from one variable that is downscaled (PRE) and one that is not (PET), making a strange compilation that is likely to be particularly biased at high-elevation sites (where PET is probably overestimated). It's not clear how large this bias would be, but I'd like to have some assurance that it is not large enough to bias the analyses overall. CWD was the variable used in the analyses because it shows better correlation with C variables (l. 365). Would results be consistent if other variables were used?

Response: I don't believe that this bias is large because the variables are strongly correlated. However, as suggested by another reviewer we made a PCA of the climate variables. I believe that this approach also deals with the issue you stated above.

l. 386- Change in aboveground biomass stock is not equivalent to the net C sink because it accounts only for live biomass, not deadwood, soils, etc.

Response: This part was removed because these analyses were dropped from the manuscript.

l. 497-498, Fig. 6- These relationships (between changes in taxonomic diversity and changes in C stocks) appear to be driven by outliers, without which there would be no correlation.

Response: As I answered to another reviewer, judging the presence of outliers based on the model fit and the raw observed data is easy to draw wrong conclusions because the fit of a given predictor takes the variance reduction caused by the explanation of the other predictors into account. However, we dropped these analyses because, indeed, they are not too solid and out of the scope of the paper, which is already extensive.

l. 499-500- Example of a repetitive sentence that could be cut to make the manuscript more concise.

Response: This part was removed because these analyses were dropped from the manuscript.

l. 508-510- I'm not sure how trustworthy this result is, given my concern about the CWD variable.

Response: Now the climate is represented by the PCA axis.

l. 517, Fig. 9a- This relationship (between C stocks and sMNTD) appears to be driven by outliers.

Response: As I said, raw data and the fitted curve is not the best way to evaluate outliers. But sMNTD was dropped from the analyses in order to obtain a more parsimonious model.

l. 518-522 – This result (just barely) misses the p=0.05 significance cutoff, so I'd probably drop it.

Response: We agree, but no actions were needed because it has not occurred in the new analyses.

l. 519-521- This could be attributable to the tendency for both species diversity and C stocks to increase as stands age, leading to the null expectation that older stands would have higher diversity and lower C sequestration rates. How certain is it that all of these forests are mature/ not recovering from significant past disturbances?

Response: This result was removed because of the new results.

l. 523, 528 (and check elsewhere) – change “negative effect” to “negative correlation” (correlation is not causation).

Response: We agree and change it over the text. However, instead of “correlation” we used “relationship” since these relationships can be causal too.

l. 530-539 – Some of the results, including these, seem more likely to be statistical artifacts (detected because of large number of variables included) than causal, biologically meaningful relationships.

Response: We agree, so we reduced the model to achieve more parsimony and only considered P values lower than 0.05 in this new version.

l. 645- do you mean competition-driven mortality? This relationship falls short of significant, and I don’t see that it makes sense biologically, as competition occurs between individuals, not species.

Response: We agree, this sentence was removed.

Fig. 4 – Make the figure stand-alone by explaining the abbreviations for the various metrics of P. diversity

Response: Now we have only one variable to represent phylogenetic diversity to reduce model complexity, so there is no need to explain the abbreviations in this version.

REVIEWER COMMENTS

Reviewer #1 (Remarks to the Author):

Dear authors,

Thank you for your thoughtful review and for addressing my comments/concerns. You have done a great job and I have not further comments now beyond of checking some grammatical errors that you have across the manuscript.

Reviewer #4 (Remarks to the Author):

Thanks for giving an opportunity to review this revised manuscript. The authors have revised the manuscript based on the comments raised by three reviewers. Meanwhile, the concerns from reviewers are addressed. In general, the manuscript presents interesting findings regarding changes in carbon stocks, carbon sinks, and tree species diversity in tropical and subtropical Brazilian forests, using long-term plot census data. The relationship between carbon stocks, carbon sinks, tree species diversity, and various climatic and soil factors has been thoroughly examined. However, the manuscript contains several language and grammatical errors in its current form. As such, it would be suitable for publication only after careful and thorough language editing.

Line 3: change the title to “Temporal trends of carbon stock, sink, and tree species diversity in tropical and subtropical Brazilian forests”. Addressed.

Line 50: change “Carbon-diversity” to “Carbon-diversity relationship”. Addressed.

Line 55: replace “uptake” with “sequestration”. Addressed.

Line 57: add a full stop before “To fully”. Addressed.

Line 60: add a full stop “In the” and replace “=” with “some”. Addressed.

Line 64: add a full stop at the end of the sentence. Addressed.

Line 66: “oscillating and/or extreme climate events” is ambiguous, please rephrase it. Addressed.

Line 67, 69: add full stop after the sentence. Please check the entire manuscript, many sentences did not have full stop. Addressed.

Line 88: delete "()". Addressed.

Line 136: delete the sentence "See correlation matrix of carbon dynamics variables in Figure S4".

Line 169: what does "->" mean? The symbol meant to describe the soil PCA axis that was negatively related to phosphorus and positively related to magnesium. We changed this notation to a more descriptive wording throughout the text: phosphorus-to-magnesium instead of phosphorus -> magnesium, as previously.

Line 347: change "showed" to "show". Addressed (line 361).

Line 432: please indicate if the growth of each surviving tree was calculated using the measurement of DBH for each tree at plot investigation time. Addressed (lines 467-469).

Line 632-637: it is difficult to understand. Addressed by several clarifications in the methods section text.

Reviewer #5 (Remarks to the Author):

I was brought on to follow up on Reviewer 2 and their initial response, and I have focused on their concerns as instructed.

Thank you for the opportunity to review the manuscript by Maia et al. It is an impressive resurvey effort, in an important and less studied region. They have also done an incredible amount of work on identification and basic data processing over time. It is a valuable dataset. However, their conclusions are based on statistics which are equivocal in supporting their conclusions, due in large part to the design of the plots and lack of data to test alternative explanations. Further the statistics are not clearly presented and make for tenuous connections to their conclusions. At the least these alternative hypotheses should be clearly and distinctly described in the main manuscript as untested alternative hypotheses.

We appreciate the reviewer's thoughtful feedback and their acknowledgment of the importance of our effort, as well as the extensive work in species identification and data processing. We acknowledge the reviewer's concerns, and below we

analyze, discuss, and present results to address them. We have also followed the reviewer's advice by ensuring that alternative hypotheses are clearly and distinctly described in the main manuscript as untested alternative explanations. Details in the answers below.

Discussion of disturbance:

This is not well addressed. The authors respond that it is addressed in the discussion, but it has not been addressed.

Response: We have now addressed disturbance in the methods, early in the discussion, and also added this caveat in the final remarks.

"The sites were selected to represent forests at advanced successional stages with minimal human intervention. Although we were unable to determine the exact age of each forest, we conservatively estimate that they are likely over 50 years old. Based on field observations and personal communication with reserve managers, local communities and landowners, the sites have not experienced the effects of fire, landslides, floods or logging during or before the monitoring period. Thus, disturbance history and forest age are not expected to be correlated with space and environmental conditions."

"Although disturbance and forest age are expected to be spatially homogeneous in our samples based on the sampling criteria and historical data, successional processes cannot be completely disregarded. Thus, considering that successional processes are controlled in our samples,"

"We also acknowledge the need for precise measures of disturbance history and forest age to better understand the carbon sink dynamics in these regions. However, our sampling strategy was designed to represent the most conserved and least disturbed forests, so disturbance and age are unlikely to confound the observed relationships. This supports the reliability of our findings, although the role of disturbance in these forests warrants further investigation."

In fact the concept of disturbance only arises once, noting that the sites are protected from anthropogenic disturbance. The methods note the sites are > 50 years old, which is hardly old enough to be considered stable with respect to “advanced” post-disturbance successional processes (line 386) without additional justification.

Response: Since the earliest projects, starting in 1987, the selection of forest sites was based on their conservation status, disturbance history, and successional stage. The most well-preserved and mature forest sites in the landscape were chosen to represent the forests with minimal human intervention in the region and almost all sites are under law protection because of their location and/or high conservation status. Therefore, an estimate of > 50 years is conservative, as the forests are likely to be much older. Given that most forests in the surrounding areas have remained less conserved to this day, the sampled areas continue to be among the least disturbed, maintaining their ecological continuity and absence of disturbances.

In addition, the relationships we found with our predictors would only be invalidated if the disturbance level or age were correlated with the predictors, which is likely not the case as, the known forest histories, context, and selection criteria (focusing on the most conserved and mature forests) are homogeneous across regions and samples; therefore, the relationships we found are likely to be valid. Moreover, in the worst-case scenario where the negative carbon sink is caused by post-disturbance processes, our findings remain relevant since our sites represent the most conserved forests in their regions, we can extrapolate that even the most preserved forests are currently likely to be experiencing a negative carbon sink. Therefore, if the observed patterns were driven by disturbance processes, it is highly unlikely that we would find this general negative trend in net carbon sink, as it would require all forest sites to be in the same disturbance phase simultaneously, which is unlikely.

Regarding functional and diversity variables, based on the estimations by Lourens Poorter et al., *Multidimensional tropical forest recovery. Science* 374, 1370-1376 (2021), "recovery to 90% of old-growth values is fastest for plant functioning (<2.5 decades), intermediate for structure and species diversity (2.5 to 6 decades)," the functional and diversity variables would not be significantly biased by successional stage in our case as we estimate that our forests samples are older than this. In addition, the trend in wood density showed a slight increase over time, being almost stable but showed a decline in the most recent years, which is inconsistent with patterns expected during early successional stages.

We added further justification in the text, as showed in the answer above.

Windthrow is also not mentioned, which is a potential important disturbance agent in these forests. This is a concern because the patterns observed could reasonably be attributed to typical post-disturbance community assembly processes (homogenization, slowing of C stock gain, wood density trends, etc) and not necessarily the soil chemistry variables and other factors offered as explanatory factors. They could merely be correlates, especially with the relatively small sample size and flexible statistical methods utilized.

The fragment size predictor, used as a proxy for edge effects, also reflects susceptibility to windthrow, which we will explicitly mention in the text. As noted previously, the disturbance regime is unlikely to be biased by region or sample, making it improbable that correlations between predictors would invalidate the relationships we found. Homogenization effects are mitigated by the age estimates, considering the recovery times proposed by Poorter et al. (2021) (Multidimensional tropical forest recovery). Regarding carbon stocks, successional processes would predict a plateau following the growth phase (note that a positive net carbon sink occur in old-growth forest, see Hubau et al 2020 - *Asynchronous Carbon Sink Saturation in African and Amazonian Tropical Forests*), not the decline we observed. This suggests the influence of an additional factor, such as climate change, supported by our data showing a linear increase in temperature and drought peaks coinciding with the shift from a positive to a negative sink after 2010. As mentioned previously, the trend in wood density was almost stable, with a decline in recent years, which is inconsistent with patterns expected during early successional stages. The sample size is not too small, as our dataset includes four large-scale spatial clusters that effectively represent broad regional patterns. Within each cluster and across regions within the clusters, there are various environmental differences, such as soil and even climate. For example, in one of the spatial clusters, we have data from both semideciduous and evergreen forests. This diversity, combined with the temporal dimension (which increases the sample size and is accounted for by the random factor in the mixed models) allows us to consider our sample as representative of these regions and that the regions represent large patterns. Among the models used, the most flexible are the Generalized Additive Models (GAMs), but these were applied only with time as a predictor, ensuring that model complexity remains controlled. In this context, using GAMs is appropriate for identifying temporal patterns over time.

The authors apparently do not have tree cores or similar. Perhaps this could be addressed with a diameter – estimated age relationship dataset that would convince the reader the patterns observed are not due to post-disturbance development.

As far as we know, there is no age-diameter model for our regions and forest types in the literature. Moreover, using any such equation or estimating age through growth rates would introduce bias, as larger diameters are associated with both higher biomass and older trees. Growth would also be related to carbon gains, thus creating a correlation between diameter, growth, biomass, and carbon sink. This would leak information across variables, causing age to be intrinsically correlated with our variables of interest and leading to bias in results. Therefore, considering that diameter and growth are influenced by various factors such as climate, climate change, soil, evolutionary history, and functional traits, age estimation without taking this into consideration would be highly biased.

Circular climate and biomass calculations

I concur with the prior reviewer that this is a significant issue. If the plots were well distributed in space this would be less of a concern, but the locations of plots are highly clustered. The authors attempted to respond by arguing that any bias introduced is small, but I confess either I am not understanding their argument clearly or it is not sufficient. In either case, it needs to be clarified.

In the previous review, we compared two methods for estimating biomass: one using the general South American height equation from the BIOMASS package (since we do not have a specific tree height equation for our region), and the other based on DBH, which incorporates climate variables under the hood. The biomass estimates from both methods were 99% correlated, indicating strong covariation. However, their means differed, with the method using height overestimating biomass by around 20%. The variation around the mean was large, and this inspection was not statistical. This discrepancy arose because we used DBH at the quadratic mean (DBHq), as some of our data only included DBHq-level data. Therefore, we had to use it for all sites. Using the height equation directly likely accentuated this bias. In addition, the biomass estimated using the estimated height was more correlated with climate than the one using the E parameter. Thus, we opted to retain the equation proposed by the BIOMASS package in the absence of height data.

Modeling framework:

Mixed effect models: The authors response appears appropriate, and I concur it is an adequate used of a mixed model as I understand their use (Line 585). Random

effects to address the repeated measures makes sense. I strongly suggest the authors expand on the statistical structure of the mixed effect model in the text, however, as I would be confused without the response to reviewer text on their use of the framework.

Response: We added this explanation in the methods:

"To account for the non-independence of repeated observations within sites over time (e.g., multiple censuses and intervals per site), we included random intercepts for site in all models. This approach accounts for temporal autocorrelation and site-level variation by allowing each site to have its own baseline, thus controlling for within-site dependence inherent to long-term forest monitoring."

And added the random effects notation in the model formulas presented in the methods.

Spatial clustering:

I again share the first reviewers concern. The authors argue that because spatial autocorrelation (SAC) was not detected, there is no significant concerns to be had. I disagree – it is an indication of model fit in geographic space, but hardly conclusive evidence that SAC doesn't matter for the conclusions. Spatial issues arise from design, and cannot always be addressed with statistical methodologies or checked via statistics. I sympathize with the authors, as they did not have control over plot locations for this study, but it is still an issue.

Response: We agree that there is a limitation in the spatial sampling design due to clustering. However, although the sampling is clustered, the areas cover a large spatial distance, as Brazil is a continental-sized country encompassing different forest types, environmental conditions, and evolutionary histories. Moreover, there is significant heterogeneity within the four spatial clusters, including variation in forest types, soil, and climate.

Our conclusions are therefore restricted to the regions of these forests (which are large, given Brazil's size) and not to the entire continuum between them, which we will acknowledge and clarify further in the text. Thus, the issue is not spatial autocorrelation (SAC) itself, but rather the sampling design. The goal of testing SAC in our models is to evaluate whether model inference is affected by SAC. When the test does not detect SAC, we can interpret that the bias from spatial autocorrelation in the residuals is minimal, and the model remains valid.

However, this does not address the issue of design and clustering, meaning the model is only valid within the known space of X (the range of predictor values the model was exposed to) and cannot be extrapolated to unsampled areas between distant clusters. We will explicitly recognize this limitation in the text. While we understand that statistical tests for SAC do not resolve design-related issues or the extrapolation of inferences, we consider them the best available approach for evaluating the model's validity, which remains conditional on the known space of X.

One option presented was using a “space” variable to address the clustering. The authors dismiss this, noting that it would also be correlated with climate (“this variable is the synthesis of the space itself, so it would be, in essence, the space.”). However I think they missed the first reviewers point, which was to drop the random effect for the plots and then use the random effect for the cluster. I agree with the authors this would be inappropriate, now that they have clarified their initial use of the random effect structure.

Response: Yes, the random effect of plot is required in our case because of the sampling design.

But we cannot dismiss the spatial arrangement of clusters. Any number of factors NOT in the model but which ARE correlated in space (like a disturbance event, or a past pest outbreak, or anything else) could EASILY have caused the patterns seen and not be considered. How much variance in their response metrics is explained simply by the cluster locations? Is it comparable to those explained by climate, for example? They are obviously correlated, so this itself does not say it is NOT climate, but it does suggest it could be something else distributed similarly in space to climate (in the case of just four clustered areas, this could be as simple as a 100 year prior wind disturbance that would not be detected per their methods).

Response: we used kmeans to find the four spatial clusters:

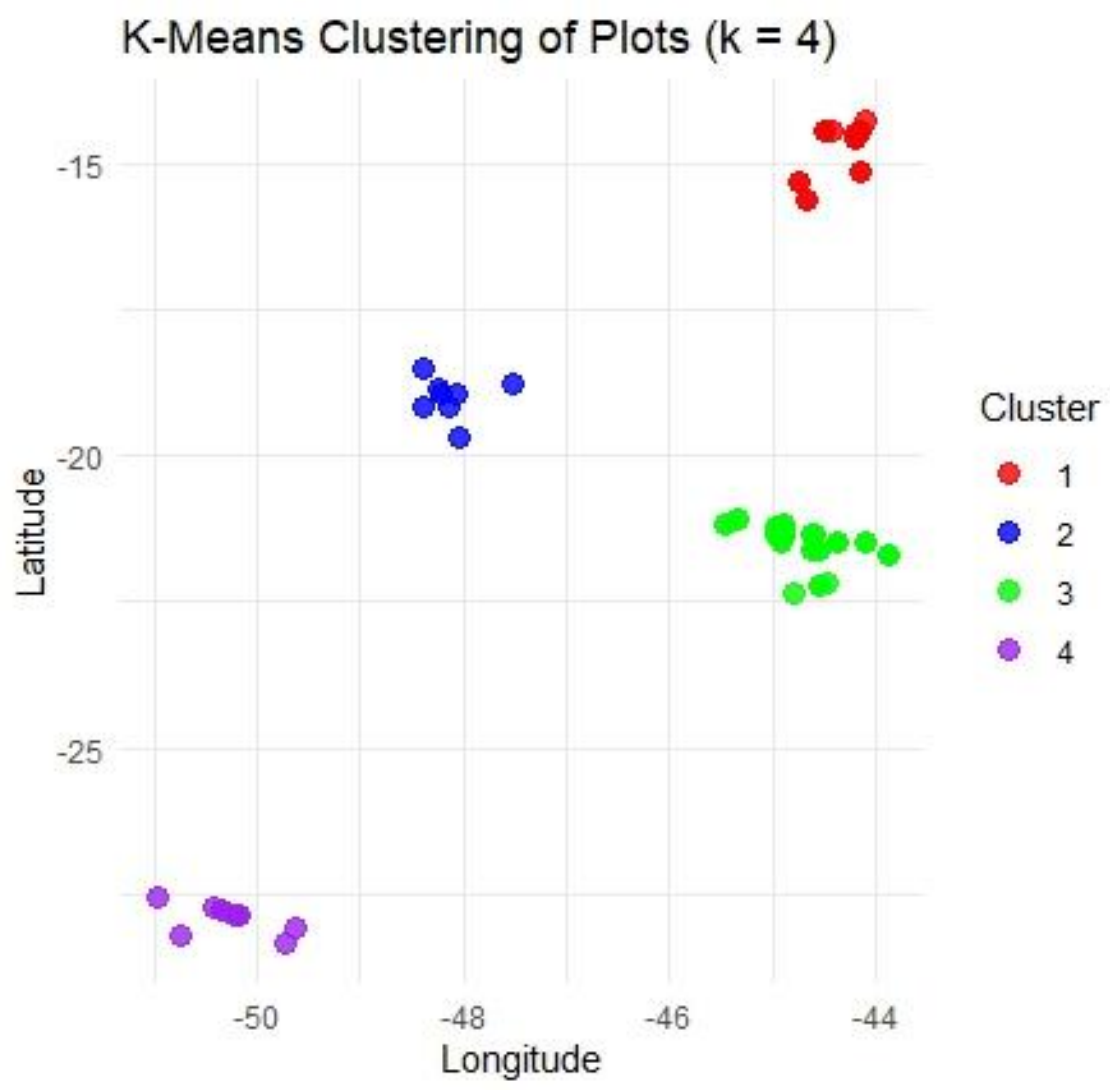

As mentioned in previous responses, disturbance and successional stages are not expected to be correlated with spatial patterns. For example, we fitted two global models for each carbon variable: one with climate as we already have, and one with the cluster class in place of climate. The average difference in R^2 between model comparisons for each carbon variable was 2%; for some variables, climate was better, and for others, the cluster model performed better. This small difference is likely to be numeric, as it is too small and not consistent across all carbon response variables.

Additionally, we fitted a random forest model to predict the cluster (as classes) using climate variables, and the model achieved 100% accuracy on unseen test data. This further supports the conclusion that there is unlikely to be a confounding variable, such as disturbance, correlated with our predictors, as the climate variable alone has the same explanatory power as the cluster variable.

In addition, the absence of SAC in models residuals also indicates that there is not likely to be a variable correlated with cluster/space that is not in the model.

One potential solution (though not ideal, and perhaps not even appropriate, as the authors know their data best) would be to include an interaction term between the site and cluster as a random effect. Perhaps this is incorporated, but the equations and the random effect structure is not clear in the manuscript methods. There are other options. But as it stands, it is difficult to support the authors conclusions without major questions about other factors not considered. I note that reviewer 3 also noted this, with the same response from the authors.
Response: We clarified the random effects structure in the manuscript to ensure that the model specifications are fully explained as showed in an answer above.

As stated above, including both climate and cluster in the same model led to exploded VIFs, as they are extremely correlated. Therefore, we chose to retain climate in the model, as it varies within and across clusters, given that the regions are large and heterogeneous.

We added this to the methods section:

"We tested the inclusion of spatial cluster as a predictor in the models. However, the strong association between cluster and climate due to the broad latitudinal gradient captured by the clusters resulted in extremely high VIF in models where cluster and climate were both included as predictors. Hence, we opted for the inclusion of climate instead of spatial cluster for two main reasons: first, for being a tangible, continuous variable instead of an arbitrary, discrete variable; second, for capturing within-cluster climate variation."

In a later comment, the original reviewer noted that the authors don't really have 49 plots. The authors noted there are 49 data points, aggregated from smaller plots. A separation of 1km in a forest is almost nothing in the context of these explanatory variables. The large distance for some plots noted (>1000km) is not really useful either, since the clusters are the key concern. I agree with the reviewer in the spatial sense; it appears to be primarily four clustered units at the broad scale. Given the many metrics that are correlated with space, it seems difficult to argue the 49 plots represent 49 truly independent observations statistically speaking.
Response: The maximum distance between sites is 1,695 km, which represents 38% of Brazil's maximum north-south extent. Within these regions, there is considerable environmental heterogeneity even at smaller spatial scales, including variation in soil, topography, and proximity to rivers. While site composition differs even at distances of less than 5 km, most sites are not in close proximity to one another. The table below demonstrates that the average distance between sites within a cluster is approximately 60 km, with cluster ranges around 150 km. This aligns with regional-scale patterns, supporting the idea that each cluster represents its respective region while the set of clusters captures broader-scale spatial patterns (>1,000 km).

cluster	mean_dist_km	max_dist_km
1	66.46637	161.0001
3	66.35789	175.5617

4	60.59310	148.1125
2	60.96309	136.2190

Since spatial autocorrelation (SAC) is absent in model residuals, the presence of nearby sites (<1 km or 10 km, for example) does not invalidate model estimates or p-values. However, as we noted earlier, while the analysis is numerically sound, our inferences remain constrained to the sampled regions rather than the entire environmental gradient.

We added this to the methods section:

"Sites within the same cluster have an average maximum distance of ~150 km and an average distance of ~60 km. Such distribution characterises large-scale variations (between clusters) and regional variations (within clusters). Despite the spatial proximity between some of the sites (< 5 km), there is high local-scale environmental heterogeneity within the clusters, driven by soil, topography and proximity to rivers and streams. Hence, we can assume independence between the sites, since their climatic and compositional similarities will have been incorporated by the model predictors (i.e., climate and functional composition)."

"we tested for spatial autocorrelation in model residuals with Moran's I. The results indicated no significant spatial autocorrelation, suggesting that spatial dependencies among observations did not bias model inferences."

Large number of parameters:
The authors have simplified the equations. However the structural issues still exist as noted above.

Response: we acknowledge that our models include a large number of parameters. However, there is an inherent dilemma between model simplicity—which is desirable—and ensuring that important variables are not omitted. Excluding key variables could lead to incorrect conclusions, while including all relevant variables is necessary to properly test our hypotheses. Given that our models are unlikely to be overfitted (with a maximum R^2 below 0.5), we believe that retaining important variables is the best approach to minimize confounding effects and ensure accurate interpretations. To further balance complexity and robustness, our use of multimodel inference—where results are derived from a combination of submodels—ensured that each submodel was fitted with a parameter-to-observation ratio of at least 1:10. In this case, we prefer the risk of

slight overfitting, if it at least allows us to correctly describe the dataset, over the risk of omitting crucial variables, which could lead to inadequate modeling of both the data and the broader population as the omitted variables are confounding variables that needs to be controlled.

Relationships and careful interpretation:
The use of GAM's is highly flexible and always should be interpreted with caution. And why not GAMM's?

Response: we agree with the caution advised when using GAMs due to their high flexibility. We opted for GAMs in this case simply because time is the only predictor, and our interest lies in capturing oscillations over time to describe the average temporal behavior. While the flexibility of GAMs is real and becomes more pronounced with a large number of predictors or incorrect hyperparameter settings, in our case, the curves are not overly wiggly. Most of them appear almost linear, and the oscillations are smooth and consistent with the data. Additionally, the GAM function supports random effects, such as s(plot_code, bs = "re"). For cases with just one random effect, a GAM can be preferred over a GAMM due to its simplicity and the fact that both methods typically yield the same results.

Overall the statistics are written up in a very confusing manner. Several places are missing equations (line 633).

Response: We improved the equations.

Alternative hypotheses are not formulated or tested. The net effect of the lack of clarity and consideration of potential alternative explanations - exemplified by the spatial structure concerns - means the reader is not convinced that the conclusions drawn are valid. Perhaps this could be addressed with simply more language around the limitations, but they are substantial and mostly stem from the underlying design of the plot network and the apparent lack of key covariates needed to dismiss alternative hypotheses for these particular conclusions.

Response: We appreciate the reviewer's thoughtful comment and recognize the importance of considering alternative explanations. In the revised manuscript, we explicitly acknowledged the limitations of our study and present alternative hypotheses where appropriate.

As discussed in previous responses, disturbance history — a key potential confounding factor — is not expected to be spatially structured across our study

area. This expectation is based on the choice of the areas when sampling, field observations, and regional knowledge indicating homogeneous disturbance regimes across the sampled areas, thus, the relationships we found are not likely to being affected by this confounding variable.

We also clearly stated that generalizations from our study should be limited to the regions represented by the identified clusters and not extrapolated across the full environmental gradient. The samples are clustered in broad, spatially separated regions that encompass large and heterogeneous areas. Cluster identity and climate variables are strongly correlated; climate was selected for analysis because it better captures variation within clusters and varies even between geographically close areas, providing additional resolution. As the clusters span a wide range of climatic conditions across distant regions, the plot network also provides valuable large-scale information.

As is common in biological and observational studies, especially those involving long-term temporal data, not all potential influencing factors can be controlled. However, although we do not have access to all possible confounding variables, our model is already complex and incorporates many relevant predictors.

Following the reviewer's suggestion, we strengthened the language around these limitations. We emphasized that, while the plot network and long-term data provide valuable insights across large spatial and environmental scales, care must be taken when extrapolating conclusions beyond the regions represented by the clusters and that the key confounding variables are not likely to be correlated with space/climate. With these clarifications, we believe the robustness of our findings and the scientific value of the dataset will be more clearly communicated.

These sentences were added to the manuscript to address these limitations:

"The forests are at advanced successional stages, as the sites were specifically selected to represent areas with minimal human intervention, favoring the most conserved locations (thus, disturbance and forest age are not expected to be correlated with space and environmental conditions). They have not experienced the effects of fire, landslides, floods, or logging during the monitoring period or prior to the first census."

"Although disturbance and forest age are expected to be spatially homogeneous in our samples based on the sampling criteria and historical data, successional processes cannot be completely disregarded. Thus, considering that successional processes are controlled in our samples,"

“We also acknowledge the need for precise measures of disturbance history and forest age to better understand the carbon sink dynamics in these regions. However, our sampling strategy was designed to represent the most conserved and least disturbed forests, so disturbance and age are unlikely to confound the observed relationships. This supports the reliability of our findings, although the role of disturbance in these forests warrants further investigation.”

“Sites within the same cluster have an average maximum distance of ~150 km and an average distance of ~60 km. Such distribution characterises large-scale variations (between clusters) and regional variations (within clusters). Despite the spatial proximity between some of the sites (< 5 km), there is high local-scale environmental heterogeneity within the clusters, driven by soil, topography and proximity to rivers and streams. Hence, we can assume independence between the sites, since their climatic and compositional similarities will have been incorporated by the model predictors (i.e., climate and functional composition).”

“we tested for spatial autocorrelation in model residuals with Moran’s I. The results indicated no significant spatial autocorrelation, suggesting that spatial dependencies among observations did not bias model inferences.”

“We tested the inclusion of spatial cluster as a predictor in the models. However, the strong association between cluster and climate due to the broad latitudinal gradient captured by the clusters resulted in extremely high VIF in models where cluster and climate were both included as predictors. Hence, we opted for the inclusion of climate instead of spatial cluster for two main reasons: first, for being a tangible, continuous variable instead of an arbitrary, discrete variable; second, for capturing within-cluster climate variation.”

“In addition, as our samples are spatially clustered, caution is needed when extrapolating our conclusions to the regions between the large-scale clusters.”

This is not meant to dismiss the value of the plot network nor the amount of work the authors have done. These long-term networks are incredibly important. But they have limitations in what they can tell us, especially when the history of the plot location is not known. It can be easy to come to the incorrect conclusions if

key variables are not included (or indeed, if one doesn't know to include those variables because of that lack of history).

We appreciate the reviewer's acknowledgment of the value of long-term plot networks and the work involved in maintaining them. We fully agree that the lack of historical context can pose limitations in interpreting patterns and trends. In response to this important point, we have addressed these concerns in detail in our response letter and clarified them throughout the revised manuscript. These clarifications aim to provide a balanced perspective on the strengths and limitations of our study and ensure transparency in the interpretation of our findings.
